# Morphogen-induced kinase condensates transduce Hh signal by allosterically activating Gli

**DOI:** 10.1126/sciadv.adq1790

**Published:** 2025-01-10

**Authors:** Yuhong Han, Mengmeng Zhou, Bing Wang, Jin Jiang

**Affiliations:** ^1^ Department of Molecular Biology, University of Texas Southwestern Medical Center, Dallas, TX 75390, USA.; ^2^ Department of Pharmacology, University of Texas Southwestern Medical Center, Dallas, TX 75390, USA.

## Abstract

Hedgehog (Hh) morphogen governs embryonic development and tissue homeostasis through the Ci/Gli family transcription factors. Here we report that Hh induces phase separation of the fused (Fu)/Ulk family kinases to allosterically regulate Ci/Gli. We find that Hh-induced phosphorylation of Fu/Ulk3 promotes SUMOylation of their inverted phosphorylation-dependent SUMOylation motifs. Subsequent interaction between SUMO and SUMO-interacting motif drives Fu/Ulk3 self-assembly to form biomolecular condensates that recruit Ci-Sufu and Gli-Sufu in the cytoplasm and primary cilium, respectively. Within the condensates, Fu/Ulk3 undergoes a conformational change to expose Ci/Gli for Fu/Ulk3-mediated phosphorylation and activation, leading to gradual accumulation of nuclear Ci^A^/Gli^A^ transcriptional complexes in proportion to ligand dose and exposure time. Our findings provide mechanistic insights into the spatiotemporal control of Hh signal transduction, reveal previously unexplored regulatory mechanism and function for biomolecular condensation, and establish a paradigm for kinase-mediated signal transduction whereby a kinase allosterically activates its substrate through ligand-induced and condensation-driven conformational change.

## Teaser

Hedgehog induces phase separation and conformational change of Fu/Ulk family kinases to activate pathway transcription factors.

## INTRODUCTION

Hedgehog (Hh) signaling is a major developmental pathway that governs embryonic development and adult tissue homeostasis in species ranging from insects to mammals (*1*, *2*). Aberrant Hh pathway activity has been linked to a wide range of human disorders including birth defects and cancer (*3*, *4*). Hh morphogen controls pattern formation in a concentration and time-dependent manner through a largely conserved signal transduction cascade that culminates in the conversion of the latent transcription factors Ci/Gli from transcriptional repressors (Ci^R^/Gli^R^) to activators (Ci^A^/Gli^A^) (*1*, *5*–*7*).

In *Drosophila*, genetic studies revealed that *fused* (*fu*), which encodes a Ser/Thr kinase, is required for converting full-length Ci (Ci^F^) into Ci^A^ (*8*, *9*); however, the biochemical mechanism by which Fu activates Ci has remained poorly understood. Fu forms a complex with the kinesin-like protein Costal2 (Cos2) that recruits multiple kinases including protein kinase A (PKA) to phosphorylate Ci, targeting it for Slimb-mediated proteolytic processing into Ci^R^ in quiescent cells (*10*–*15*). Upon Hh stimulation, the Hh signal transducer Smoothened (Smo) interacts with Cos2 and PKA to inhibit Ci processing (*13*, *16*–*21*). However, inhibition of Ci processing is insufficient for Ci activation because the accumulated Ci^F^ is bound and inhibited by Sufu (*22*, *23*). High levels of Hh induce clustering of Smo C-terminal intracellular tails, which in turn promotes dimerization/oligomerization of the bound Cos2/Fu complexes, leading to Fu trans-autophosphorylation and kinase activation (*24*–*28*). Activated Fu phosphorylates Ci at multiple sites, priming CK1-mediated phosphorylation at adjacent sites, which promotes Ci activation by attenuating Sufu binding (*29*–*31*).

In vertebrates, Hh signal transduction occurs at primary cilia, which are microtubule-based membrane protrusions found in most mammalian cells (*32*). Upon Hh stimulation, key pathway components including Smo, Gli, and Sufu translocate to primary cilia (*33*–*38*), leading to the hypothesis that Gli proteins are converted into Gli^A^ at primary cilia. Recent studies revealed that Gli proteins have conserved phosphorylation sites that mediate the activation of Gli2, a major contributor of Gli^A^ activity in mammals, and that phosphorylation of Gli2 is mediated by the Fu/Ulk family kinases Ulk3 and Stk36 dependent on Gli2 ciliary localization (*29*–*31*). How Gli proteins are activated at primary cilia has remained enigmatic.

Membraneless organelles or biomolecular condensates have been implicated in many cellular processes (*39*–*41*). The formation of biomolecular condensates through phase separation is often constitutive or regulated in response to stress signals; however, whether phase separation can be induced by extracellular signals has been largely underexplored. In general, biomolecular condensates function to facilitate biochemical reactions by simultaneously concentrating substrates and enzymes in the same subcellular compartments or to suppress the activity of the sequestered factors that are otherwise active outside the condensates (*39*, *40*). Whether biomolecular condensates can fulfill other function has remained unexplored. Here, we report that Hh induces Fu/Ulk3 phase separation to form biomolecular condensates that recruit Sufu and Ci/Gli2. Fu/Ulk3 phase separation is induced by phosphorylation-dependent SUMOylation and SUMO–SUMO-interacting motif (SIM) interaction of their regulatory domains. Whereas Fu-Sufu-Ci condensates form in the cytoplasm, Ulk3-Sufu-Gli2 condensates form at the ciliary tip. We provide evidence that Fu/Ulk3 condensation drives their conformational change (a process we called maturation) to allosterically expose Ci/Gli for Fu/Ulk3-mediated phosphorylation and activation and that nuclear Ci^A^/Gli^A^ exists in a complex containing phosphorylated Ci/Gli, mature Fu/Ulk3, and Sufu. Our study reveals a unified mechanism for Hh signal transduction from *Drosophila* to mammals despite their differential requirement for primary cilia.

## RESULTS

### Hh-induced phosphorylation of Fu regulatory domain confers a noncatalytic function

**Fig. 1.**
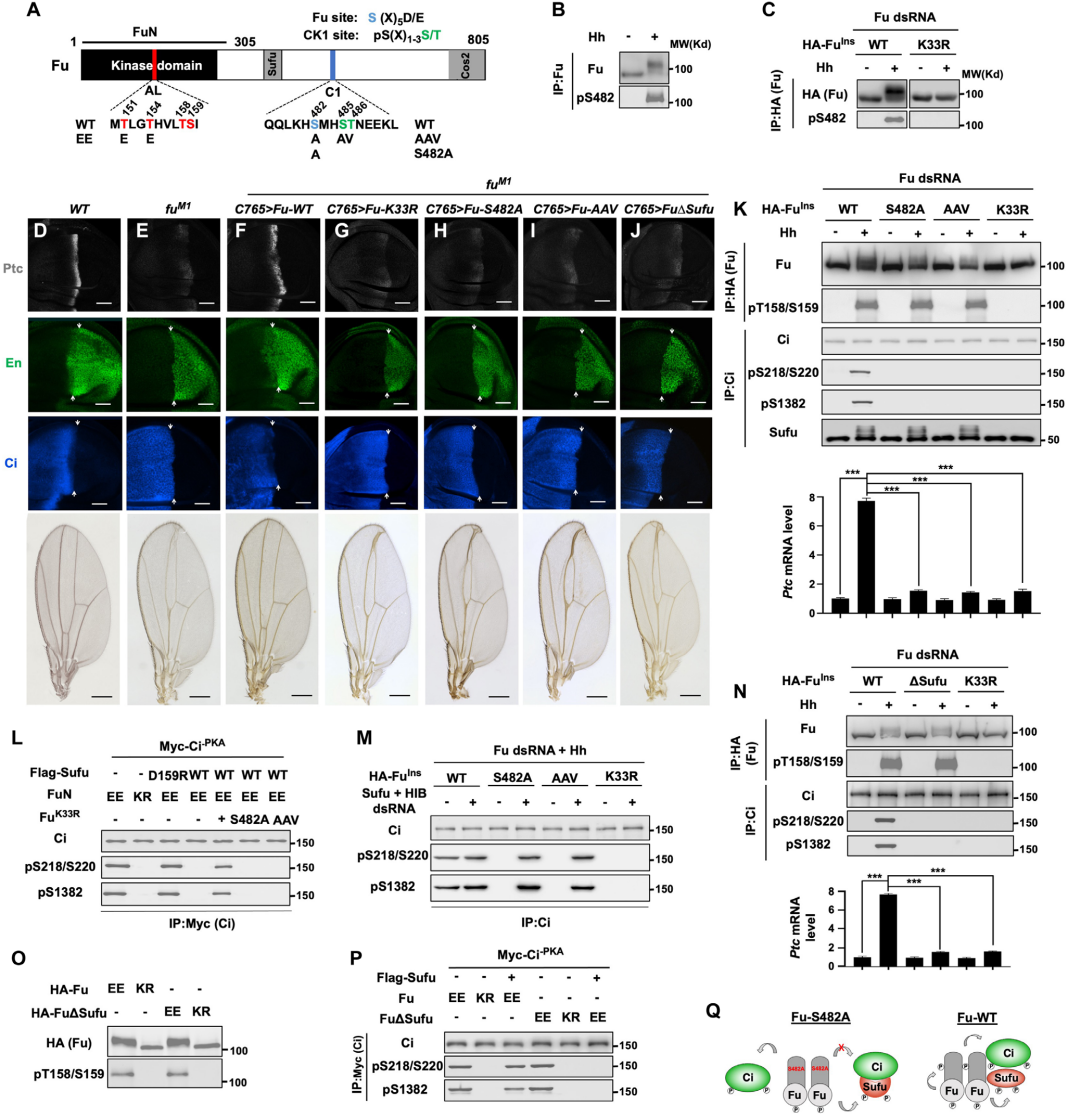
Hh-induced phosphorylation of FuR confers a noncatalytic function to Fu. (**A**) Schematic drawing of Fu with Sufu- and Cos2-binding regions (gray boxes), activation loop (AL), and the C-terminal regulatory region (C1, blue bar) indicated. (**B**) Western blot (WB) analysis of the phosphorylation of endogenous Fu in Cl8 cells treated with Hh-conditioned or control medium. (**C**) WB analysis of the phosphorylation of RNAi-insensitive (Ins) WT or kinase dead (K33R) Fu stably expressed in Cl8 cells treated with Fu dsRNA and Hh-conditioned or control medium. (**D** to **J**) Expression of Ptc, En, and Ci in late third instar wing imaginal discs or adult wings of the indicated genotypes. Scale bars, (wing discs) 50 μm and (adult wings) 500 μm, respectively. (**K**) WB analysis of the phosphorylation of Fu, Ci, and Sufu proteins (top) and qRT-PCR analysis for *ptc* expression (bottom) in Fu-depleted Cl8 cells stably expressing the indicated Fu constructs. (**L**) WB analysis of the phosphorylation of Ci^-PKA^ in S2R^+^ cells cotransfected with the indicated Sufu and Fu constructs. (**M**) WB analysis of Ci phosphorylation in Fu-depleted Cl8 cells stably expressing the indicated Fu constructs and treated with control or Sufu/HIB dsRNA. (**N**) WB analysis of Fu and Ci phosphorylation (top) and qRT-PCR analysis of *ptc* mRNA (bottom) in Fu-depleted Cl8 cells stably expressing the indicated Fu constructs. (**O**) WB analysis of the phosphorylation of the indicated Fu variants in S2R^+^ cells. (**P**) WB analysis of the phosphorylation of Ci^-PKA^ in S2R^+^ cells cotransfected with the indicated Fu and Sufu constructs. (**Q**) Fu-S482A can phosphorylate Sufu and free Ci but not Sufu-bound Ci (left). Phosphorylation of Sufu-bound Ci depends on S482 phosphorylation as well as Fu-Sufu interaction (right). Data are means ± SD from three (K) or two (P) independent experiments. ***P < 0.001 (*t* test).

Our previous studies identified three Fu phosphorylation sites on Ci that fall into the consensus sequence: S/T(X)_5_D/E (*29*–*31*). To determine how phosphorylation on these sites affects Ci activation, we converted the Fu and adjacent CK1 phosphorylation sites into acidic residues in a Ci variant (Ci^-PKA^) in which three PKA sites were mutated to block Ci processing (*29*, *42*). Although the resulting construct Ci^-PKA_SD123^ is more active than Ci^-PKA^ when expressed in wing imaginal discs at close to endogenous level (*31*), full activation of Ci^-PKA_SD123^ still depended on Hh and Fu (fig. S1A), suggesting that Fu may phosphorylate additional site(s) on Ci or another substrate(s), or use a noncatalytic mechanism to fully activate Ci. A previous study revealed that the regulatory domain (FuR) of Fu contains a CK1 phosphorylation cluster (S485/T486) primed by S482 phosphorylation and that mutating this phosphorylation cluster prevented the constitutive activity of GAP-Fu (Fig. 1A) (*27*, *29*). Our in vitro kinase assays showed that Fu could phosphorylate S482, which primed CK1 to phosphorylate S485 and T486 (fig. S1, B and C). Previous studies showed that forced dimerization of Fu through a coiled-coil (CC) domain from GCN4 (CC-Fu) is sufficient to induce its kinase activation (*26*, *28*). Using an antibody that recognizes phosphorylated S482 (pS482), we found that wild-type (WT) CC-Fu but not its kinase dead form (K33R) phosphorylated S482 when expressed in S2 cells (fig. S1D). Furthermore, Hh induced phosphorylation of endogenous Fu at S482 dependent on Fu kinase activity in Cl8 cells (Fig. 1, B and C), demonstrating that Hh induces Fu autophosphorylation at S482.

To assess the functional significance of FuR phosphorylation, we first carried out a cell-based derepression assay in which the repression of exogenously expressed Ci by coexpressed Sufu is released by cotransfection of active Fu constructs (*26*, *29*). S2 cells with endogenous Fu depleted by Fu double-stranded RNA (dsRNA) were transfected with Ci^-PKA^, Sufu, RNA interference (RNAi)–insensitive (Ins) WT or mutant CC-Fu carrying K33R, S482A, or AAV mutations (Fig. 1A), and a *ptc-luciferase* (*ptc-Luc*) reporter gene. As shown in fig. S1E, only WT, but not K33R, S482A, or AAV version of CC-Fu alleviated Sufu-mediated repression of Ci^-PKA^. Next, we generated transgenic flies expressing Myc-tagged WT or mutant (S482A, AAV, or K33R) Fu under the control of a *UAS* promoter and determined whether they could rescue the phenotypes caused by an *fu* null mutation (*fu^M1^*) (*27*). A weak Gal4 driver, *C765*, was used to drive the *UAS* transgene expression at low levels to avoid overexpression (*43*). In wing imaginal discs, posterior (P) compartment cells express Hh, whereas anterior (A) compartment cells express Ci; consequently, A-compartment cells near the A/P boundary respond to Hh and activate Hh target genes including high-threshold responsive genes *ptc* and *engrailed* (*en*)(Fig. 1D) (*8*, *44*, *45*). In *fu^M1^* discs, *ptc* expression near the A/P boundary was diminished, although its expression domain became broader because of more Hh spreading into the anterior compartment (Fig. 1E) (*46*). In addition, *en* expression in A-compartment cells abutting the A/P boundary, which requires the peak levels of Hh, was abolished (Fig. 1E). Consistent with Fu being required for converting Ci^F^ into labile Ci^A^ in response to high levels of Hh (*8*), full-length Ci was up-regulated in A-compartment cells abutting the A/P boundary in *fu^M1^* discs (Fig. 1E). Expression of Fu^WT^ (*C765 > Fu^WT^*) in *fu^M1^* wing discs restored Hh target gene expression and down-regulated Ci in A-compartment cells near the A/P boundary (Fig. 1F and fig. S1F), suggesting that *C765 > Fu^WT^* converted Ci^F^ into labile Ci^A^ in the absence of endogenous Fu. In addition, *C765 > Fu^WT^* fully rescued the “fused” wing phenotype in *fu^M1^* adults (Fig. 1F). By contrast, expression of Fu^K33R^, Fu^S482A^, or Fu^AAV^ failed to rescue the *fu* mutant phenotypes (Fig. 1, G to I). These findings demonstrate that Hh-induced phosphorylation of FuR is essential for high-threshold Hh responses mediated by Ci activation.

Fu activates Ci by phosphorylating multiple sites in Ci including S218 and S1382, and S218 phosphorylation primes CK1 to phosphorylate S220 (*29*, *31*). These phosphorylation events can be detected by phospho-specific antibodies pS218/220 and pS1382, respectively (*29*, *31*). In Fu-depleted S2 cells transfected with Ci^-PKA^, Sufu and RNAi-insensitive CC-Fu constructs, CC-Fu^WT^ but not CC-Fu^AAV^ or CC-Fu^K33R^ promoted Ci^-PKA^ phosphorylation at S218/220 and S1382 (fig. S1G). However, unlike CC-Fu^K33R^, CC-Fu^AAV^ could still phosphorylate its own activation loop as determined by the phospho-specific antibody pT158/S159 (fig. S1G) (*26*), as well as the coexpressed Cos2 and Sufu as indicated by their mobility shift (fig. S1H), suggesting that CC-Fu^AAV^ is catalytically active. To determine whether FuR phosphorylation could be involved in Hh-induced Fu kinase activation, we generated Cl8 cells stably expressing RNAi-insensitive Fu^WT^, Fu^S482^, Fu^AAV^, or Fu^K33R^ at levels close to that of endogenous Fu (fig. S1I). After depletion of endogenous Fu by dsRNA, these cell lines were treated with Hh-conditioned medium. Hh induced phosphorylation of Fu (pT158/S159), Sufu (indicated by mobility shift) and Ci (pS218/S220 and pS1382), as well as *ptc* expression in cells expressing Fu^WT^ but not in cells expressing Fu^K33R^ (Fig. 1K). However, in cells expressing either Fu^S482A^ or Fu^AAV^, Hh could still induce phosphorylation of Fu and Sufu but failed to induce Ci phosphorylation or *ptc* expression (Fig. 1K). These results demonstrate that S482A and AAV mutations abolish Ci phosphorylation and activation without affecting the enzymatic activity of Fu kinase.

The above observations imply that Ci activation may require a noncatalytic function of Fu promoted by FuR phosphorylation. In cell-based derepression assays, a phospho-mimetic and constitutively active form of Fu [Fu^EE^, (*22*)] could activate Ci^-PKA^ regardless the presence or absence of endogenous Fu (fig. S2A). However, Fu^EE^ bearing the AAV mutation (Fu^EE/AAV^) could only activate Ci^-PKA^ in control S2 cells but not in Fu-depleted cells (fig. S2A). Coexpression of the kinase dead Fu^K33R^ complemented with Fu^EE/AAV^ to restore Ci^-PKA^ activation in Fu-depleted cells in a manner similar to Fu^WT^ (fig. S2A), suggesting that Fu^K33R^ may provide a noncatalytic activity that is lost in Fu^EE/AAV^. Both S482A and AAV mutations abolished the noncatalytic function of Fu^K33R^ (fig. S2A). In a parallel experiment, both Fu^WT^ and Fu^K33R^ but not Fu^K33R/S482A^ complemented with a constitutively active Fu kinase domain (FuN^EE^) to restore Ci^-PKA^ activation in Fu-depleted cells (fig. S2B). In addition, a previous study showed that a constitutively active Fu kinase domain (Fu-EE 1-305) could rescue Hh pathway activity in *fu^mH63^* wing discs that expressed a kinase-inactive Fu (*27*). Together, these results suggest that Fu contains two separable activities: a kinase activity stimulated by its activation loop phosphorylation and a noncatalytic activity promoted by FuR phosphorylation, both of which are required for Ci activation.

### The noncatalytic function of Fu is required for unmasking Ci in the Sufu-Ci complex

The N- and C-terminal Fu phosphorylation sites on Ci (S218 and S1382) are located within or close to the N- and C-terminal Sufu-binding domains, respectively (fig. S2C) (*23*, *29*), raising the possibility that Sufu binding could mask these sites. Addition of Sufu in in vitro kinase assays abolished phosphorylation of glutathione *S*-transferase (GST)–Ci_215–261_ and GST-Ci_1372–1397_ by FuN^EE^ (fig. S2D). However, phosphorylation of GST-Ci_215–227_, which lacks a critical Sufu-binding motif, SYGH (*47*), was unaffected (fig. S2D). Furthermore, coexpression of WT but not a Ci-binding–deficient Sufu (D159R) blocked Ci^-PKA^ phosphorylation by FuN^EE^ in S2 cells (Fig. 1L). This blockage was alleviated by coexpression of Fu^K33R^ but not by Fu^K33R/S482A^ or Fu^K33R/AAV^ (Fig. 1L), suggesting that Fu^K33R^ provided a noncatalytic activity to expose S218/S220 and S1382 masked by Sufu binding. To further test this notion, we asked whether removing endogenous Sufu would allow Fu^S482A^ and Fu^AAV^ to phosphorylate endogenous Ci. After depletion of endogenous Fu in Cl8 cells stably expressing RNAi-insensitive Fu constructs, only Fu^WT^ but not Fu^S482A^ or Fu^AAV^ could phosphorylate S218/S220 and S1382 in response to Hh (Fig. 1M). However, depletion of Sufu allowed Fu^S482A^ and Fu^AAV^ to phosphorylate Ci (Fig. 1M), suggesting that both Fu^S482A^ and Fu^AAV^ have normal kinase activity that can phosphorylate unbound Ci. These results suggest that FuR phosphorylation is required for unmasking Ci in the Sufu-Ci complex likely through an allosteric mechanism that alters Ci/Sufu interaction. Of note, Hh-induced MATH and BTB domain containing protein (HIB) was knocked down together with Sufu to stabilize Ci because unbound Ci is labile because of its degradation by HIB (*48*).

One would predict that allosteric regulation of Sufu-Ci by Fu requires physical interaction between Fu and the Sufu-Ci complex. Deleting the Sufu-binding domain (amino acids 371 to 387) (*49*) from Fu^EE^ (Fu∆Sufu^EE^) prevented it from phosphorylating Ci^-PKA^ in S2 cells in the absence of endogenous Fu when Sufu was coexpressed, although Fu∆Sufu^EE^ had normal kinase activity (Fig. 1, O to P). Furthermore, RNAi-insensitive Fu∆Sufu expressed at endogenous level (fig. S1I) neither phosphorylated nor activated endogenous Ci, although its kinase activity was stimulated by Hh (Fig. 1N). Expression of Fu∆Sufu failed to rescue the *fu* null mutant phenotypes (Fig. 1J). Hence, Fu kinase activation and Ci phosphorylation/activation are two separable steps with Ci phosphorylation/activation being regulated by an allosteric mechanism dependent on S482 phosphorylation and physical interaction between Fu and Sufu (Fig. 1Q).

### Hh promotes Fu conformational maturation via phosphorylation of its regulatory domain

**Fig. 2.**
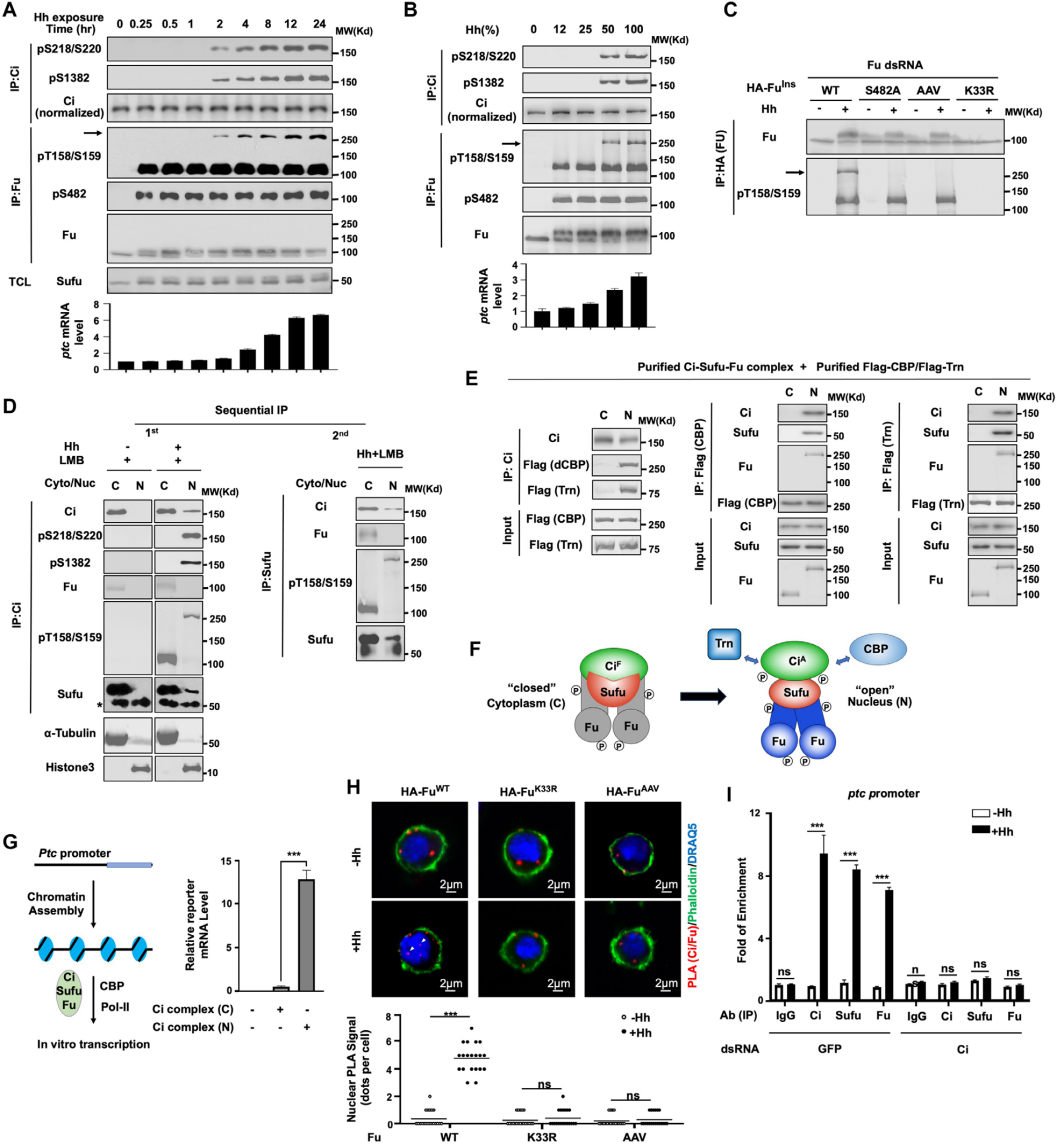
Phosphorylation of Fu regulatory domain promotes a conformational change of Fu to allosterically activate Ci. (**A**) WB analysis of Fu and Ci phosphorylation (top) and qRT-PCR of *ptc* mRNA (bottom) in Cl8 cells treated with Hh-conditioned medium for the indicated time. Arrow indicates the SDS-resistant Fu dimer. (**B**) WB analysis of Fu and Ci phosphorylation (top) and qRT-PCR of *ptc* mRNA (bottom) in Cl8 cells treated with increasing dose of Hh for 6 hours. (**C**) WB analysis of Fu phosphorylation and maturation in Fu-depleted Cl8 cells stably expressing the indicated Fu constructs and treated with control or Hh-conditioned medium. (**D**) WB analysis of cytoplasmic (C) and nuclear (N) Ci complexes isolated by sequential IP from Cl8 cells treated with Hh-conditioned or control medium. Cells were pretreated with 5 nM LMB for 30 min before harvesting for fractionation. Primary (first) and secondary (second) IP experiments were carried out with anti-Ci and anti-Sufu antibodies, respectively. Asterisk indicates IgG. (**E**) In vitro binding assay using cytoplasmic or nuclear Ci complexes immunopurified from Hh/LMB-treated Cl8 cells and *Drosophila* CBP and Trn purified from sf9 cells. (**F**) Diagram of cytoplasmic and nuclear Ci complexes. Of note, both the cytoplasmic and nuclear complexes may contain two copies of Ci/Sufu. (**G**) qRT-PCR analysis of in vitro transcription experiments using the cytoplasmic or nuclear Ci complexes immunopurified from Hh/LMB-treated Cl8 and chromatin-assembled *ptc* promoter fused to a reporter in the presence of CBP and Pol II. (**H**) Images (top) and quantification (bottom) of PLA of Ci and Fu in Fu-depleted Cl8 cells stably expressing the indicated Fu constructs. (**I**) ChIP qPCR analysis of chromatin-associated Ci, Sufu, and Fu on *ptc* promoter in control or Ci-depleted Cl8 cells. Data are means ± SD from three independent experiments. ***P < 0.001 (*t* test). ns, not significant.

We next asked how FuR phosphorylation mediates allosteric regulation of Ci. In a time-course experiment in which Cl8 cells were treated with Hh-conditioned medium for increasing amount of time, Fu kinase activation (indicated by pT158/S159 and pS482) was detected at 15 min, whereas Ci activation (indicated by pS218/S220, pS1382, and *ptc* expression) was detected at 2 hours upon ligand exposure (Fig. 2A). Ci phosphorylation and Ci^A^ activity increased progressively overtime and plateaued at 12 to 24 hours after Hh exposure. A high molecular weight Fu isoform whose size (~250 *K*_d_) was indicative of an Fu dimer was induced at 2 hours after Hh exposure, which coincided with the onset of Ci phosphorylation and activation (Fig. 2A). In the following, we refer to this SDS-resistant Fu dimer as “mature” form and the process of its formation as “Fu maturation” to distinguish from the process of Fu kinase activation.

When Cl8 cells were treated with conditioned medium containing different concentrations of Hh, low dose Hh (12%) was sufficient to induced Fu kinase activation, whereas high dose (>50%) was required to induce Fu maturation and Ci phosphorylation/activation (Fig. 2B). Overexpression of CC-Fu or Fu^EE^ in S2 cells also promoted Fu maturation (fig. S3, A and B). The ~250-*K*_d_ band could be converted into a single band corresponding to Fu monomers by prolonged SDS treatment (fig. S3B), suggesting that the ~250-*K*_d_ band corresponded to Fu dimers. Although the pT158/S159 antibody could recognize both the monomeric and dimeric Fu on SDS–polyacrylamide gel electrophoresis (SDS-PAGE) (Fig. 2, A and B, and fig. S3, A and B), the pS482 antibody only recognized the monomeric but not the dimeric form (fig. S3B). However, the pS482 antibody could recognize the monomeric Fu derived from the SDS-resistant dimer (fig. S3B), indicating that the pS482 epitope was masked in the mature Fu. Both S482A and AAV mutations abolished the formation of the SDS-resistant Fu dimer (Fig. 2C and fig. S3A), suggesting that Fu-R phosphorylation is essential for Fu maturation.

### Mature Fu forms a complex with Sufu-Ci to allosterically activate Ci

In unstimulated cells, Ci complexes containing Sufu and Fu were detected only in the cytoplasm; however, after Hh stimulation, a fraction of Ci-Sufu-Fu complexes was found in the nucleus when Ci nuclear export was blocked by leptomycin B (LMB) (Fig. 2D) (*50*, *51*). The existence of Ci, Sufu, and Fu in the same complexes was confirmed by sequential immunoprecipitation (IP) (Fig. 2D). Both the cytoplasmic and nuclear Ci complexes migrated to similar position (~600 *K*_d_) on blue native gels (BN-PAGE) that preserve the native composition of protein complexes (fig. S3C). Immunoblot analysis confirmed that both cytoplasmic and nuclear Ci complexes contained Ci, Sufu, and Fu, and their molecular weight on BN-PAGE suggests that they may contain two copies of each protein (fig. S3C). Consistent with Ci^A^ entering the nucleus, pS218/S220 and pS1382 recognized Ci in the nuclear but not in the cytoplasmic complexes (Fig. 2D and fig. S3C). The nuclear Ci complex contained the mature Fu, whereas Fu in the cytoplasmic Ci complex run as monomer on SDS PAGE (Fig. 2D). The pS482 antibody only recognized the cytoplasmic but not the nuclear Ci complexes on BN-PAGE (fig. S3C), lending further support that Fu in the nuclear and cytoplasmic Ci complexes existed in different conformation. IP experiments showed that nuclear but not cytoplasmic Ci-Sufu-Fu complexes were accessible to Transportin (Trn) and CREB-binding protein (CBP) binding (Fig. 2E). Limited proteolysis assay revealed that Sufu in the nuclear complex was more accessible to trypsin compared with that in the cytoplasmic complex (fig. S3D). Hence, in the presence of Hh and LMB, the cytoplasm appears to contain almost exclusively closed Ci-Sufu-Fu complexes, while the nucleus contains open complexes with unmasked pS482 epitope and Ci phosphorylation sites, increased Su(fu) accessibility to trypsin, and increased Ci accessibility to binding partners. Subsequent phosphorylation of Ci by Fu and CK1 may further stabilize the open conformation as phosphorylation reduces the binding affinity of Sufu to Ci (*29*, *31*).

In vitro transcription assay using a chromatin assembled *ptc*-*luc* fusion reporter as a template revealed that the nuclear Ci-Sufu-Fu complex was indeed transcriptionally active (Fig. 2G). Proximity ligation assay (PLA) (*52*) confirmed that Fu formed a complex with Ci in the nucleus of Hh-stimulated cells (Fig. 2H). Chromatin IP (ChIP) showed that Ci, Sufu, and Fu were enriched on the *ptc* promoter upon Hh stimulation and that the chromatin association of Fu and Sufu depended on Ci (Fig. 2I). These observations suggest that the nuclear Ci-Sufu-Fu complex may contribute to Ci^A^ function.

If mature Fu is part of the Ci^A^ complex, preventing Fu from entering the nucleus should impair Ci^A^ function. Therefore, we generated Cl8 cells stably expressing a myristoylated form of Fu (Fu^Myr^) to trap exogenously expressed Fu in the cytoplasm (fig. S1I). After depletion of endogenous Fu, RNAi-insensitive Fu^Myr^ was still activated by Hh, underwent maturation, and phosphorylated Ci, albeit with reduced efficiency compared with Fu^WT^; however, phosphorylated Ci was trapped in the cytoplasm and failed to activate *ptc* (fig. S3, E and F), suggesting that Ci phosphorylation by Fu precedes nuclear transport. In addition, Fu^Myr^ failed to rescue the *fu^M1^* mutant phenotypes (fig. S3, G and H). Hence, nuclear Ci^A^ consists of a Ci-Sufu-Fu complex in an active conformation rather than “free” Ci.

### Fu maturation is promoted by phosphorylation-dependent SUMOylation and SUMO-SIM interaction

**Fig. 3.**
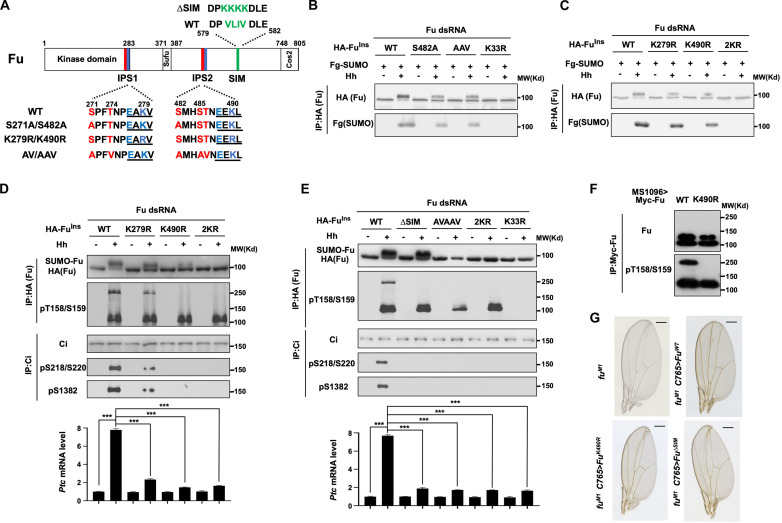
Phosphorylation-dependent SUMOylation promotes Fu maturation and Ci activation. (**A**) Schematic drawing of Fu with its SIM and two inverted phosphorylation-dependent SUMOylation motifs (IPS1 and IPS2) indicated. (**B**) WB analyses of Fu SUMOylation in Fu-depleted Cl8 cells stably expressing RNAi-insensitive WT, S482A, AAV, or K33R version of HA-Fu, transiently transfected with Flag-SUMO and treated with Hh-conditioned or control medium. (**C**) WB analysis of Fu SUMOylation in Fu-depleted Cl8 cells stably expressing RNAi-insensitive WT, K279R, K490R, and 2KR version of HA-Fu, transiently transfected with Flag-SUMO and treated with Hh-conditioned or control medium. (**D** and **E**) WB analysis of Ci phosphorylation, Fu phosphorylation and maturation (top) and qRT-PCR analysis of *ptc* mRNA (bottom) in Fu-depleted Cl8 cells stably expressing the indicated RNAi-insensitive Fu constructs and treated with control or Hh-conditioned medium. Data are means ± SD from two independent experiments. ***P < 0.001 (*t* test). (**F**) WB analysis of Fu maturation in wing discs expressing Myc-Fu-WT or Myc-Fu-K490R using the *MS1096* Gal4 driver. A total of 150 discs were used for each genotype. (**G**) Adult wings of *fu^M1^* mutants without or with transgenic expression of the indicated *fu* transgenes. Scale bars, 500 μm.

Our previous study revealed that the SUMO pathway regulates Hh signaling by promoting Smo cell surface accumulation (*43*). Therefore, we were surprised to find that the activity of a constitutively active and Smo-independent Fu (CC-Fu^EE^) diminished in wing discs that coexpressed dsRNA targeting the SUMO-conjugating enzyme Ubc9 (fig. S4A). Blocking SUMOylation either by Ubc9 RNAi or overexpression of the deSUMOylation enzyme Ulp1 abolished Fu^EE^-mediated activation of Ci^-PKA^ in S2 cells (fig. S4, B and C), indicating that the SUMO pathway may also regulate Fu. Hh induced a mobility shift of Fu to a >100-*K*_d_ position, which was abolished by treatment with a catalytically active yeast Ulp1 (fig. S4D), suggesting that the >100-*K*_d_ Fu species were SUMO-conjugated Fu (SUMO-Fu). Under appropriate electrophoresis conditions, two SUMOylated Fu species were resolved, likely corresponding to the addition of one or two SUMO moieties (fig. S4E). The conjugation of Flag-SUMO to Fu was abolished by the K33R mutation and greatly reduced by the S482A or AAV mutation in Cl8 cells stimulated with Hh (Fig. 3B), suggesting that Hh-induced phosphorylation of FuR promotes SUMOylation of Fu. Of note, the SUMO epitope was not detected in the SDS-resistant Fu dimers but was detected in the Fu monomers derived from the dimers after extensive incubation in 2× SDS loading buffer (fig. S3B), suggesting that the mature Fu was SUMO conjugated, but the SUMO epitope was not exposed.

SUMOylation sites have consensus motifs: ψKxE/D and E/DxKψ (inverted site), where ψ is a large hydrophobic amino acid and x is any amino acid (*53*). SUMOylation of several proteins was promoted by phosphorylation in the so-called phosphorylation dependent SUMOylation motifs (PDSM): ψKxE/DxxpS/T (*53*–*55*). Several putative SUMOylation sites were noticeable in Fu, including K279 (_271_SPFTNPEAKV_280_) and K490 (_482_SMHSTNEEKL_491_), both of which are adjacent to Fu-primed CK1 sites with a space of four amino acids (Fig. 3A). We speculated that Fu/CK1-mediated phosphorylation may convert these SUMOylation sites into inverted PDSM (IPS), which we name IPS1 and IPS2 (Fig. 3A). Both in vitro and in vivo assays showed that phosphorylation of IPS1/2 by Fu/CK1 promoted their SUMOylation at K279 and K490, respectively (fig. S4, F and G). Either K279R or K490R mutation reduced whereas the combined mutation (2KR) abolished Hh-induced SUMOylation of Fu (Fig. 3C). Furthermore, both 2KR and AVAAV abolished Hh-induced Fu maturation and Ci activation (Fig. 3, D and E). K490R abolished whereas K279R only reduced Fu maturation and Ci activation, suggesting that IPS2 is more critical than IPS1 for Fu function. K490R abolished Fu maturation of exogenously expressed Fu in wing imaginal discs (Fig. 3F). In addition, *C765>Fu^K490R^* failed to rescue the *fu^M1^* mutant phenotypes (Fig. 3G and fig. S4H). Hence, Hh-induced and phosphorylation-dependent SUMOylation of Fu regulatory domain promotes Fu maturation and Ci activation.

SUMOylation often mediates protein-protein interaction by binding to SIM, which consists of a stretch of hydrophobic amino acids adjacent to acidic residues (*56*). FuR contains an SIM motif (DP_579_VLIV_582_ DLE) located C-terminally to IPS2 (Fig. 3A). Mutating SIM (∆SIM: VLIV to KKKK) did not affect Fu^∆SIM^ phosphorylation or SUMOylation but abolished Fu^∆SIM^ maturation and Ci activation in Fu-depleted Cl8 cells treated with Hh (Fig. 3E). Furthermore, *C765>Fu^∆SIM^* failed to rescue the *fu* mutant phenotypes (Fig. 3F and fig. S4G). These results suggest that Fu maturation and Ci activation may rely on SUMO-SIM interaction.

### SUMO-SIM interaction promotes Fu self-association to form biomolecular condensates

**Fig. 4.**
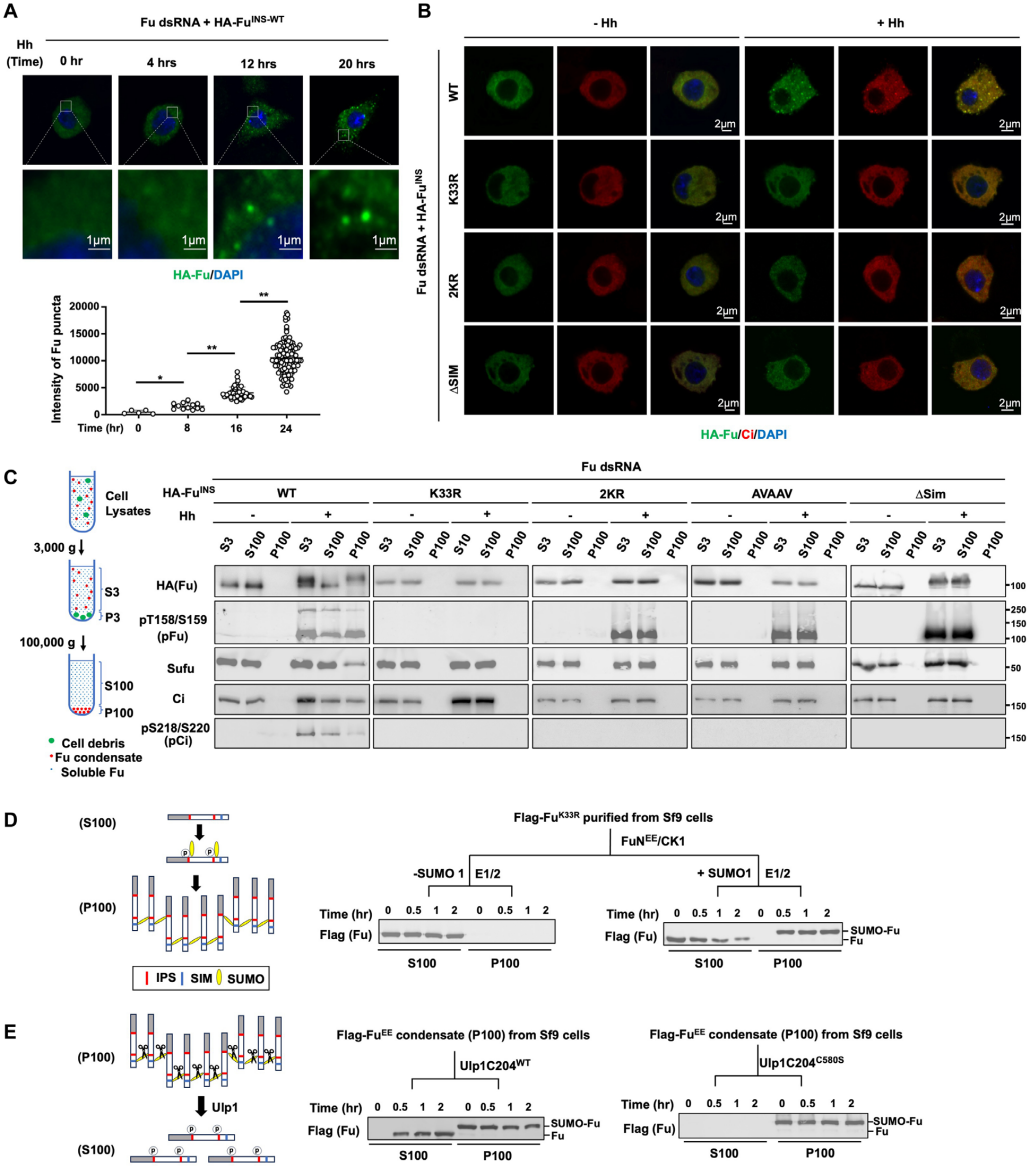
Hh induces Fu kinase condensation mediated by SUMO-SIM interaction. (**A**) Top, immunostaining of HA-Fu in Fu-depleted Cl8 cells stably expressing RNAi-insensitive HA-Fu and treated with Hh-conditioned medium for the indicated time. Bottom, quantification of HA-Fu condensates in (A) from *n* = 10 cells for each time point. Data are means ± SD. *P < 0.05, **P < 0.01 (*t* test). (**B**) Immunostaining of Ci and HA-Fu proteins in Fu-depleted Cl8 cells stably expressing the indicated RNAi-insensitive HA-Fu constructs and treated with Hh-conditioned or control medium for 16 hours. (**C**) Left, schematic drawing of the sedimentation assay. Right, WB analyses of Fu, Ci, Sufu, phosphorylated Fu, and Ci in S3, S100, and P100 fractions from Fu-depleted Cl8 cells stably expressing the indicated RNAi-insensitive HA-Fu constructs and treated with Hh-conditioned or control medium. (**D**) Left, schematic drawing of in vitro condensation of Fu driven by phosphorylation and SUMOylation. Right, Flag-tagged kinase dead Fu (Fg-Fu^K33R^) purified from Sf9 cells was subjected to in vitro kinase assay by FuN^EE^ and CK1, followed by in vitro SUMOylation assay in the presence or absence of SUMO1 for the indicated periods of time. The distribution of Fg-Fu^K33R^ in S100 and P100 at the indicated time points was analyzed by immunoblot after centrifugation. (**E**) Left, schematic drawing of in vitro deSUMOylation and de-condensation of Fu. Right, Flag-Fu^EE^ condensates (p100) isolated from Sf9 cells were resuspended and deSUMOylated with WT or enzymatic dead (C580S) Ulp1C204 fused to GST for the indicated periods of time. The distribution of total Fu and phosphorylated Fu in condensates (P100) and supernatant (S100) at different time points was analyzed by immunoblot after re-centrifugation.

SUMOylated proteins that also contain SIM can self-associate to form biomolecular condensation as has been shown for promyelocytic leukemia nuclear bodies (*57*). We speculate that Hh-induced SUMOylation and subsequent SUMO-SIM interaction may promote Fu maturation by inducing Fu self-association to form condensates. To test this hypothesis, we treated Fu-depleted Cl8 cells stably expressing hemagglutinin (HA)–Fu^WT^ at near endogenous level (fig. S1I) with Hh-conditioned medium for increasing amount of time. As shown in Fig. 4A, Hh induced the formation of HA-Fu puncta in the cytoplasm and the intensity and size of the puncta increased over time. Hh also induced the formation of Ci puncta that colocalized with the HA-Fu puncta (Fig. 4B). Mutations blocking Fu kinase activity (K33R), SUMOylation (2KR), or SUMO-SIM interaction (∆SIM) all diminished Hh-induced formation of Fu/Ci puncta (Fig. 4B). Ci was recruited to Fu condensates through Sufu because Ci did not colocalize with Fu∆Sufu puncta (fig. S5A). In addition, Sufu was also recruited to the HA-Fu puncta together with Ci (fig. S5B).

Protein condensates can be separated from soluble proteins by sedimentation due to their high density (*58*, *59*). Therefore, we carried out density fractionation of Fu-depleted Cl8 cells stably expressing HA-Fu^WT^, Fu^K33R^, Fu^2KR^, Fu^AVAAV^, or Fu^∆SIM^ before and after Hh treatment (Fig. 4C). In the absence of Hh, HA-Fu^WT^ could only be detected in the soluble fraction (S100); however, after Hh treatment, HA-Fu^WT^ was detected in both the soluble fraction and condensates (P100) (Fig. 4C). By contrast, phosphorylation-, SUMOylation-, or SIM-deficient Fu variants were not detected in the condensates (Fig. 4C), confirming that phosphorylation-mediated SUMOylation of Fu is essential for Fu condensation. Immunoblot analysis showed that both Ci and Sufu were also present in the P100 fraction from cells expressing HA-Fu^WT^ (Fig. 4C), confirming that Ci and Sufu were recruited into Fu condensates in response to Hh.

To investigate the biophysical properties of Fu condensates, we applied various chemicals to dissolve Fu in the P100 fraction derived from Hh-treated Cl8 cells. As shown in fig. S5C, Fu condensates were resistant to 1% Triton X-100 or 4 M urea, suggesting that Fu condensation was not due to its membrane association or forming prion-like protein aggregates. Fu condensates also sustained the treatment of 1.0 M NaCl, 5% 1, 6-hexanediol, or 1% SDS at 4°C, conditions that normally disrupt liquid-liquid phase separation (LLPS) (*60*), suggesting that Fu condensates were more rigid and gel-like condensates (*61*). Consistent with this, fluorescence recovery after photobleaching (FRAP) analysis revealed that Hh-induced mEGFP-Fu condensates in Cl8 cells exhibited slow dynamics with *t*_1/2_ > 20 min (fig. S5D).

To further demonstrate that phosphorylation-dependent SUMOylation and SUMO-SIM interaction drive Fu condensation, we carried out in vitro reconstitution experiments in which Flag-Fu^K33R^ purified from Sf9 cells was subjected to in vitro phosphorylation and SUMOylation, followed by high-speed ultracentrifugation. As shown in Fig. 4D, increasing amounts of Fu^K33R^ were converted from soluble proteins to condensates over time after in vitro SUMOylation whereas unsumoylated Fu^K33R^ stayed in the soluble fraction. Mutating the SUMO sites (2KR) prevented Fu^K33R/2KR^ from being converted into condensates after in vitro phosphorylation and SUMOylation (fig. S5E). On the contrary, Sf9-derived Flag-Fu^EE^ condensates were gradually converted to soluble forms after deSUMOylation by Ulp1 (Fig. 4E), suggesting that Fu condensation is a reversible process driven by SUMOylation and deSUMOylation.

### Fu condensation drives Fu maturation and Ci activation

**Fig. 5.**
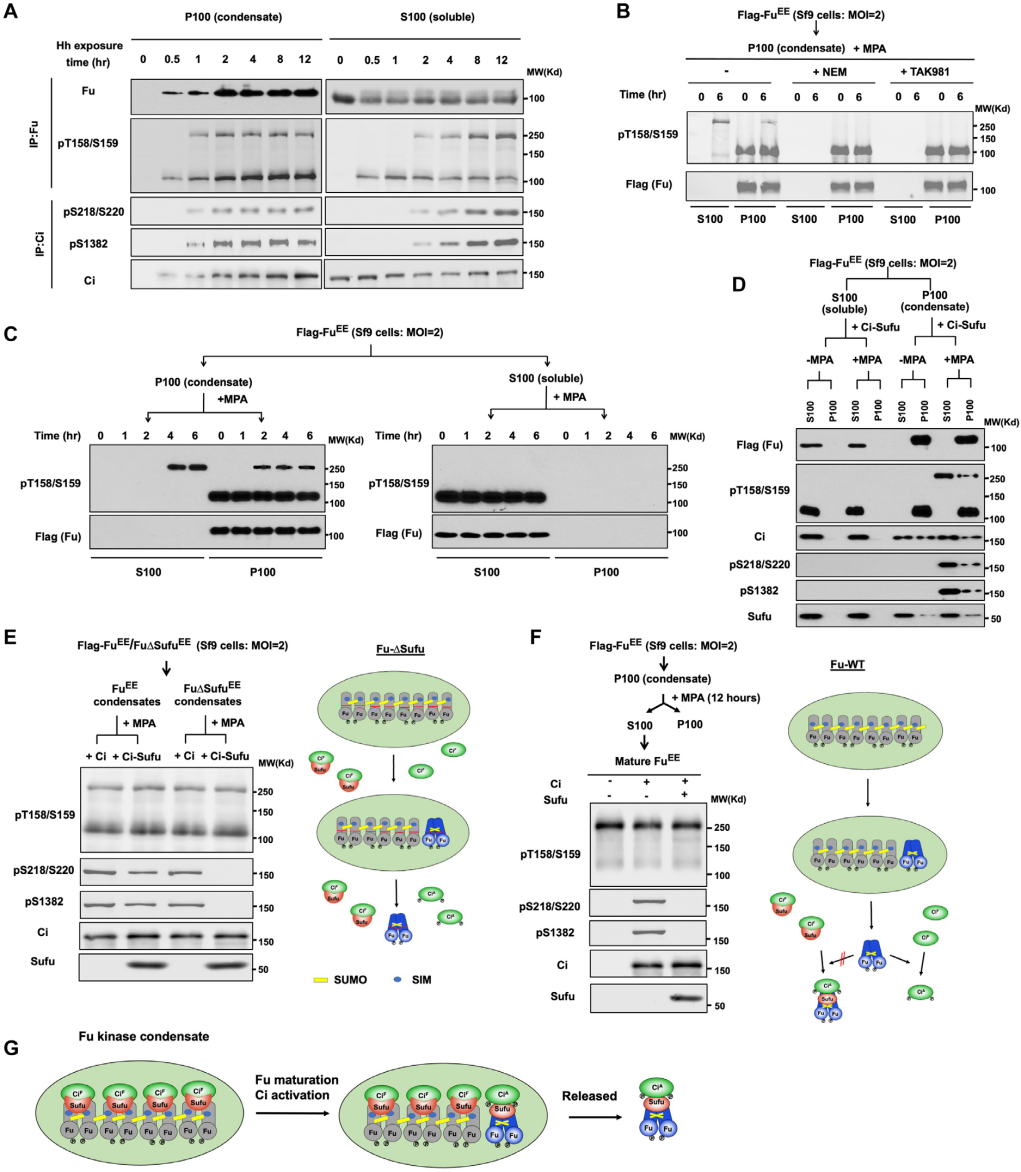
Fu kinase condensation drives Fu maturation and Ci activation. (**A**) WB analysis of endogenous Fu maturation and Ci phosphorylation in S100 and P100 fractions from Cl8 cells treated with Hh-conditioned medium for the indicated periods of time. (**B**) In vitro maturation assay of Fu in the absence or presence of NEM (deSUMOylation inhibitor) or TAK981(SUMOylation inhibitor). (**C**) In vitro maturation assay of Fu. Flag-Fu^EE^ proteins expressed in Sf9 cells at high MOI were separated into S100 and P100 fractions. Purified (S100) or resuspended (P100) Flag-Fu^EE^ proteins were incubated with MPA (Sf9 cell lysate, ATP, and SUMO1) for the indicated periods of time. The distribution of Fu in condensates (P100) and supernatant (S100) at different time points after incubation was analyzed by immunoblot after re-centrifugation. (**D**) In vitro Fu maturation and Ci phosphorylation. Flag-Fu^EE^ proteins expressed in Sf9 cells at high MOI were separated into S100 and P100 fractions and then incubated with or without MPA in the presence of Sufu-Ci complexes purified from Sf9 cells for 6 hours, followed centrifugation into S100 and P100 fractions and immunoblot analysis with the indicated antibodies. (**E**) Left, Flag-tagged Fu^EE^ or Fu∆Sufu^EE^ condensates (P100) derived from Sf9 cells were resuspended and incubated with MPA in the presence of free Ci or Sufu-Ci purified from Sf9 cells, followed by immunoblot analysis with the indicated antibodies. Right, Fu∆Sufu can undergo maturation but fail to phosphorylate Sufu-bound Ci although it can phosphorylate free Ci. (**F**) Left, Flag-Fu^EE^ was purified from S100 after in vitro maturation experiments and then incubated with either free Ci or Sufu-Ci, followed by immunoblot analysis with the indicated antibodies. Right, the released mature Fu is unable to phosphorylate Sufu-bound Ci but can still phosphorylate free Ci. (**G**) Model of Ci phosphorylation driven by Fu maturation in Fu condensates.

Mature Fu and phosphorylated Ci were detected in both the soluble and condensate fractions after Hh stimulation (Fig. 4D). To determine where Fu maturation and Ci phosphorylation occur, we carried out time course experiments in which Cl8 cells were treated with Hh for increasing amount of time and cell extracts were fractionated into S100 and P100 followed by immunoblot analysis. As shown in Fig. 5A, Fu condensates were detected after Hh treatment for 0.5 hours, followed by the detection of mature Fu and phosphorylated Ci initially in condensates (~1 hour after Hh treatment) and then in supernatants (~2 hours after Hh treatment). Furthermore, the amount of mature Fu and phosphorylated Ci gradually increased in the soluble fraction but remained relatively constant in condensates at later time points (Fig. 5A). These observations suggest that mature Fu and phosphorylated Ci were generated in condensates and then released into the soluble fraction.

To demonstrate that Fu condensation drives Fu maturation, we carried out in vitro maturation experiments. We expressed a constitutively active Fu (Flag-Fu^EE^) in Sf9 cells infected with high MOI (multiplicity of infection). Under high MOI (MOI = 2), Fu maturation was largely suppressed in Sf9 cells (fig. S5F), likely due to the suppression of endogenous protein synthesis that could limit the amount of host factor(s) required for Fu maturation. However, Fu SUMOylation and condensation still occurred under these conditions (Fig. 5C). Soluble Fu^EE^ and Fu^EE^ condensates were incubated with Sf9 cell lysates in the presence of adenosine triphosphate (ATP) and SUMO1 (hereafter referred to as MPA for maturation promoting activity) for increasing amount of time, followed by ultracentrifugation. Fu^EE^ in the condensates but not in the soluble fraction was gradually converted into the mature form, which was then released into the soluble fractions (Fig. 5C). Addition of either SUMOylation inhibitor TAK981 or deSUMOylation inhibitor NEM prevented Fu^EE^ maturation (Fig. 5B), suggesting that Fu maturation depends on a dynamic process involving SUMOylation and deSUMOylation.

We next determined whether Fu maturation drives Ci phosphorylation. In the aforementioned in vitro maturation experiments, we introduced Ci-Sufu complexes purified from Sf9 cells as substrates to determine whether Ci could be activated as indicated by S218/S220/S1382 phosphorylation. As shown in Fig. 5D, Ci was activated only under conditions that promoted Fu maturation (P100/+MPA). By contrast, Fu∆Sufu^EE^ maturation failed to phosphorylate Sufu-bound Ci, although it could still phosphorylate unbound Ci (Fig. 5E). Furthermore, the preexisting mature Fu^EE^ purified from the in vitro maturation experiments failed to phosphorylate Sufu-bound Ci (Fig. 5F). Hence, Fu maturation and Ci phosphorylation are both physically and temporarily coupled, suggesting that they occur in the same complex (Fig. 5G).

### Ulk3 activates Gli2 via a conserved mechanism in mammalian cells

**Fig. 6.**
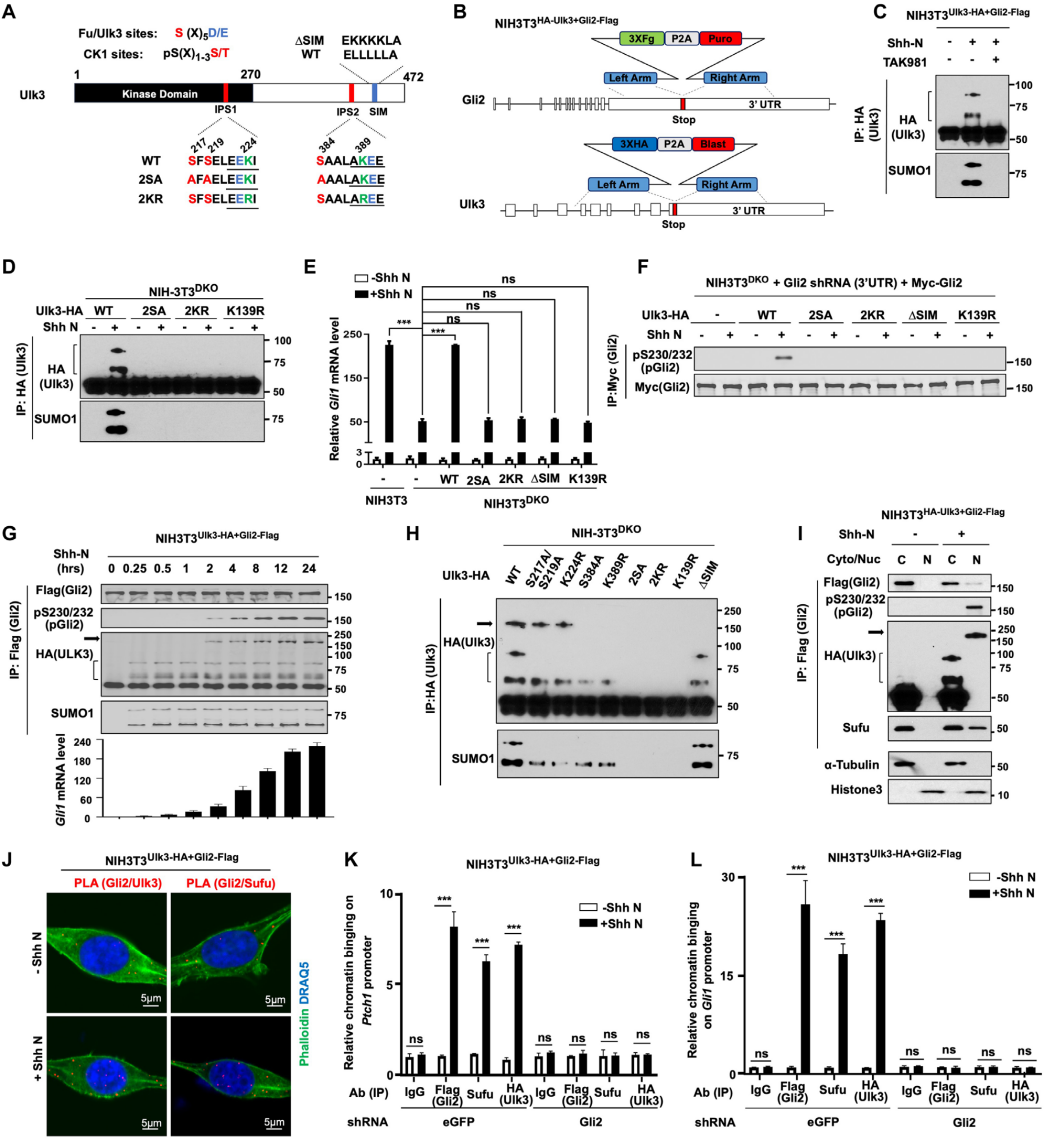
Ulk3 undergoes phosphorylation- and SUMOylation-dependent conformation maturation to activate Gli2. (**A**) Schematic drawing of Ulk3 with WT and mutant sequences of IPS1, IPS2, and SIM indicated. (**B**) Diagram of tagging Gli2 and Ulk3 with 3× Flag and 3× HA on C-terminal ends by CRISPR-Cas9 system in the NIH3T3^Ulk3-HA+Flag-Gli2^ cell line. (**C**) WB analysis of Ulk3 SUMOylation in NIH3T3^Ulk3-HA+Flag-Gli2^ cells untreated or treated with Shh and without or with TAK981. Bracket indicates SUMOylated Ulk3. (**D**) WB analysis of SUMOylation of exogenously expressed WT and mutant Ulk3-HA in NIH3T3^DKO^ cells. (**E**) qRT-PCR analysis of *Gli1* mRNA expression in control or DKO NIH3T3 cells expressing the indicated Ulk3 constructs. (**F**) WB analysis of Gli2 phosphorylation in NIH3T3^DKO^ cells with endogenous Gli2 depleted but expressing Myc-Gli2 and the indicated Ulk3 constructs. (**G**) WB analysis of Gli2 phosphorylation and Ulk3 SUMOylation and maturation and qRT-PCR analysis of *Gli1* mRNA expression in NIH3T3^Ulk3-HA+Flag-Gli2^ cells treated with Shh for increasing amounts of time. Arrow indicates mature Ulk3, whereas bracket indicates SUMOylated Ulk3. (**H**) WB analysis of Ulk3 SUMOylation and maturation in NIH3T3^DKO^ cells expressing the indicated Ulk3 constructs and treated with Shh. (**I**) WB analysis of cytoplasmic (C) and nuclear (N) Gli2 complexes isolated by IP from NIH3T3^Ulk3-HA+Flag-Gli2^ cells treated with or without Shh. (**J**) PLA of Gli2 and Ulk3 or Gli2 and Sufu in NIH3T3^Ulk3-HA+Flag-Gli2^ cells treated with or without Shh. (**K** and **L**) ChIP qPCR analysis of chromatin-associated Gli2, Sufu, and Ulk3 on *Ptch1* (K) and *Gli1* (L) promoters in control or Gli2-depleted NIH3T3^Ulk3-HA+Flag-Gli2^ cells treated with or without Shh. Data are means ± SD from three (E) or two [(K) and (L)] independent experiments. ***P < 0.001 (*t* test).

Our previous study revealed that the Fu/Ulk family members Ulk3 and Stk36 act in parallel to promote Gli2 phosphorylation at S230/S232 and S1528 located within the Sufu-binding domains, and phosphorylation at these sites promotes Gli2 activation (*29*–*31*). Except for a conserved kinase domain, the Fu/Ulk family kinases share little if any sequence similarity in their regulatory domains. We found that Ulk3 also contains two putative IPSs (IPS1 and IPS2) and an SIM motif downstream of IPS2 (Fig. 6A). In vitro experiments revealed that Ulk3/CK1 phosphorylates IPS1/2 to promote their SUMOylation (fig. S6, A to E). Using a double knockin NIH-3 T3 cell line (NIH3T3^Ulk3-HA+Gli2-Flag^) with 3× HA inserted into the Ulk3 locus and 3× Flag inserted into the Gli2 locus generated by CRISPR-Cas9 gene editing (Fig. 6B), we found that Shh induced SUMO conjugation of endogenously expressed Ulk3-HA (Fig. 6C). Shh induced SUMOylation of exogenously expressed Ulk3-HA in Ulk3/Stk36 double knockout cells (NIH3T3^DKO^) ^33^ at K224 and K389 dependent on phosphorylation at adjacent Ulk3/CK1 sites (Fig. 6D and fig. S6, D to F). Phosphorylation-dependent SUMOylation at IPS1/2 is essential for Ulk3-HA to promote Gli2 phosphorylation and *Gli1* expression in NIH3T3^DKO^ cells treated with Shh (Fig. 6, E and F, and fig. S6, G and H), with IPS2 playing a more critical role than IPS1. Mutating the SIM motif (∆SIM) also abolished the ability of Ulk3-HA to phosphorylate and activate Gli2 (Fig. 6, E and F).

When overexpressed in human embryonic kidney (HEK) 293 cells, Ulk3^WT^ but not the kinase dead Ulk3^K139R^ phosphorylated coexpressed Myc-Gli2, suggesting that overexpression could promote Ulk3 kinase activation (fig. S6I). SUMOylation-deficient Ulk3 (Ulk3^2SA^ or Ulk3^2KR^) could still phosphorylate free Gli2; however, only Ulk3^WT^ but not Ulk3^2SA^ or Ulk3^2KR^ could phosphorylate Sufu-bound Gli2 (fig. S6, I and J), suggesting that SUMOylation plays an allosteric role by unmasking Gli2 in the Sufu-Gli2 complex.

When NIH3T3^Ulk3-HA+Gli2-Flag^ cells were treated with Shh for increasing amount of time, an SDS-resistant Ulk3 oligomer (hereafter called mature Ulk3) emerged after Shh treatment for 2 hours, which temporarily coincided with Gli2 phosphorylation and activation (Fig. 6G). Ulk3 maturation, Gli2 phosphorylation, and activation increased progressively overtime and plateaued at 24 hours after ligand exposure (Fig. 6G). Overexpression of Ulk3 in NIH3T3^DKO^ cells also promoted Ulk3 maturation, which depended on its kinase activity, SUMOylation, and SIM (Fig. 6H). While mutating IPS1 (S217A/S219A or K224R) partially blocked Ulk3 maturation, mutating IPS2 (S384A or K389R) completely blocked Ulk3 maturation (Fig. 6H), which is in line with the pathway activities exhibited by these variants (Fig. 6, E and F, and fig. S6, G and H), suggesting that IPS2 is more essential than IPS1 for Ulk3 maturation and Gli activation. Of note, the SUMO epitope was not detected in the SDS-resistant Ulk3 oligomers (Fig. 6H) but was detected in the Ulk3 monomers derived from the oligomers after prolonged incubation in 2× SDS loading buffer (fig. S6K), suggesting that the mature Ulk3 was SUMO-conjugated, but the SUMO epitope was masked.

Consistent with Ulk3 maturation allosterically activating Gli2, mature Ulk3 formed a complex with phosphorylated Gli2 and Sufu in the nucleus of NIH3T3^Ulk3-HA/Gli2-Flag^ cells treated with Shh (Fig. 6I). PLA revealed that Gli2 formed a complex with Ulk3 and Sufu in the nucleus of intact cells (Fig. 6J). ChIP experiments showed that Gli2, Ulk3, and Sufu co-occupied both *Ptch1* and *Gli1* promoters and that the chromatin association of Ulk3 and Sufu depended on Gli2 (Fig. 6, K and L). Together, these results suggest that (i) Shh-induced SUMOylation promotes Ulk3 conformation maturation that allosterically activates Gli2, and (ii) Gli2^A^ exists in a complex with Sufu and mature Ulk3.

### Ulk3 forms a condensate at ciliary tip to activate Gli2

**Fig. 7.**
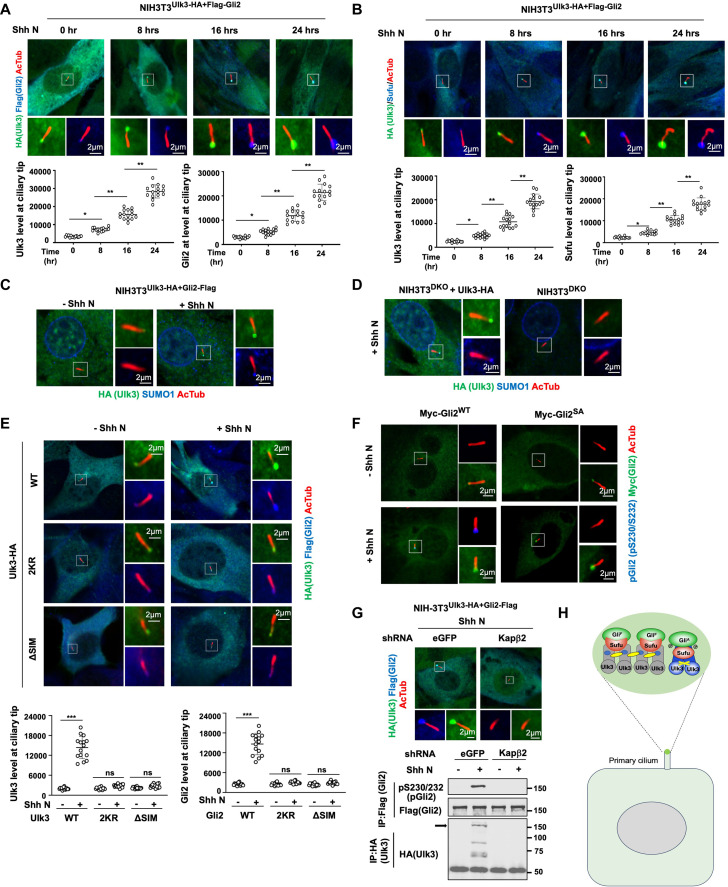
Shh-induced Ulk3 condensation promotes Gli2 activation at ciliary tip. (**A** and **B**) Representative images of immunostaining (top) and quantification (bottom; *n* = 15 cells) of ciliary-localized Ulk3-HA and Flag-Gli2 (A) or Sufu (B) in NIH3T3 cells expressing endogenously tagged Ulk3 and Gli2 and treated with Shh for the indicated time. Data are means ± SD. *P < 0.05, **P < 0.01 (*t* test). Acetylated tubulin (AcTub) staining marks the primary cilia. (**C**) Representative images of immunostaining of ciliary-localized Ulk3-HA and SUMO1 in NIH3T3^Ulk3-HA+Flag-Gli2^ cells treated with or without Shh. (**D**) Representative images of immunostaining of ciliary-localized Ulk3-HA and SUMO1 in NIH3T3^DKO^ cells infected with or without lentiviral Ulk3-HA and treated with Shh. (**E**) Representative images of immunostaining (left) and quantification (right, *n* = 15 cells) of ciliary-localized Ulk3 and Gli2 in Ulk3/Stk36 DKO and Gli2 depleted NIH3T3 cells expressing Flag-Gli2 and the indicated Ulk3-HA constructs and treated with or without Shh. Data are means ± SD. ***P < 0.001 (*t* test). (**F**) Immunostaining to examine the ciliary Gli2 phosphorylation (pGli2) in NIH3T3 cells transfected with Myc-Gli2^WT^ or Myc-Gli2^SA^ and Flag-Sufu and treated with or without Shh. (**G**) NIH3T3 cells expressing Ulk3-HA and Flag-Gli2 at endogenous loci were treated with control (eGFP) or Kapβ2 shRNA in the presence or absence of Shh, followed by immunostaining to examine Ulk3/Gli2 ciliary localization (top) or IP and WB analysis to examine Ulk3 maturation and Gli2 phosphorylation (bottom). (**H**) Schematic drawing of Ulk3 condensation at ciliary tip that drives Ulk3 maturation and Gli2 activation.

Primary cilia play an essential role in vertebrate Hh signal transduction (*32*). In response to Hh, both Gli2 and Sufu accumulate at ciliary tip and hindering Gli2 ciliary localization blocks its phosphorylation and activation (*29*, *62*–*64*). We found that Ulk3-HA accumulated at ciliary tips and colocalized with Gli2-Flag and Sufu in NIH3T3^Ulk3-HA+Gli2-Flag^ cells treated with Shh and that the levels of Ulk3-HA, Gli2-Flag, and Sufu at ciliary tips increased progressively over time (Fig. 7, A and B). SUMO-1 signal was also accumulated at ciliary tips in response to Shh, which correlated with Ulk3 accumulation (Fig. 7, C and D). Blocking phosphorylation-induced SUMOylation of Ulk3 (K139R, 2SA, and 2KR) or mutating SIM (∆SIM) abrogated Shh-induced ciliary tip accumulation of Ulk3-HA, Gli2-Flag, and Sufu in NIH3T3^DKO^ cells (Fig. 7E and fig. S6, L and M), suggesting that Shh-induced SUMOylation of Ulk3 and subsequent SUMO-SIM interaction promoted the formation of Ulk3 condensates that also recruit Gli2 and Sufu at ciliary tips. Supporting Gli2 being activated at ciliary tips, ciliary tip–localized Myc-Gli2^WT^ but not Myc-Gli2^S230A/S232A^ (Gli2^SA^) was immunostained by the pS230/232 (pGli2) antibody (Fig. 7F). Furthermore, blocking ciliary tip accumulation of Ulk3/Gli2 in Kapβ2 knocked down cells (*62*) inhibited Ulk3 maturation and Gli2 phosphorylation (Fig. 7G). These results suggest that SUMO-conjugated Ulk3 formed condensates at ciliary tips to promote Ulk3 maturation and Gli2 activation (Fig. 7H).

Ulk3 forms condensates at ciliary tips likely due to (i) its local activation by ciliary Smo in response to Shh and (ii) relatively high local concentrations of Hh pathway components. Overexpression of Ulk3-HA^WT^, which promoted Ulk3 kinase activation (fig. S6I), together with Myc-Gli2 and Flag-Sufu in HEK293T cells led to the formation of cytoplasmic puncta containing Ulk3-HA, Myc-Gli2, and Flag-Sufu (fig. S7A). By contrast, overexpression of the kinase dead (Ulk3-HA^K139R^), phosphorylation-, SUMO-, or SIM-deficient Ulk3 variants failed to form cytoplasmic puncta (fig. S7A). Cytoplasmic Ulk3 condensates in HEK293T cells were also positively labeled with the SUMO1 antibody (fig. S7B). In addition, pGli2 signals were detected in Ulk3 condensates containing Myc-Gli2^WT^ but not in those containing Myc-Gli2^SA^ (fig. S7C), consistent with Gli2 being phosphorylated and activated in the Ulk3 condensates. In the sedimentation assay, Ulk3-HA^WT^ was detected in both soluble fraction (S100) and condensates (P100), and SUMO-conjugated Ulk3 (SUMO-Ulk3) was enriched in the condensates (fig. S7, D and E), whereas Ulk^K139R^, Ulk3^2KR^, and Ulk3^∆SIM^ failed to form condensates (fig. S7E). In vitro maturation experiments revealed that Ulk3 in the condensates but not in the soluble fraction produced mature Ulk3 and phosphorylated Gli2 (fig. S7F), suggesting that Ulk3 condensation drives Ulk3 maturation and Gli2 activation (Fig. 7H).

## DISCUSSION

**Fig. 8.**
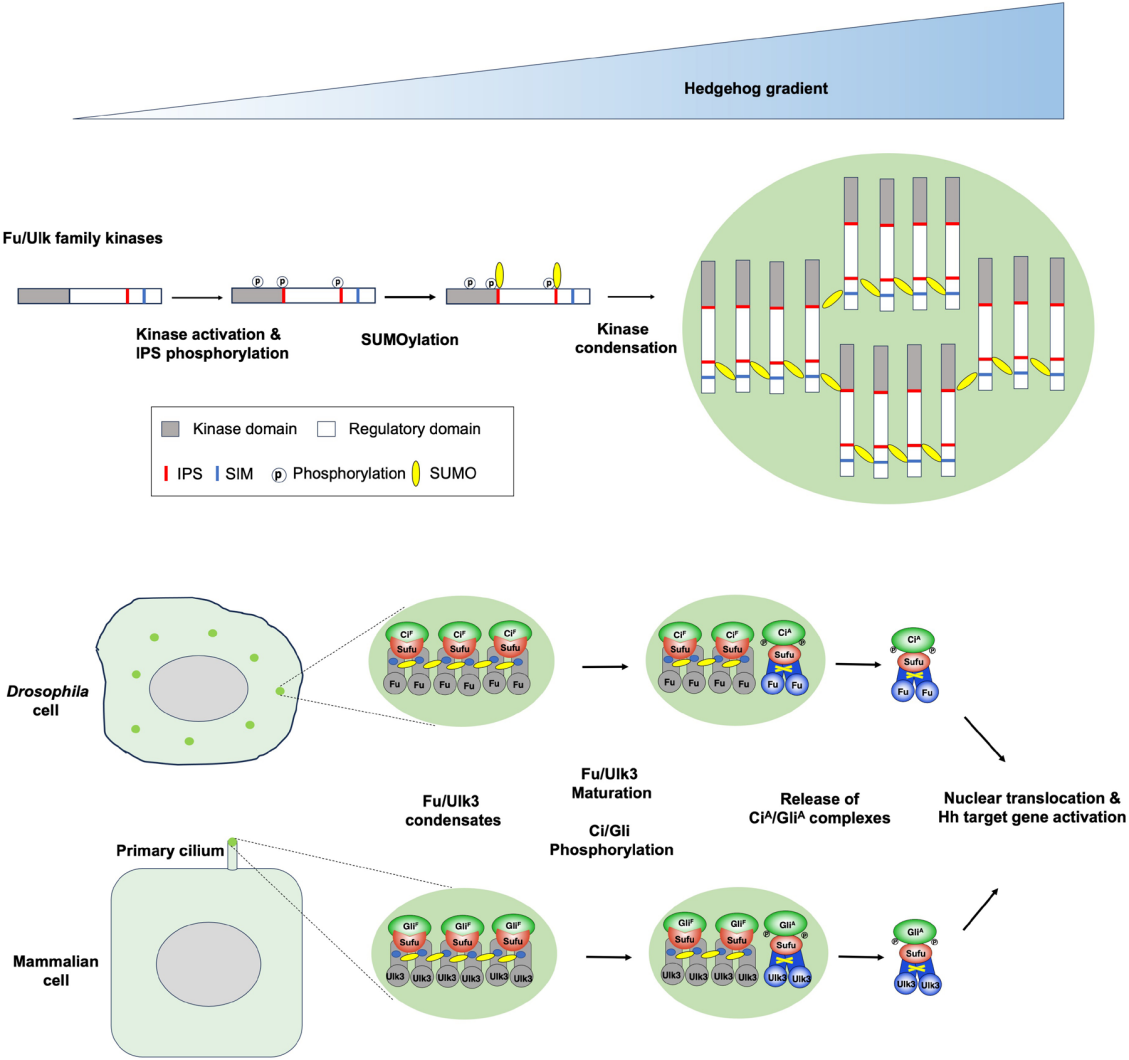
Hh morphogen induces Fu/Ulk3 condensation via phosphorylation-dependent sumoylation to allosterically activate Ci/Gli. (**Top**) Fu/Ulk3 kinase activation requires low Hh concentration and short ligand exposure time, whereas SUMO-SIM–driven condensation, Fu/Ulk3 conformational maturation, and Ci/Gli phosphorylation and activation require higher Hh concentration and longer ligand exposure time. For simplicity, Sufu and Ci are not drawn in the kinase condensate. (**Bottom**) Hh induces condensation of Fu and Ulk3 in the cytoplasm and primary cilium, respectively. Within the condensates, Fu/Ulk3 undergo a conformation maturation to allosterically activate Ci/Fu. The Ci^A^/Gli^A^ complex containing mature Fu/Ulk3 and Sufu enter the nucleus and activate Hh target genes. See text for details.

How Hh converts the latent transcription factor Ci/Gli into Ci^A^/Gli^A^ has remained a major unsolved question. Here, we show that signal-induced formation of kinase condensates to allosterically activate pathway transcription factors represents a unified mechanism for Hh signal transduction from *Drosophila* to mammals despite their differential requirement for primary cilia. We provide evidence that Hh induces Fu/Ulk3 conformational change and Ci/Gli activation through phosphorylation-regulated SUMOylation and SUMO-SIM–mediated phase separation of the kinases (Fig. 8).

In a conventional kinase-mediated signal transduction cascade, activated kinases transiently bind and phosphorylate their substrates. This “hit and run” or catalytic mechanism allows one kinase to phosphorylate many substrates to rapidly amplify the signal. Here, we show that Fu/Ulk3 does not simply act catalytically but rather forms a stochiometric complex with Ci/Gli to allosterically activate Ci/Gli. By using a stochiometric mechanism for Ci/Gli activation through conformational change, Fu/Ulk3-mediated Hh signal transduction results in a linear increase of signal outputs that is proportional to signal inputs, allowing cells to be able to respond to a broad range of Hh morphogen inputs. In addition, the slow process of Fu/Ulk3 maturation compared with its kinase activation may allow Ci^A^/Gli^A^ activity to be built up gradually and in proportion to Hh exposure time, which may explain why cellular responses are sensitive to the duration of Hh exposure and why high-threshold responses require not only higher Hh dose but also longer Hh exposure time than low-threshold responses (*65*, *66*). How Hh induces Fu/Ulk3 kinase activation remains to be determined. In *Drosophila*, Fu is activated in Cos2-Fu complexes bound to activated Smo (*26*–*28*). Therefore, activated Fu from Cos2-Fu complexes may trans-phosphorylate Fu in Fu-Sufu-Ci complexes or there could be intermediate Cos2-Fu-Sufu-Ci complexes.

Biomolecular condensates participate in many cellular processes, and their formation is often constitutive or regulated by stress signals (*39*, *40*). Whether the formation of intracellular condensates can be regulated by extracellular signals such as morphogens has been underexplored. Here, we report that Hh induces the formation of kinase condensates in a dose- and time-dependent manner and reveal a novel mechanism that links kinase activation to phase separation (Fig. 8). We found that both Fu and Ulk3 contain two inverted phosphorylation-dependent SUMOylation motifs (IPS1/2). In response to Hh, Fu/Ulk3 and CK1 phosphorylate these newly identified motifs, leading to their SUMOylation. Subsequent SUMO-SIM–mediated interaction promotes Fu/Ulk3 polymerization to form biomolecular condensates (Fig. 8). Because phosphorylation-induced SUMOylation has been observed in many regulatory proteins and SUMOylation can drive condensate formation (*55*, *67*), we speculate that other proteins that contain phosphorylation-dependent SUMOylation motifs may also form biomolecular condensate upon phosphorylation. Therefore, the mechanism we uncover here, i.e., phase separation driven by phosphorylation-dependent SUMOylation could be generally used in other settings. Intriguingly, Stk36 does not contain a predicted pair of IPS and SIM. However, our recent studies showed that Stk36 formed a complex with another Fu/Ulk family member Ulk4, a pseudokinase, to regulate Shh signaling, and that Stk36-mediated phosphorylation and SUMOylation of Ulk4 promoted the formation of Ulk4-Stk36-Sufu-Gli2 condensates at the ciliary tip (*68*, *69*).

Our finding that signal-induced kinase condensation drives conformational change of the kinase to allosterically activate its substrate uncovers a previously uncharacterized role of biomolecular condensates. While the precise mechanism by which Fu/Ulk3 condensation drives their conformational maturation and how Fu maturation changes the conformation of Ci-Sufu subcomplex remain to be explored by future study, Fu/Ulk3 conformational maturation appears to be regulated by SUMOylation remodeling as pharmacological inhibition of either SUMOylation or deSUMOylation prevented Fu from maturation in our in vitro reconstitution experiments. We speculate that deSUMOylation is required for the breakdown of interdimer SUMO-SIM interaction to free mature Fu from the condensates, whereas re-SUMOylation may create new intradimer SUMO-SIM interaction to facilitate Fu conformational maturation through molecular cross-linking (Fig. 8). Unlike most biomolecular condensates that are formed through LLPS, Hh-induced kinase condensates exhibited slow dynamics and were resistant to the treatments that normally disrupt LLPS. We speculate that formation of more rigid and less dynamic condensates might reduce the energy barrier for Fu/Ulk3 conformational change.

In the textbook model, Hh converts Ci^F^/Gli^F^ into Ci^A^/Gli^A^ by dissociating it from Sufu, allowing freed Ci^A^/Gli^A^ to enter the nucleus to activate Hh target genes. This view is based on genetic evidence that removal of Sufu suppressed *fu* mutant phenotypes in *Drosophila* and led to constitutive activation of Hh signaling in mammals (*8*, *37*, *70*, *71*), as well as biochemical evidence that Hh signaling reduces the binding of Sufu to Ci/Gli (*29*, *38*, *72*, *73*). Here, we provide evidence that nuclear Ci^A^/Gli^A^ may exist in a complex containing phosphorylated Ci/Gli, mature Fu/Ulk3, and Sufu (Fig. 8). By conducting in vitro transcription experiments using a chromatin-assembled *ptc* promoter, we demonstrated that endogenous Ci-Sufu-Fu complexes isolated from the nucleus of Hh-stimulated cells were transcriptionally active, whereas Ci-Sufu-Fu complexes isolated from the cytoplasm were transcriptionally inert. Both Fu/Ulk3 and Sufu co-bind with Ci/Gli2 on Hh target promoters, and their chromatin association depends on Ci/Gli2. Furthermore, preventing Fu nuclear localization impeded Ci^A^ function both in vitro and in vivo because of the sequestration of Ci^A^ complex in the cytoplasm. Our findings are in line with previous studies showing that Sufu entered the nucleus in Hh receiving cells in *Drosophila* wing discs and that Sufu functioned as a chaperon to accompany Gli into the nucleus and co-bind with Gli on Hh target promoters in mammalian cells (*74*, *75*). Consistent with our findings, a more recent study showed that Ulk3 formed a complex with Gli2 in the nucleus in cancer-associated fibroblasts (*76*). Formation of an active Ci/Gli-Sufu-Fu/Ulk3 transcriptional complexes can prevent premature loss of Ci^A^/Gli^A^ function because unbound Ci/Gli is more accessible to HIB/speckle-type POZ protein (SPOP) mediated degradation in the nucleus (*37*, *48*, *77*, *78*), which could explain why ectopic Hh pathway activation in *Sufu* mutants or *Sufu Ptch1* double mutants was less dramatic than that in *Ptch1* mutants (*37*, *71*, *75*), and why increasing Sufu gene dosage could promote medulloblastoma tumorigenesis caused by *Ptch1* ablation (*79*). Because aberrant Gli activation has been implicated in many types of cancer, our findings that Ci/Gli activation is regulated by signal-induced kinase condensation and the formation of transcriptional complexes suggest that targeting these processes may provide avenues for cancer drug development.

## MATERIALS AND METHODS

### *Drosophila* mutants and transgenes

*fu^M1^* is a null allele of *fu* (*27*). Transgenic flies expressing *UAS-Myc-Fu^WT^*, *UAS-Myc-Fu^K33R^*, *UAS-Myc-Fu^S482A^*, *UAS-Myc-Fu^AAV^*, *UAS-Myc-Fu^K490R^*, *UAS-Myc-Fu*^∆*SIM*^, or *UAS-Myr-Fu* were generated using the *phiC31* integration system with the transgenes inserted into the *75B1 attP* locus. Myc-Fu variants with point mutations were generated using polymerase chain reaction (PCR)–based site-directed mutagenesis. Myristoylated Myc-Fu and HA-Fu constructs were made by addition of a stretch of amino acids harboring a myristylation signal (MGNKCCSKRQ) at the N-terminal end of the Fu constructs. CC-Fu was generated by adding peptide corresponding to leucine zipper of yeast GCN4 to the N terminus of Fu coding sequence. Fu^Ins^ constructs were made by replacing the coding sequence targeted by Fu dsRNA with a synthesized DNA fragment with alternative codon for the same primary sequence (Genescript, NJ). To generate the Cl8 cell lines stably expressing Fu constructs, Fu (WT or mutants) cDNAs were subcloned into the *pMT/V5-His A* vector using NotI and KpnI sites with 3× HA tag attached on the N terminus. All baculoviral constructs were made with *pFastBac1* as the backbone using EcoRI and KpnI sites. All GST-fusion constructs were made by subcloning coding sequences into the *pGEX 4 T-1* vector using EcoRI and XhoI sites. Mammalian cell expression constructs were subcloned into *pcDNA3.1(+)* vector using EcoRI and XbaI sites or into the lentiviral vector, *FUXW*, using BamHI and XbaI sites. All Ulk3 constructs were made by adding a C-terminal 3× HA tag to the coding sequence of mouse Ulk3. Myc-Gli2/Flag-Gli2 were made by adding 6× Myc/3× Flag tag to the N-/C-terminal end of mouse Gli2 coding sequence.

### Cell cultures, transfection, and lentiviral infection

Cells (described as S2 cells in the text for simplicity) were cultured in *Drosophila* SFM (Thermo Fisher Scientific) supplemented with 10% fetal bovine serum (FBS) (MilliporeSigma, F4135) at 24°C. Cl8 cells were cultured in Shields and Sang M3 medium (MilliporeSigma) with 2.5% of FBS, 2.5% fly extract, and insulin (0.5 mg/ml) (MilliporeSigma). Sf9 cells were cultured in SF900III medium containing 10% FBS at 27°C. NIH3T3 cells were cultured in Dulbecco’s modified Eagle’s medium (DMEM, MilliporeSigma) containing 10% bovine calf serum [American Type Culture Collection (ATCC)]. HEK293T cells were cultured in DMEM containing 10% FBS (Thermo Fisher Scientific). Transfection of S2R^+^ cells was performed using calcium phosphate. Cl8 cells were transfected using CellFectin II Reagent (Thermo Fisher Scientific). Mammalian cells were transfected with PolyJet in vitro DNA transfection kit (SignaGen). For lentivirus production, HEK293T-17 (ATCC) cells were seeded and transfected with psPAX2 and vesicular stomatitis virus glycoprotein, along with pLKO.1-puro [for short hairpin RNA (shRNA) lentivirus] or FUXW (for protein-expressing lentivirus). After 48 hours, viruses containing culture media were collected, filtered, and centrifuged at 20,000*g* for 2 hours, and the resulting precipitates were resuspended in culture medium and stored in aliquots at −80°C for future use. Gli2 shRNA (TRCN0000219066) and Kapβ2 shRNA (TRCN0000295586) were obtained from MilliporeSigma. Hh-conditioned medium was prepared by culturing S2 cells stably expressing Hh-N with 0.7 mM CuSO4 induction for 1 day. The resultant medium was filtered and harvested. Hh-conditioned medium was used at 8:2 ratios with regular medium. Recombinant human sonic Hh protein (Shh-N) was purchased from R&D systems (8908-SH) with final concentration of 10 ng/ml.

### Generation of Cl8 cell lines stably expressing Fu constructs

Cl8 cells were transfected with *pMT/V5-His A* vectors containing the coding sequences of HA-Fu^INS^ (WT or mutants) and the *pCoHygro* vector. Two days after transfection, the cells were subject to hygromycin B (250 μg/ml) selection for 2 weeks, and individual cells were sorted by flow cytometry and further expanded. The expression level of exogenous Fu protein in each positive colony was measured by immunoblot with Fu antibody after treatment with Fu dsRNA and CuSO_4_. Colonies with HA-Fu^INS^ constructs expressed at levels similar to that of the endogenous Fu were picked and stored for further experiments.

### Generation knock-in NIH3T3 cell line using CRISPR-Cas9

NIH3T3 cell was electroporated with the *PX458* vector containing SgRNA GCTCCATCCGCTGCTCCTTC (−) targeting mouse *Ulk3* and *SpCas9*, and a *pUC19* vector with the following cassette inserted: left arm (Ulk3 C-terminal coding sequencing with stop codon mutated) – 3× HA tag – P2A – blasticidin resistant gene – right arm. After 2 days, the resultant cells were subject to blasticidin (10 μg/ml) selection for 2 weeks and single cell was sorted by flow cytometry and further expanded. The resulting cell colonies were analyzed by immunoblot with both HA and Ulk3 antibodies for validation. Similar protocol was applied to tagging endogenous Gli2 (SgRNA: TTTTAAACATGATGACCTAA+) with C-terminal 3× Flag except that Puromycin (5 μg/ml) was used for selection.

### Immunostaining, IP, and Western blot analysis

For immunostaining of cells, harvested cells were first washed with phosphate-buffered saline (PBS) and fixed with 4% formaldehyde in PBS for 30 min at 37°C [except for pGli2 staining, cells were fixed with methanol/acetone (1:1) at −20°C for 10 min]. Fixed cells were permeabilized with PBS containing 0.2% Triton X-100 for 30 min at 37°C. After being washed for three times with PBS, the cells were incubated with primary antibodies in PBS for 1.5 hours at room temperature or 4°C overnight. Secondary antibody incubation was carried out at room temperature for 1 hour. For NIH3T3 cells, a similar protocol was adopted except that the cells were seeded in Nunc Lab-Tek II CC2 Chamber Slide (Thermo Fisher Scientific). Immunostaining of imaginal discs, IP, and Western blot analysis were carried out using the standard protocols.

To reveal the mobility shift of phosphorylated Sufu, resolving gel (T value12.5%; C value 0.83%) and stacking gel (T value 5%; C value 1.33%) mixtures were prepared. Images were captured by confocal microscopy (Zeiss LSM700) and fluorescence signals were quantified by ImageJ software with neighboring background noise eliminated. Antibodies used in this study were as follows: mouse anti-Flag (M2, F3165, MilliporeSigma), mouse anti-HA (F7, Santa Cruz Biotechnology), rabbit anti-HA (ab9110, Abcam), mouse anti-Myc (9E10, Santa Cruz Biotechnology), rabbit anti-Myc (ab9106, Abcam), rat anti-Ci (2A1, DSHB), mouse anti-Fu (22F10, DSHB), mouse anti-Sufu (25H3, DSHB), mouse anti-Patched (Apa1, DSHB), mouse anti-engrailed (4D9, DSHB), mouse anti–α-tubulin (T9026, MilliporeSigma), rabbit anti-histone3 (ab1791, Abcam), goat anti-Gli2 (AF3635, R&D systems), rabbit anti-SUMO1 (4930, Cell Signaling Technology), mouse anti-acetylated tubulin (T7451, MilliporeSigma), mouse anti-ubiquitin (P4D1, Santa Cruz Biotechnology), and rabbit anti-Sufu (ab28083, Abcam). Phospho-Fu antibodies: pT154/S159 and pT158/S159 (*26*); phospho-Ci antibodies: pS218/S220 (*29*) and S1382 (pCi) (*31*); phospho-Gli2 antibody: pS230/232 (*29*). The phospho-Fu antibody (pS482) was generated by Genemed Synthesis (San Antonio, TX) using the following phospho-peptide as antigen: QLKHS(p)MHSTNEEKL. This phospho-specific antibody was purified by positive and negative selection using affinity column conjugated with the antigen peptide and corresponding nonphosphorylated peptide sequentially.

### Protein purification

GST-fusion proteins were expressed in *BL21*, purified with Glutathione Sepharose 4B (GE Healthcare), and eluted with 10 mM reduced Glutathione. For protein purification using the baculovirus system, Sf9 cells were infected with corresponding baculovirus at the density of 1 to 2 × 10^6^/ml, and infected cells were collected 2 days after the infection. The resultant cell lysates were incubated with Flag M2 (MilliporeSigma) or HA (Pierce) affinity agarose and eluted with corresponding synthetic peptides. A similar protocol was applied to proteins expressed in HEK293Tcells.

### RNAi by dsRNA

dsRNAs were generated using the MEGAscript high-yield transcription kit (Ambion). For transfection, cells (or Cl8) were seeded at the density of 1 × 10^6^/ml with serum-free medium; dsRNA (10 to 20 μg) was added for each 10-cm plate, and transfected cells were further incubated for 2 to 3 hours before switching to regular growth medium. The primers for amplifying DNA template for dsRNA transcription are as follows. Ci: GAATTAATACGACTCACTATAGGGAGAATCAACGTTAGAAGGCGGTG (F) and GAATTAATACGACTCACTATAGGGAGACGCTTACAGCGGAAAGATTC (R).

Fu: GAATTAATACGACTCACTATAGGGAGATGTTGAGCATCTTGAGACC (F) and GAATTAATACGACTCACTATAGGGAGAGACCCAAGCGAACAGAGAAG (R).

Sufu: GAATTAATACGACTCACTATAGGGAGAATGGCCGAGGCGAATTTGGA (F) and GAATTAATACGACTCACTATAGGGAG-AGTTAGTCACCAGCCAATCAC (R).

HIB: GAATTAATACGACTCACTATAGGGAGATTCAAGAA-GTTCATCCGACG (F) and GAATTA ATACGACTCATATAGGGA GATGGTGGGATTTGTTGTGTTG (R).

### Dual luciferase reporter assay, qRT-PCR, and PLA

S2R^+^ cells were seeded in 12-well plate at the concentration of 1 × 10^6^/ml, and, the next day, each well was transfected with 0.5 μg of *ptc-Luc* reporter construct, 25 ng of *RL-PolIII Renilla* internal control construct, and 0.5 μg each of the other constructs. The cells were collected 48 hours after the transfection. For *Gli-luc reporter* assay, NIH 3T3 cells were seeded in 12-well plate and transfected with 0.4 μg of *8XGli1-Luc* reporter construct and 0.1 μg of *PRL-SV40 Renilla* construct, together with 0.5 μg each of other constructs. The luciferase activity was determined using dual-luciferase reporter assay system (Promega) and FLUOstar OPTIMA (BMG Labtech). For reverse transcription quantitative PCR (RT-qPCR) with cell samples, total RNA was extracted from cells using RNeasy Plus Mini Kit (Qiagen), cDNA was synthesized with iScript cDNA synthesis kit (Bio-Rad), and qPCR was performed using iQ SYBR Green System (Bio-rad) and a Bio-Rad CFX96 real-time PCR system. Mammalian glyceraldehyde-3-phosphate dehydrogenase (GADPH) or *Drosophila* Rps20 expression level was used as a normalization control. The primer pairs used were as follows:

*GADPH*: GTGGTGAAGCAGGCATCTGA(F) and GCCATGT-AGGCCATGAGGTC(R).

*Gli1*: GTGCACGTTTGAAGGCTGTC(F) and GAGTGGGTC-CGATTCTGGTG(R).

*Ptch*1: GAAGCCACAGAAAACCCTGTC(F) and GCCGCAA-GCCTTCTCTAGG(R).

*ptc*: ATGGACCGCGACAGCCTCCCA(F) and CGACGCAGA-AGGTGCTCAGCA(R).

*Rps20*: TGTGGTGAGGGTTCCAAGAC(F) and GACGATCTC-AGAGGGCGAGT(R).

PLA was performed by using Duolink in situ detection reagents (MilliporeSigma) following the guidance of the manufacturer.

### In vitro kinase assay

Flag-FuN^EE^ and Flag-FuN^GV^ expressed in Sf9 cells using the baculovirus system (Bac to Bac, Thermo Fisher Scientific) were purified by Flag M2 affinity agarose (MilliporeSigma) and eluted with Flag peptide (200 μg/ml). HA-mUlk3^WT^ and HA-mUlk3^KR^ were expressed in HEK293T cells by transient transfection, purified with Pierce anti-HA agarose (Thermo Fisher Scientific), and eluted with HA peptide (250 μg/ml). GST-fusion proteins were expressed in BL21, purified with Glutathione Sepharose 4B (GE Healthcare), and eluted with 10 mM reduced Glutathione. CK1δ was purchased from NEB (#P6030). In vitro kinase assay was conducted by incubating reaction mixture (25 μl) containing 150 mM tris-HCl (pH 7.5), 0.2 mM Mg^2+^/ATP, 1 μg of substrate (GST or GST-fusion proteins), and appropriate amount of protein kinase at 30°C for 30 min. In the case of Fu (or Ulk3)/CK1 sequential kinase assay, glutathione beads preabsorbed with GST or GST-fusion proteins were firstly incubated with either FuN or Ulk3 for 15 min and then centrifuged and washed thoroughly by 1× kinase assay buffer for three times. The resultant beads were further used as substrates for second round of kinase assay with CK1. The reaction was terminated by adding 2× SDS loading buffer. For in vitro kinase assay in presence of Sufu, 2.5 μg of purified Sf9-derived HA-Sufu (S321A) was added in the reaction mixture. The phosphorylation of substrates was analyzed by pIMAGO with fluor-680 detection kit (MilliporeSigma) and LI-COR Odyssey platform as previously described (*29*).

### In vitro SUMOylation and deSUMOylation assay

In vitro SUMOylation assay was performed with SUMOylation assay kit (Abcam) following the manufacturer’s instructions. HA-tagged Fu or Ulk3 fragments were transiently expressed in HEK293T cells and purified with Pierce anti-HA agarose. The resulting agarose were subjected to sequential Fu(Ulk3)/CK1 kinase assay before finally used as substrates in SUMOylation assay. For deSUMOylation assay, yeast C-terminal 204 amino acid fragment containing Ulp1 catalytic domain (Ulp1-C204) and its C580S variant (enzymatically dead form) was subclone into pGEX 4 T-1 vector. GST-Ulp1-C204 and GST-Ulp1-C204^C580S^ were expressed in BL21 *E. coli* and purified with Glutathione Sepharose 4B. In vitro deSUMOylation assay was carried out by mixing purified Ulp1 proteins with Cl8 cell lysate in buffer containing 50 mM tris-HCl (pH 7.5), 150 mM NaCl, and 1 mM dithiothreitol (DTT) at 37°C for 1 hour. The reaction was stopped by adding equal volume of 2× SDS loading buffer and boiled for 5 min.

### Cell fractionation

Cl8 cells were washed with PBS and then resuspended in appropriate volume of buffer containing 20 mM Hepes (pH 7.6), 5 mM MgCl_2_, 10 mM KCl, 250 mM sucrose, and 0.025% digitonin (EMD Bioscience). The cells were further passed through 18_1/2_G syringes three times and incubated at 4°C for 5 min with agitation. Following a centrifugation at 800*g* for 10 min, the resultant supernatant was collected as cytoplasmic fraction. The remaining pellet was extracted with 10 mM tris-HCl (pH 7.5), 300 mM NaCl, and 1% NP 40 to yield nuclear fraction.

### Sedimentation

Cl8 cells with or without Hh conditional medium treatment were disrupted with nitrogen decompression (1500 psi, 15 min) on ice and subjected to 3000*g* centrifugation for 15 min. The resulting supernatant was further spun down at 100,000*g* for another 15 min. The pellet (P100) and supernatant (S100) were collected as Fu condensate and soluble Fu containing fractions.

### Solubilization of Fu condensates

Fu condensates from either Cl8 cells treated with Hh-conditioned medium or Sf9 cells expressing Fu^EE^ were collected by differential centrifugation. The condensates (P100 fractions) were resuspended in 10 mM tris-HCl (pH 7.5) and extracted with reagents at room temperature or 4° for 30 min with agitation. The soluble and insoluble parts were collected with second round sedimentation (100,000*g* for 15 min).

### Sequential IP, BN-PAGE electrophoresis, and limited proteolysis

The cytoplasmic and nuclear fraction of Cl8 cells were firstly incubated with Ci antibody (2A1, DSHB)/Protein G beads and then eluted with GST Ci 700 to 850 fragment (1 μg/μl) (antigen of Ci 2A1 antibody, expressed and purified from BL21 *E. coli* cells) in buffer containing 20 mM bis-tris (pH 7.0) and 50 mM NaCl. For secondary IP, the elute was incubated with Sufu antibody (25H3, DSHB)/Protein G beads. The bound protein was stripped with 2× SDS loading buffer and boiled for 5 min. To perform BN-PAGE electrophoresis, Ci complex was mixed with NativePAGE sample buffer and NativePAGE G-250 and incubated on ice for 10 min. The samples were then loaded onto NativePAGE Novex 3 to 12% gel and subjected to further immunoblot analysis. Limited proteolysis was carried out by mixing samples with trypsin (0.5 μg/ml) at 37°C; a small aliquot was withdrawn at each time point, and protease was inactivated by adding SDS loading buffer.

### Electroelution and disruption of SDS-resistant Fu dimer

Sf9 cells were infected with Flag-Fu^EE^ (1:20,000, v:v) and HA-SUMO (1:5,000, v:v) baculovirus with low MOI and Flag-Fu^EE^ protein was purified with Flag M2 affinity agarose and load on a 6% SDS PAGE (pre-run). The band containing Fu protein was cut blindly on the basis the molecular weight marker and further smashed into small pieces. The electroelution was performed using an Elutrap system (Cytiva) in a standard tris-glycine buffer containing 0.01% SDS at 4°C for 20 hours. The elute was concentrated with ultracentrifugation and incubated in 2× SDS loading buffer at −18°C for 4 to 8 days.

### Chromatin assembly and in vitro transcription assay

The DNA fragment for *ptc* promoter region (−758 to +130) was chemically synthesized (Genescript) and subcloned into pGL2 basic luciferase reporter. The DNA region including the *ptc* promoter region plus a part of Luciferase coding sequence (around 200 base pairs) was amplified using a set of primers with biotin modification, and the resulting PCR products were used for chromatin assembly. Fifty microliters of preabsorbed Streptavidin beads was mixed with 1 μg of gel-extracted biotinylated linearized DNA in buffer [20 mM Hepes (pH 7.5), 2 M NaCl, 1 mM EDTA, and 10% glycerol] at room temperature for 1 hour with agitation. Then, the beads were thoroughly washed with HEG50 buffer [25 mM Hepes (pH 7.6), 50 mM KCl, 0.1 mM EDTA, and 10% glycerol] twice and resuspended in the same buffer. The chromatin assembly was conducted with a chromatin assembly kit (Active Motif) following the manufacturer’s instructions. Partial digestion with micrococcal nuclease was performed to ensure the quality of chromatin assembly. One hundred nanograms of of chromatin DNA was mixed with 20 ng of purified cytoplasmic or nuclear Ci complex (200 ng of Sufu was also added to capture any free Ci due to dissociation) in HAT buffer [20 mM tris-HCl (pH 7.6), 50 mM KCl, 5 mM DTT, 10 mM sodium butyrate, 2 mM MgCl_2_, and 5% glycerol] at 30°C for 20 min. Histone acetylation was conducted by adding 15 ng of dCBP purified from Sf9 cells and 2 μM acetyl–coenzyme A followed by 30°C incubation for 30 min. Thirty microliters of HAT reaction mixture was combined with 27 μl of transcription mixture: 6 μl of transcription buffer [200 mM Hepes (pH 7.9) and 40 mM MgCl2], 0.48 μl of 1 M DTT, 1.22 μl of bovine serum albumin (10 mg/ml), 0.3 μl of RNasin (10 U/ml), 6 μl of BC150 buffer [20 mM tris-HCl (pH 7.9), 0.2 mM EDTA, 150 mM KCl, and 20% glycerol], and 100 μg of nuclear extract, and incubated at room temperature for 20 min to from preinitiation complex. Then, 3 μl of nucleotide triphosphate mixture was added to the mixture and incubated at 30°C for 50 min. After the in vitro transcription reaction, streptavidin beads were removed by centrifugation, and the resultant supernatant was treated with 20 U of deoxyribonuclease I (DNase I) at 37°C for 20 min. The RNA transcripts were extracted with TRIzol and chloroform and then redissolved in ribonuclease-free water and treated with 10 U of DNase I at 37°C for another 20 min. After heat inactivation of DNase I, 2 μl of RNA was analyzed by one-step RT-qPCR with primers: AGGCCCGGCGCCATTCTATC(F) and AGCAATTGTT-CCAGGAACCA(R).

### Nuclear extract preparation

Four hundred milliliters of cells at the density of 2 to 3 × 10^6^/ml were collected and washed twice with cold PBS. The cells were thoroughly resuspended in lysis buffer [20 mM Hepes (pH 7.9), 1 mM MgCl_2_, 10 mM KCl, 1 mM DTT, 0.1 mM EDTA, and 1× Complete protease inhibitor cocktail] and incubated on ice for 10 min. The cells were then homogenized with a motor-driven homogenizer for 15 to 20 times on ice. The nuclei were collected by 1000*g* centrifugation for 15 min and washed twice with lysis buffer. The nuclei pellet was resuspended in low-salt nuclear extraction buffer [20 mM Hepes (pH 7.9), 1 mM MgCl_2_, 20 mM KCl, 1 mM DTT, 0.1 mM EDTA, 25% glycerol, and 1× Complete protease inhibitor cocktail] by two to three gentle strokes in glass Dounce homogenizer with a type B pestle. With gentle stirring on ice, one-third volume of high-salt nuclear extraction buffer [20 mM Hepes (pH 7.9), 20 mM MgCl_2_, 1.2 M KCl, 1 mM DTT, 0.1 mM EDTA, 25% glycerol, and 1× Complete protease inhibitor cocktail] was added to the above suspension. The extract was carried out by gentle stirring and agitation for 30 min on ice. After 10,000*g* centrifugation for 10 min, the supernatant was collected and dialyzed against the stocking buffer [20 mM Hepes (pH 7.9), 100 mM KCl, 1 mM DTT, 0.1 mM EDTA, 20% glycerol, and 1× Complete protease inhibitor cocktail] for 2 hours at 4°C. The resultant nuclear extract was aliquoted and store at −80°C.

### ChIP qPCR

Cl8 cells were treated with 1% fresh formaldehyde at room temperature with gentle shaking for 15 min. The cross-linking was stopped by adding glycine to a final concentration of 0.125 M with mild swirling. The cells were scraped off the plate and washed with cold PBS twice and resuspended with cold Farnham lysis buffer [5 mM Pipes (pH 8.0), 85 mM KCl, 0.5% NP-40, and 1× complete protease inhibitors cocktail]. The cells were subjected to centrifugation, and the pellet was resuspended in cold Farnham lysis buffer. The lysates were passed through 20-gauge needle for 15 to 20 times. The nuclei were collected by centrifugation and resuspended in cold radioimmunoprecipitation assay buffer (1× PBS, 1% NP-40, 0.5% sodium deoxycholate, and 0.1% SDS). The resulting samples were sonicated by a Bioruptor for 5 min in total (30 s on and 30 s off). The supernatants were collected after centrifugation and mixed with protein G beads preabsorbed with appropriate antibodies at room temperature for 1 hour and 4°C for another hour. The beads were washed with LiCl wash buffer [100 mM tris (pH 7.5), 500 mM LiCl, 1% NP-40, and 1% sodium deoxycholate] five times and resuspended with IP elution buffer (1% SDS and 0.1 M NaHCO_3_). The beads were incubated in 65°C water bath for 1 hour and shacked for every 15 min. The supernatants were collected after centrifugation and incubated in 65°C water bath overnight. After the final purification with QIAquick PCR purification kit, the resultant DNA was used for RT-qPCR. The primers used are as follows:

*ptc*: CTACAGTGGCAACAACAAAC(F) and CACGAGAGCG-CTAATTTCTA(R).

*Gli1*: TATGGGGTTGGGAGAGTTTG(F); AAAGAGACCTGG-GACAGACAC(R).

*Ptch*1: GGGTTGCCTACCTGGGTGGTCT(F) and AACGCG-ATTGGCTCTTGGAG(R).

### In vitro assay for Fu condensation

Flag-Fu^K33R^ expressed in Sf9 cells was purified by Flag M2 affinity agarose and phosphorylated in vitro by HA-FuN^EE^ and CK1. Phosphorylated Fu was repurified with Flag beads and used as substrate for in vitro SUMOylation assay in the absence or presence of SUMO1 at 37°C. A small aliquot of reaction mixture was withdrawn at different time points and subjected to 100,000*g* centrifugation for 15 min. The supernatant and pellet were collected and subjected to Western blot analysis.

### In vitro dissolution of Fu condensates

Fu condensates were collected by differential centrifugation of Sf9 cells expressing Fu^EE^ and sequentially washed with 0.3 M NaCl and 1% Triton X-100 for 30 min at room temperature. Depolymerization was performed by mixing Fu condensates with GST-Ulp1-C204 or GST-Ulp1-C204^C580S^ in DeSUMOylation buffer at 37°C. A small aliquot of reaction mixture was withdrawn at different time points and subjected to 100,000*g* centrifugation for 15 min. The supernatant and pellet were collected for further analysis by Western blot.

### In vitro Fu/Ulk3 maturation and Ci/Gli2 activation

Soluble (S100) and condensate fraction (P100) of Fu Kinase were collected from Sf9 expressing Fu^EE^ at high MOI (5 to 10) by differential centrifugation. Soluble Fu was further purified by Flag M2 beads, while Fu condensate was sequentially washed with 0.3 M NaCl and 1% Triton X-100 for 30 min at room temperature. The maturation was carried out by mixing similar amounts (~25 μg) of soluble Fu and Fu condensate with MPA [Sf9 total cell lysate in buffer containing 20 mM tris-HCl (pH 7.5), 100 mM NaCl, 2.5 mM DTT, 5 mM EDTA, 3 mM ATP, 30 mM phosphocreatine, creatine phosphokinase (20 μg/ml), and 0.1 μM SUMO 1] at room temperature with agitation. A small aliquot of the reaction mixture was withdrawn at different time points and subjected to 100,000*g* centrifugation for 15 min. The supernatant and pellet were collected for further analysis by Western blot. When coupled with Ci phosphorylation, roughly 100 to 200 ng of Flag-Ci^-PKA^ and HA-Sufu (S321A) complex was incubated with either soluble Fu or Fu condensate in the same buffer with or without adding MPA at room temperature for 6 hours. Soluble and condensate fractions were collected from the reaction mixture after 100,000*g* centrifugation for 15 min and subjected to Western blot analysis. Similar protocol was applied to in vitro Ulk3 maturation and Gli2 phosphorylation except that all proteins were expressed and purified from HEK293T cells and HEK293T total cell lysates were used in MPA.

### FRAP assay

To evaluate the dynamics Hh-induced Fu condensates, Cl8 cells were transfected with mEGFP-Fu and treated with Hh-conditioned medium for 16 hours. To perform the FRAP assay, pre-photobleaching image was recorded followed by quickly photobleaching the region of interest to ~25% of its original intensity. Subsequently, images were acquired every 5 min using a Nikon A1R confocal microscope (Ti-E) equipped with a CFI SR Apochromat TIRF 40×/1.49 oil objective lens. The mean fluorescence intensity of the photobleached region was calculated using ImageJ software, and the un-photobleached region in the vicinity was used to correct the curve.

### Proximity ligation assay

PLA (*52*) was carried out using Duolink In Situ Detection Reagents Red (MilliporeSigma, #DUO92008). The cells were fixed with 4% formaldehyde, permeabilized with PBST (0.2% Triton X-100), and blocked with PLA blocking solution. After sequential incubation with a pair of primary antibodies and corresponding PLA probes (PLUS and MINUS), the cells were further subjected to ligation/amplification reaction. PLA images were obtained with Zeiss LSM 700 confocal microscope.

### Quantification and statistical analysis

All experiments were carried out independently two to three times as described in the figure legends. All quantification data were analyzed by two-tailed Student’s *t* test. *P* values are described in the figure legends.

## Supplementary Materials

### This PDF file includes:

Figs. S1 to S7 Uncropped Western Blots
