# Supplementary material for: Morphogen-induced kinase condensates transduce Hh signal by allosterically activating Gli
Source: Sci Adv. 2025 Jan 10;11(2):eadq1790. doi: 10.1126/sciadv.adq1790 (PMC11721587; doi:10.1126/sciadv.adq1790)

Science Advances
AAAS

# Supplementary Materials for

## Morphogen-induced kinase condensates transduce Hh signal by allosterically activating Gli

Yuhong Han *et al.*

Corresponding author: Jin Jiang, jin.jiang@utsouthwestern.edu

*Sci. Adv.* **11**, eadq1790 (2025)
DOI: 10.1126/sciadv.adq1790

**This PDF file includes:**

Figs. S1 to S7
Uncropped Western Blots

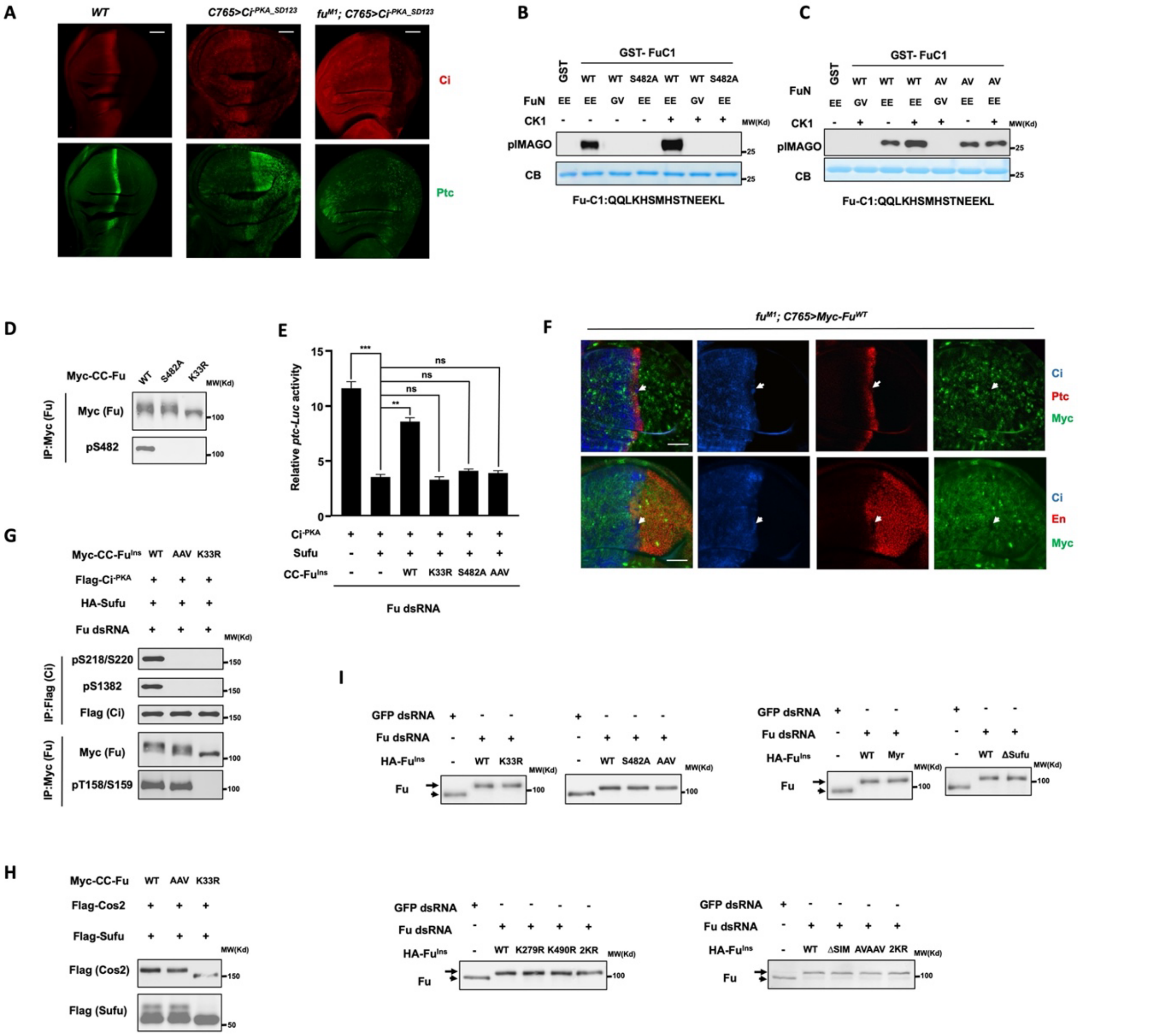

## Fig. S1. Characterization of Ci and Fu variants.

(**A**) Late third instar wing discs of the indicated genotypes were immunostained to show the expression of Ci (red) and Ptc (green). All wing discs are oriented with anterior to the left and ventral up. Scale bars are 50 μm. *ci* is expressed in the anterior (A) compartment whereas *hh* is expressed in the posterior (P) compartment. Consequently, *ptc* is activated in A-compart cells near the A/P boundary in wild type (*WT*) wing discs. *C765>Ci*$^{-PKA\_SD123}$ induced ectopic expression of Ptc in A-compartment cells away from the A/P boundary as well as in P-compartment cells where Hh is produced. Of note, the ectopic Ptc expression is higher in the P-compartment than that in the A-compartment. The ectopic Ptc expression is downregulated in the P-compartment to reach a level comparable to that in the A-compartment in *fu* mutant discs, suggesting that Fu is required for Hh to upregulate the activity of Ci$^{-PKA_SD123}$.

(**B**-**C**) *In vitro* kinase assays using constitutively active (EE) or kinase dead (GV) Fu kinase domain (FuN; aa1-305) purified from Sf9 cells and recombinant CK1 as kinases, and GST fusion proteins containing wild type (WT) or mutated (S482A or AV: S485A/T486V) Fu-C1 fragment as substrates. Phosphorylation was detected by the pIMAGO system. CB: Coomassie blue staining.
(**D**) Western blot analysis of the phosphorylation of wild type (WT) and the indicated mutant Myc-CC-Fu expressed in S2R+ cells.
(**E**) *ptc-luc* reporter assay in Fu-depleted S2R+ cells expressing the indicated constructs. Endogenous Fu was depleted by Fu dsRNA and RNAi insensitive (Ins) Fu constructs were used for transfection. Data are mean ± SD from three independent experiments. $^{**}P < 0.01$, $^{***}P < 0.001$ (t test). ns: not significant.
(**F**) Late third instar wing discs of $fu^{M1}$ mutant flies expressing Myc-$Fu^{WT}$ under the control of the *C765* Gal4 driver were immunostained to show the expression of Ci, Ptc or En, and Myc. Of note, the transgenic expression driven by *C765* is uneven. Arrowheads indicate cells expressing relatively low levels of Myc-$Fu^{WT}$. Scale bars are 50 μm.
(**G**) Western blot analysis of the phosphorylation of $Ci^{-PKA}$ and RNAi-insensitive CC-Fu (wild type and mutants) co-expressed with HA-Sufu in Fu-depleted S2R+ cells.
(**H**) Western blot analysis of the phosphorylation of Cos2 and Sufu coexpressed with wild type or mutant CC-Fu (AAV or K33R) in S2R+ cells. The phosphorylation of Cos2 and Sufu was indicated by mobility shift.
(**I**) Western blot analysis of Fu in control (GFP dsRNA) or Fu-depleted Cl8 cells stably expressing the indicated RNAi-insensitive HA-Fu constructs. Arrows and arrowheads indicate exogenously expressed and endogenous Fu, respectively.

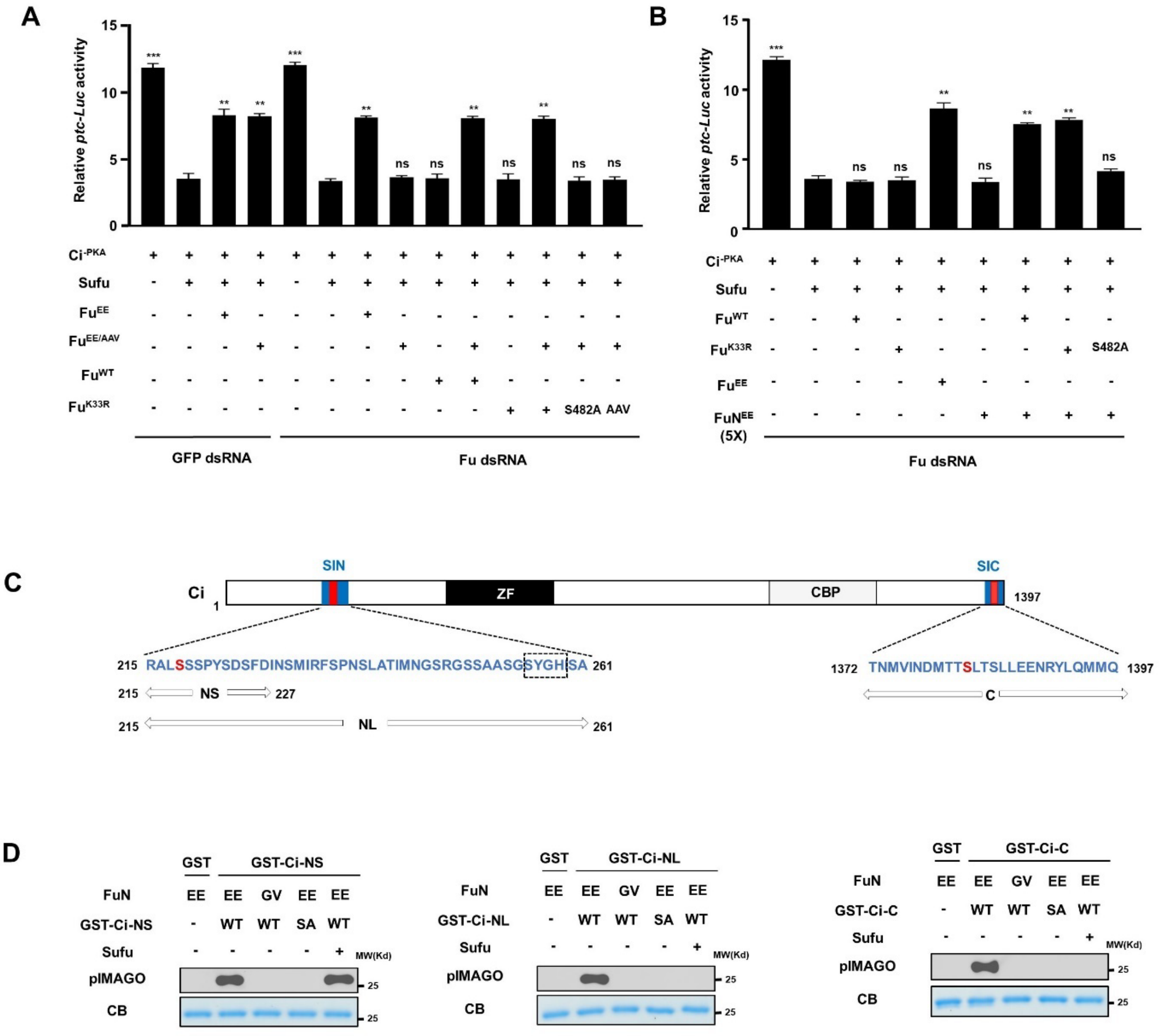


**Fig. S2. Phosphorylation of Fu regulatory domain confers a non-catalytic function of Fu required for Ci activation.**

(**A-B**) *ptc-luc* reporter assay in S2R+ cells expressing the indicated constructs and treated with GFP (control) or Fu dsRNA. All Fu constructs used are RNAi-insensitive. Data are mean ± SD from three independent experiments. $^{**}P < 0.01$, $^{***}P < 0.001$ (*t* test). ns: not significant.

(**C**) Schematic representation of Ci with Fu phosphorylation sites highlighted in red and the sequence of the N- and C-terminal Sufu Interacting domains (SIN and SIC) shown below the schematic drawing. The key residues mediated Sufu binding to SIN are boxed by dash lines. ZF: Zinc finger DNA binding domain; CBP: *Drosophila* CBP binding domain.

(**D**) *In vitro* kinase assay using constitutively active (EE), or kinase dead (GV) Fu kinase domain (FuN) purified from Sf9 cells and the indicated GST-fusion proteins as substrates in the absence or presence of purified Sufu protein. Ci-NS: aa215-227; Ci-NL: aa215-261; Ci-C: aa1372-1397.

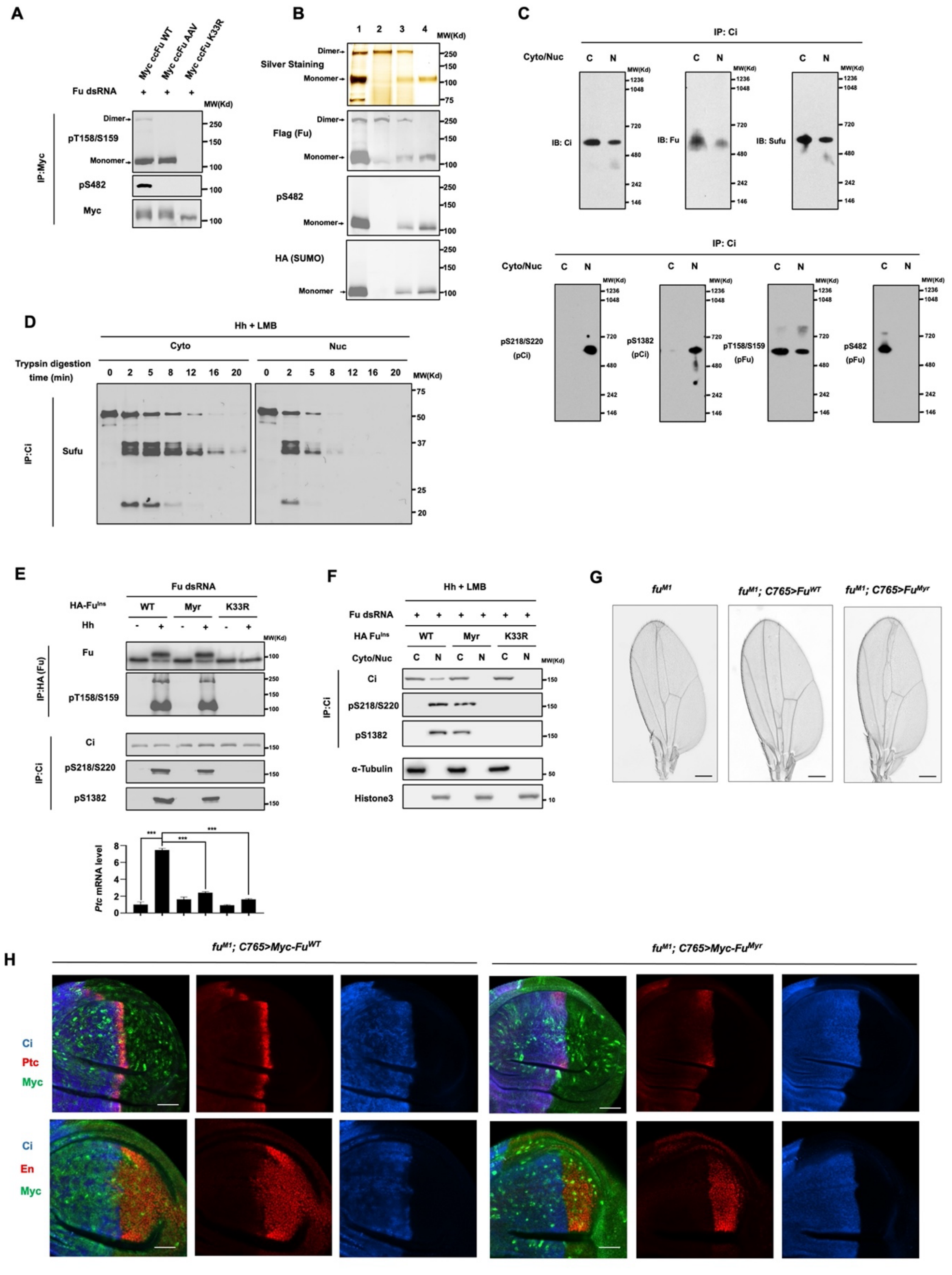

A
Myc ccFu WT
Myc ccFu AAV
Myc ccFu K33R
Fu dsRNA
IP:Myc
Dimer
pT158/S159
Monomer
pS482
Myc
MW(Kd)
B
Silver Staining
Flag (Fu)
pS482
HA (SUMO)
Dimer
Monomer
C
IP: Ci
Cyto/Nuc
IB: Ci
IB: Fu
IB: Sufu
pS218/S220 (pCi)
pS1382 (pCi)
pT158/S159 (pFu)
pS482 (pFu)
D
Hh + LMB
Cyto
Nuc
Trypsin digestion time (min)
IP:Ci
Sufu
E
Fu dsRNA
HA-Fu^Ins
WT
Myr
K33R
Hh
IP:HA (Fu)
Fu
pT158/S159
IP:Ci
Ci
pS218/S220
pS1382
Ptc mRNA level
F
Hh + LMB
Fu dsRNA
HA Fu^Ins
WT
Myr
K33R
Cyto/Nuc
IP:Ci
Ci
pS218/S220
pS1382
α-Tubulin
Histone3
G
fu^M1
fu^M1; C765>Fu^WT
fu^M1; C765>Fu^Myr
H
fu^M1; C765>Myc-Fu^WT
fu^M1; C765>Myc-Fu^Myr
Ci
Ptc
Myc
Ci
En
Myc

**Fig. S3. Characterization of mature Fu and Ci-Sufu-Fu complexes**

(**A**) Western blot analysis of phosphorylation and maturation of Fu in S2R+ cells expressing the indicated RNAi-insensitive Fu constructs and treated with Fu dsRNA. Arrow indicates the SDS resistant dimer (mature Fu).

(**B**) Silver staining and western blot analysis of Fu purified from Sf9 cells expressing Flag-Fu$^{EE}$ and HA-SUMO (lane 1). Mature form of Fu was cut from SDS-PAGE, recovered by electroelution, and incubated in 2X SDS loading buffer for 0 (lane 2), 4 (lane 3) and 8 days (lane 4), respectively. The products were subjected to electrophoresis on SDS-PAGE followed by silver staining (top panel) or western blot analysis with the indicated antibodies. pS482 and SUMO epitopes were not detected in the mature Fu (Lanes 1 and 2) but were detected in the Fu monomer derived from the mature Fu (Lanes 3 and 4).

(**C**) Ci complexes were immunopurified from the nuclear (N) and cytoplasmic (C) fraction of Cl8 cells treated with Hh-conditioned medium and LMB and subjected to electrophoresis on blue native gels, followed by western blot analyses with the indicated antibodies. Top: Sufu and Fu formed a ~600KD complex with Ci both in the cytoplasm (C) and found nucleus (N). Bottom: pS218/S220 and pS1382 were detected in Ci complex form the nucleus but not that from the cytoplasm, suggesting that active Ci is in the nuclear complex. Whereas pT158/S159 detected phosphorylated Fu in both cytoplasmic and nuclear Ci complexes, pS482 only detected phosphorylated Fu in the cytoplasmic but not in the nuclear Ci complex, consistent with the notion that the nuclear Ci complex contained the mature Fu.

(**D**) Western blot analysis of Sufu in the cytoplasmic or nuclear Ci complexes from Cl8 cells treated with Hh-conditioned medium and LMB after limited trypsin proteolysis. Sufu in the nuclear Ci complex was digested by trypsin more rapidly than Sufu in the cytoplasmic Ci complex, suggesting that Sufu in the nuclear Ci complex adopts a more “open” conformation than Sufu in the cytoplasmic complex.

(**E**) Western blot analysis of Ci phosphorylation, Fu phosphorylation and maturation (top) or qRT-PCR analysis of *ptc* expression (bottom) in Fu-depleted Cl8 cells stably expressing the indicated RNAi-insensitive Fu constructs and treated with control or Hh-conditioned medium. In contrast to kinase dead Fu (Fu-K33R) but like wild type Fu (Fu-WT), Myr-Fu was activated, underwent maturation, and activated Ci in response to Hh. Data are mean ± SD from two independent experiments. ***$P < 0.001$ (*t* test).

(**F**) Western blot analysis of cytoplasmic (C) and nuclear (N) Ci and phosphorylated Ci from Fu-depleted Cl8 cells stably expressing the indicated RNAi-insensitive Fu constructs and treated with Hh-conditioned medium and LMB. Unlike Ci activated by Fu-WT (recognized by pS218/S220 and pS1382), which entered the nucleus (N), Ci activated Myr-Fu was retained in the cytoplasm (C).

(**G**) Adult wings of *fu* mutant flies in the absence or presence of transgenic expression of the indicated Fu constructs. Unlike Fu-WT, Myr-Fu failed to rescue the *fu* wing phenotype. Scale bars: 500 μm.

(**H**) Late third instar wing discs of $fu^{M1}$ mutants expressing Myc-tagged wild type (WT) or myristoylated (Myr) Fu were immunostained with the indicated antibodies. Scale bars are 50 μm. Unlike Fu-WT, Myr-Fu failed to rescue the A/P boundary expression of Ptc and En in *fu* wing discs.

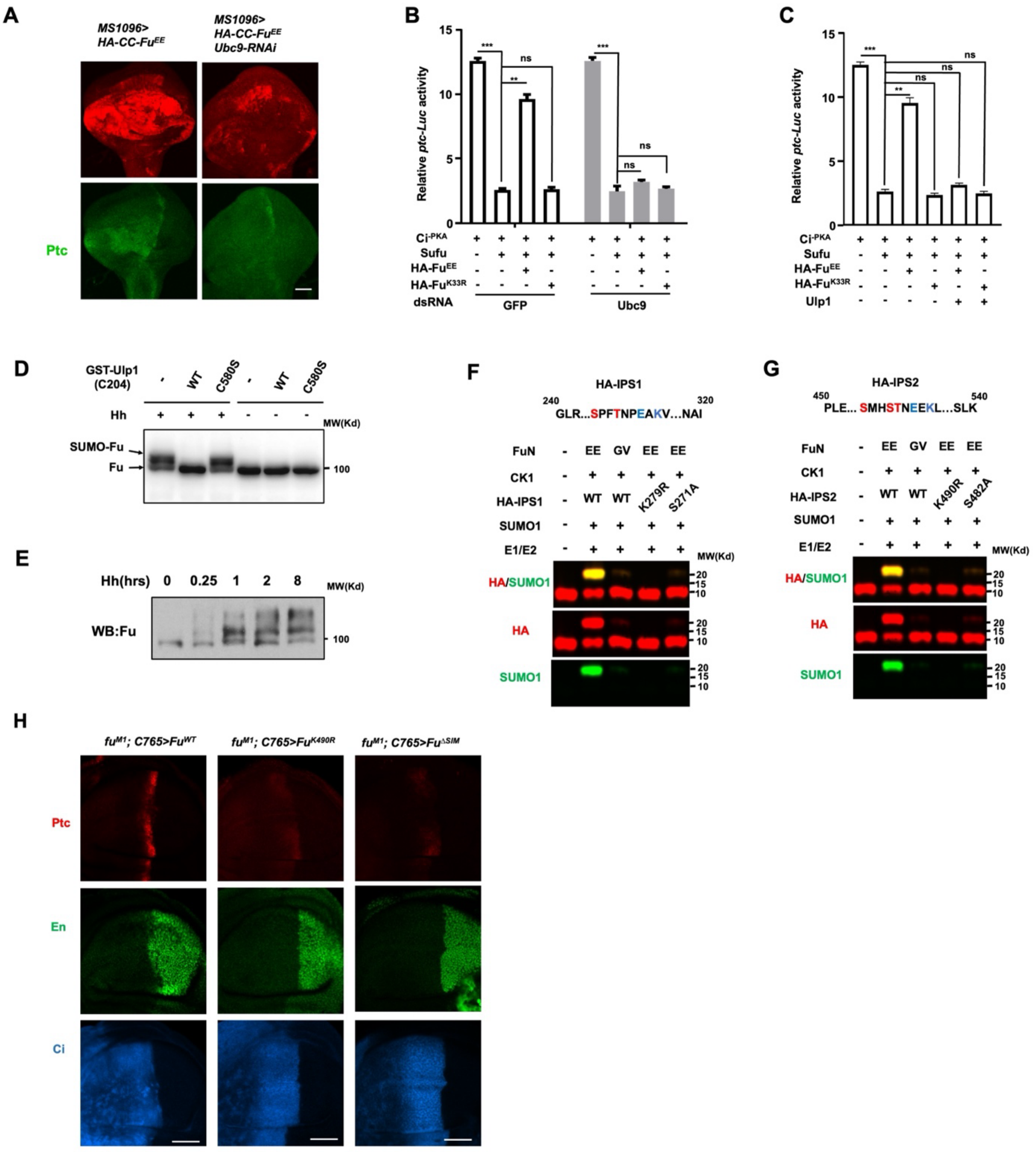


**Fig. S4. Characterization of phosphorylation-mediated Fu SUMOylation.**
(**A**) Late third instar wing discs expressing *HA-CC-Fu^EE^* with or without *Ubc9-RNAi* under the control of the *MS1096* Gal4 driver were immunostained with HA (red) and Ptc (green) antibodies. Depletion of Ubc9 downregulated *ptc* activation by HA-CC-Fu$^{EE}$ as well as the protein level of HA-CC-Fu$^{EE}$, suggesting that the SUMOylation pathway is required for the stability and activity of HA-CC-Fu$^{EE}$. Scale bar: 50 μm.

(**B**) *ptc-luc* reporter assay in S2R+ cells expressing the indicated constructs and treated with GFP or Ubc9 dsRNA. Depletion of Ubc9 downregulated the activity of HA-$Fu^{EE}$. Data are mean ± SD from three independent experiments. $^{**}P < 0.01$, $^{***}P < 0.001$ (*t* test). ns: not significant.
(**C**) *ptc-luc* reporter assay in S2R+ cells expressing the indicated constructs. Overexpression of the deSUMOylation enzyme Ulp1 downregulated the activity of HA-$Fu^{EE}$. Data are mean ± SD from three independent experiments. $^{**}P < 0.01$, $^{***}P < 0.001$ (*t* test). ns: not significant.
(**D**) *In vitro* deSUMOylation assays using Fu proteins immunopurified from Cl8 cells treated with Hh-conditioned or control medium as substrates and GST-fusion proteins containing the wild type (WT) or catalytic inactive (C580S) Ulp1 C-terminal 204 aa fragment (C204) as deSUMOylation enzymes.
(**E**) Western blot analysis of Fu from Cl8 cells treated with Hh-conditioned medium for the indicated time.
(**F**-**G**) *in vitro* SUMOylation assays of purified Fu fragment containing WT or mutant IPS1 (E) and IPS2 (F) and phosphorylated by the constitutively active (EE) or kinase dead (GV) FuN and CK1.
(**H**) Late third instar wing discs of $fu^{M1}$ mutants expressing the indicated Fu constructs were immunostained for Ptc, En and Ci. Both $Fu^{K490R}$ and $Fu^{\Delta SIM}$ failed to rescue Hh target gene expression in $fu^{M1}$ mutant discs. Scale bars are 50 μm.

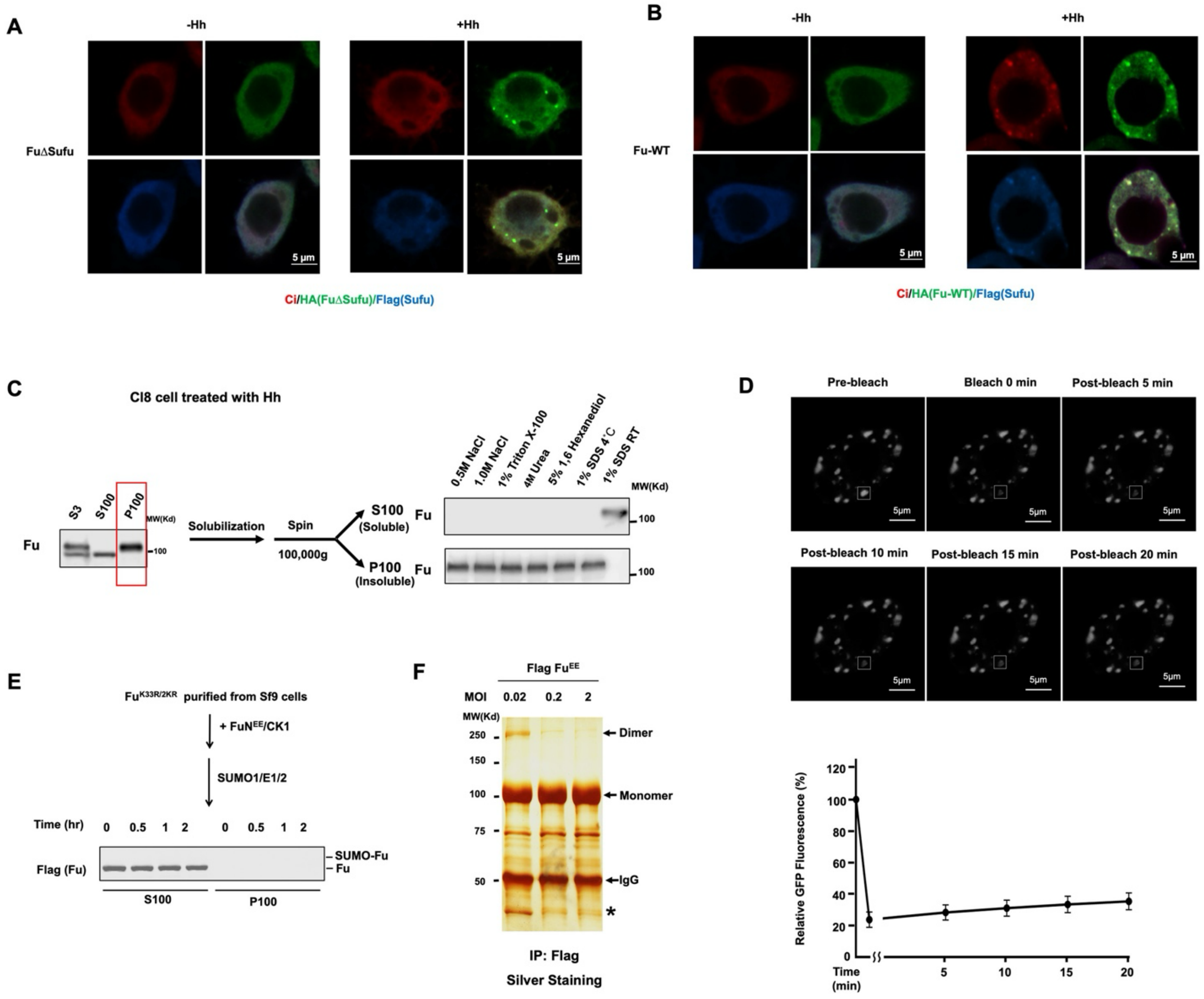


**Fig. S5. Characterization of Fu condensates.**

(**A**-**B**) Immunostaining for Ci, HA and Flag in Fu-depleted Cl8 cells stably expressing RNAi-insensitive HA-FuΔSufu (A) or HA-Fu-WT (B), transfected with Flag-Sufu, and treated with Hh-conditioned or control medium. FuΔSufu formed condensates in response to Hh stimulation without recruiting Ci and Sufu into the condensates (A) whereas Fu-WT formed condensates that also recruit Ci and Sufu in Cl8 cells treated with Hh (B).

(**C**) Fu condensates (P100) from Hh-treated Cl8 cells were solubilized with the indicated reagents and subjected to western blot analysis after centrifugation into S100 and P100 fractions.

(**D**) Representative Images of FRAP assay with mEGFP-Fu expressed in Cl8 cells treated with Hh for 16 hours. White rectangle indicates the area of photobleaching. Quantification of

fluorescence intensity of the photobleached area at the indicated time is shown in bottom panel. Data are shown as mean ± SD. n=5 puncta.

(**E**) *In vitro* condensation experiment using SUMOylation deficient (2KR) form of $Fu^{K33R}$ purified from Sf9 cells. Purified Flag-$Fu^{K33R2KR}$ was phosphorylated by $FuN^{EE}$/CK1 and then subjected to *in vitro* SUMOylation assay for the indicated time. Samples collected at indicated time points were separated into S100 and P100 fractions, followed by western blot analysis using anti-Flag antibody.

(**F**) Silver staining to reveal the maturation of Flag-$Fu^{EE}$ purified from Sf9 cells infected with increasing MOI (0.02, 0.2 and 2). Asterisk indicates a cellular protein with decreasing protein level.

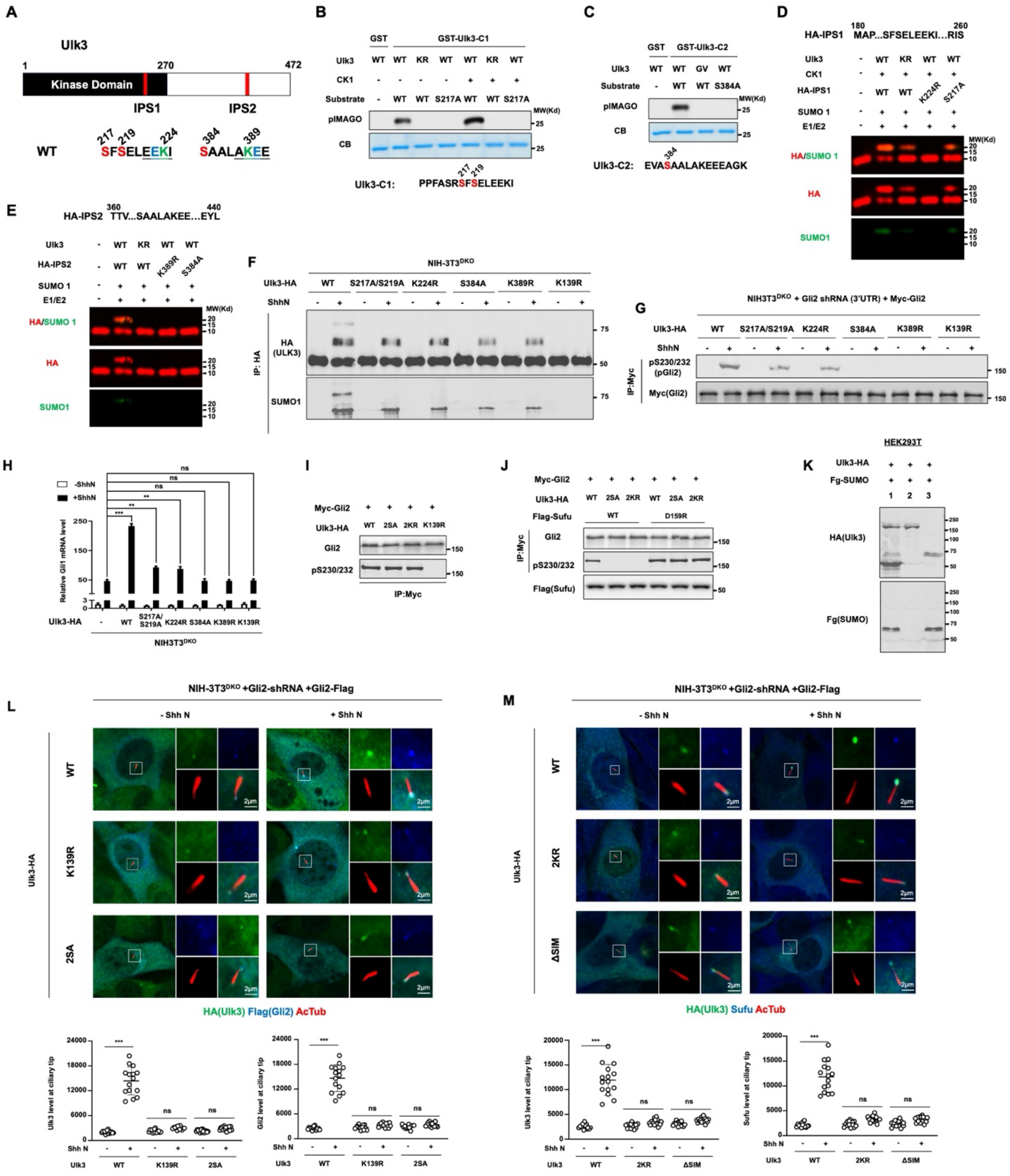

A
Ulk3
Kinase Domain
IPS1
IPS2
WT
SFSELEEKI
SAALAKEE
B
GST
GST-Ulk3-C1
pIMAGO
CB
Ulk3-C1: PPFASRSFSELEEKI
C
GST-Ulk3-C2
Ulk3-C2: EVASAALAKEEEAGK
D
HA-IPS1 MAP...SFSELEEKI...RIS
HA/SUMO 1
HA
SUMO1
E
HA-IPS2 TTV...SAALAKEE...EYL
F
NIH-3T3DKO
Ulk3-HA
IP: HA
HA (ULK3)
SUMO1
G
NIH3T3DKO + Gli2 shRNA (3'UTR) + Myc-Gli2
pS230/232 (pGli2)
Myc(Gli2)
IP:Myc
H
Relative Gli1 mRNA level
NIH3T3DKO
I
Myc-Gli2
Gli2
pS230/232
IP:Myc
J
Flag-Sufu
Flag(Sufu)
K
HEK293T
Fg-SUMO
HA(Ulk3)
Fg(SUMO)
L
NIH-3T3DKO +Gli2-shRNA +Gli2-Flag
- Shh N
+ Shh N
HA(Ulk3) Flag(Gli2) AcTub
Ulk3 level at ciliary tip
Gli2 level at ciliary tip
M
HA(Ulk3) Sufu AcTub
Sufu level at ciliary tip

**Fig. S6. Phosphorylation-dependent SUMOylation regulates Ulk3 ciliary tip localization and activity.**

(**A**) Schematic drawing of Ulk3 protein with the two Inverted Phosphorylation dependent SUMOylation motifs (IPS1 and IPS2) indicated. Fu sites and SUMOylation sites are highlighted in red and green, respectively

(**B**-**C**) *In vitro* kinase assay using wild type (WT), or kinase dead (KR) Ulk3 purified from HEK293T cells and recombinant CK1 as kinases and the indicated GST-fusion proteins containing Ulk3 C1 (B) or C2 (C) fragment as substrates. Phosphorylation was detected by the pIMAGO system.

(**D**-**E**) *in vitro* SUMOylation assay of Ulk3-HA fragment containing IPS1 (D) and IPS2 (E) immunopurified from HEK293T cells and phosphorylated by purified $Ulk3^{WT}$ or $Ulk3^{KR}$ with (D) or without (E) recombinant CK1. Recombinant SUMO1, E1 and E2 were added in the SUMOylation assay.

(**F**) Western blot analysis of Ulk3 (wild type and indicated mutants) and its SUMOylation expressed in $NIH3T3^{DKO}$ cells treated with or without Shh.

(**G**) Western blot analysis of Myc-Gli2 phosphorylation in $NIH3T3^{DKO}$ cells expressing either wild type or the indicated mutant Ulk3 and with endogenous Gli2 depleted by shRNA and treated with or without Shh.

(**H**) qRT-PCR analysis of *Gli1* expression in $NIH3T3^{DKO}$ cells expressing either wild type or the indicated mutant Ulk3 and treated with or without Shh. Data are mean ± SD from three independent experiments. $^{**}P < 0.01$, $^{***}P < 0.001$ (*t* test). ns: not significant.

(**I**) Western blot analysis of Myc-Gli2 phosphorylation in HEK293T cells co-expressing Myc-Gli2 and either wild type or the indicated mutant Ulk3.

(**J**) Western blot analysis of Myc-Gli2 phosphorylation in HEK293T cells co-expressing Ulk3 (either wild type or the indicated mutants) and Flag-Sufu (either wild type or the D159R mutant).

(**K**) Western blot analysis of SUMOylation of Ulk3-HA purified from HEK293T cells coexpressing Ulk3-HA and Flag (Fg)-SUMO1. Ulk3-HA was immunoprecipitated with HA antibody and subjected to western blot analysis with the indicated antibodies (Lane 1). Eluted protein was separated by 6% SDS PAGE and mature Ulk3 was cut from gel blindly. The gel slices were further cut into small pieces and electro-eluted in Tris Glycine Buffer with 0.01% SDS at 4 degree for 10 hours. The resultant elute was concentrated by ultrafiltration (Lane 2) and incubated in 2X SDS loading buffer at -18 degree for 8 days (Lane 3), followed by western blot analysis with the indicated antibodies.

(**L**-**M**) Immunostaining (top) and quantification (bottom; n=20 cells) of ciliary localized of Ulk3-HA and Flag-Gli2 (L) or Sufu (M) in Ulk3/Stk36 DKO and Gli2 depleted NIH3T3 cells expressing a Flag-tagged Gli2 construct and the indicated Ulk3-HA constructs and treated with or without Shh. Data are mean ± SD. $^{*}P < 0.05$, $^{**}P < 0.01$(*t* test). ns: not significant.

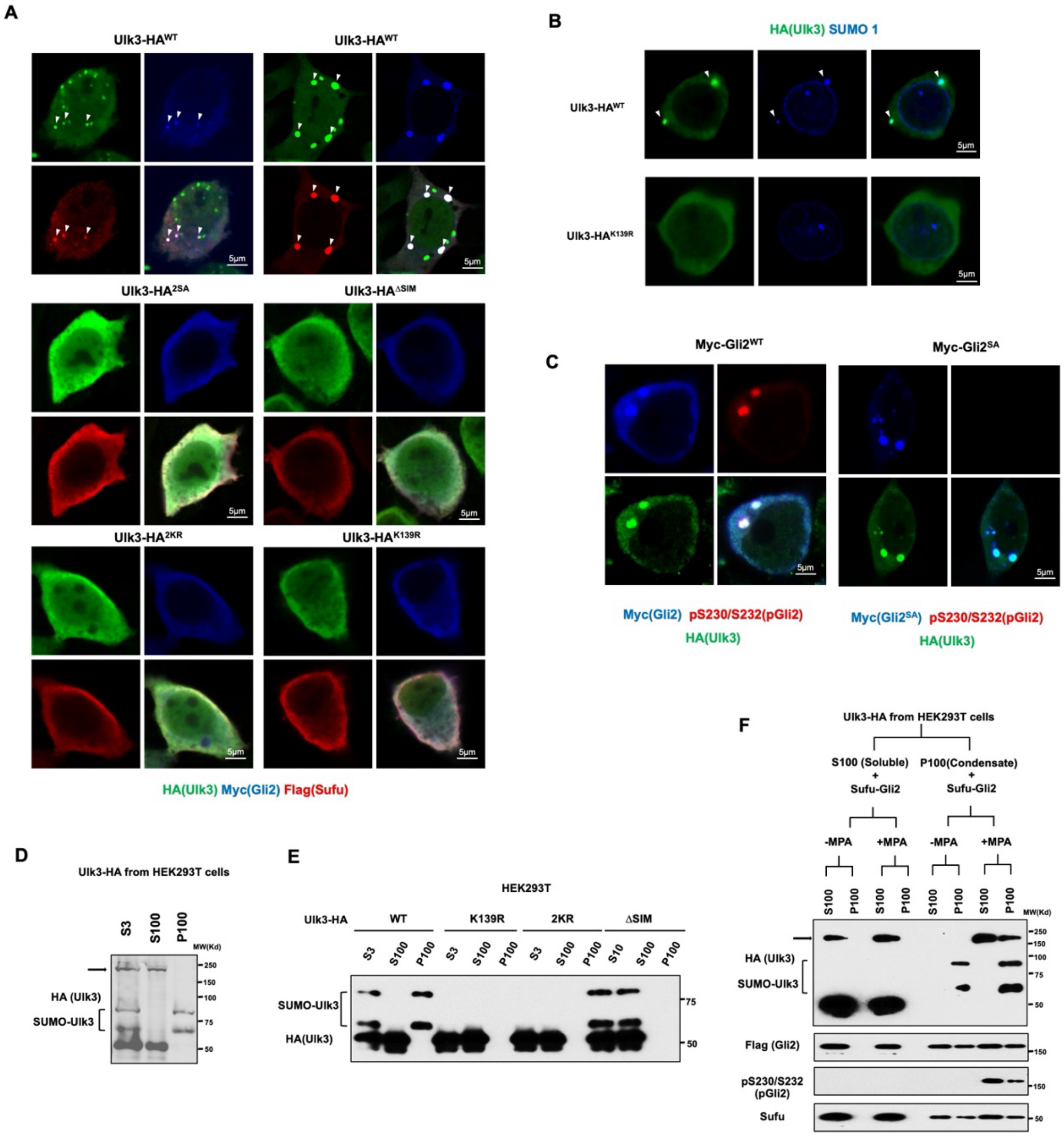


**Fig. S7. Characterization of Ulk3 condensates in HEK293T cells.**

(**A**) Representative images of immunostained HEK293T cells transfected with the indicated Ulk3-HA expression constructs together with Myc-Gli2 and Flag-Sufu expressed constructs. Arrowheads indicate Ulk3 puncta. Ulk3-HA$^{WT}$ but not Ulk3-HA$^{2SA}$ or HA-Ulk3-HA$^{\Delta SIM}$ formed condensates that recruit Myc-Gli2 and Flag-Sufu.

(**B**) Immunostaining to visualize HA (green) and SUMO1 (blue) signals in HEK293T cells transfected with the indicated Ulk3-HA constructs. Arrowheads indicate Ulk3 puncta. SUMO1 signal is enriched in Ulk3-HA condensates.

(**C**) Immunostaining to visualize Myc (blue), pGli2 (red) and HA (green) signals in HEK293T cells transfected with Ulk3-HA, Flag-Sufu, and the indicated Myc-Gli2 constructs. pGli2 could be detected in Ulk3 condensates.
(**D**) Western blot analysis of exogenously expressed wild type Ulk3-HA in S3, S100 and P100 fractions from HEK293T cells. Ulk3-HA in S3 and S100 fractions was immunoprecipitated with anti-HA antibody prior to western blot analysis. Arrow indicates the mature Ulk3, which was largely present in the soluble fractions. Bracket indicates SUMOylated Ulk3.
(**E**) Western blot analysis of exogenously expressed HA-tagged wild type (WT) Ulk3 and the indicated mutant Ulk3 in S3, S100 and P100 fractions from HEK293T cells. Ulk3-HA$^{K139R}$, Ulk3-HA$^{2KR}$ and Ulk3-HA$^{\Delta SIM}$ failed to form condensates (P100). Bracket indicates SUMOylated Ulk3.
(**F**) *In vitro* Ulk3 maturation and Gli2 phosphorylation. Ulk3-HA protein expressed in HEK293T cells was separated into S100 and P100 fractions and then incubated with or without MPA (HEK293T cell lysate, ATP and SUMO1) in the presence of Gli2-Sufu complexes purified from HEK293T cells for 6 hours, followed by centrifugation into S100 and P100 fractions and immunoblot analysis with the indicated antibodies. Arrow indicates the mature Ulk3 and Bracket indicates SUMOylated Ulk3.

# Uncropped Western Blots

**Fig 1B**

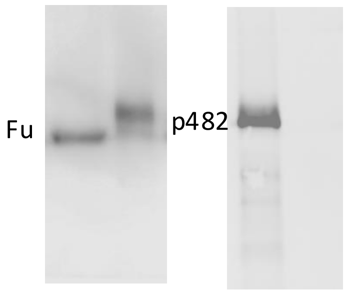


**Fig 1C**

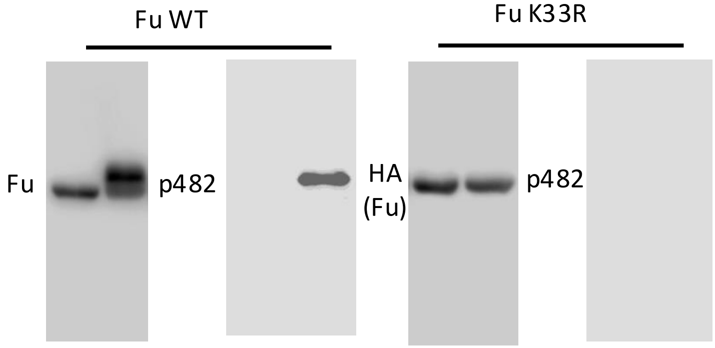


**Fig 1K**

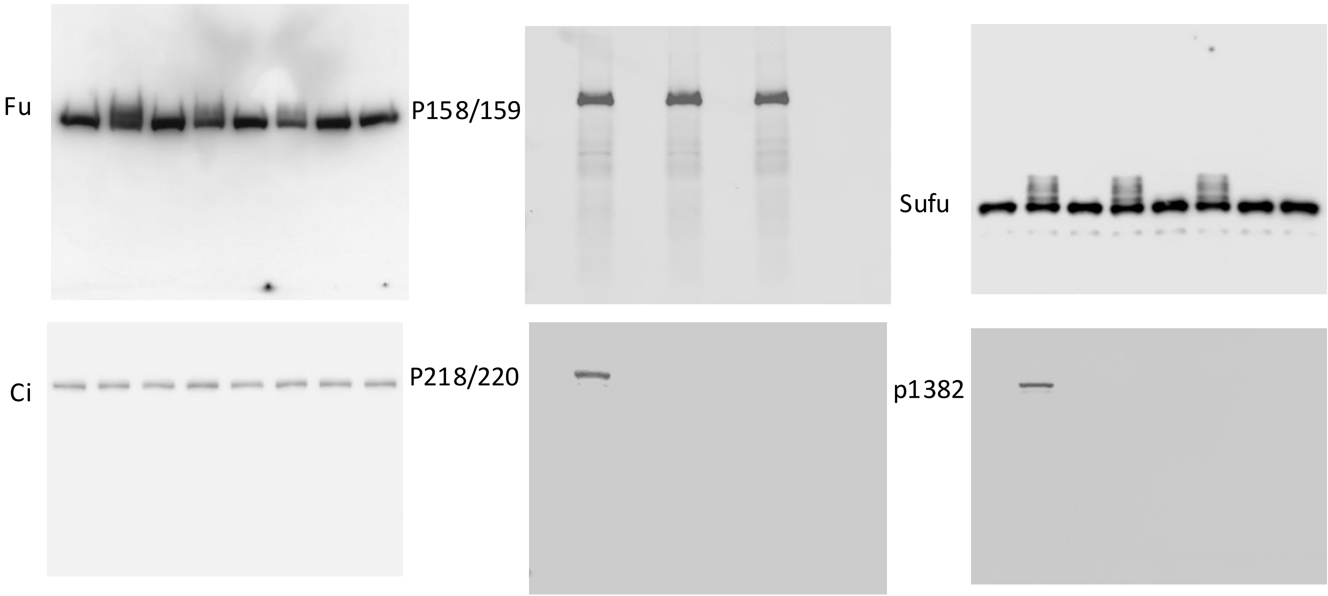


**Fig 1L**

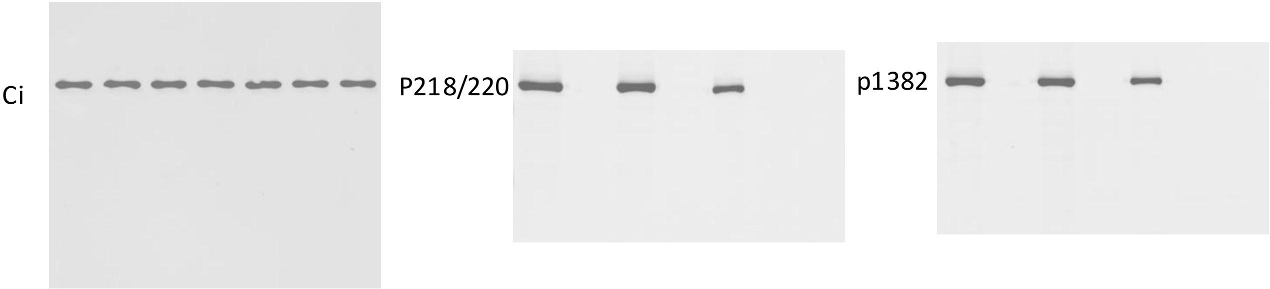


**Fig 1M**

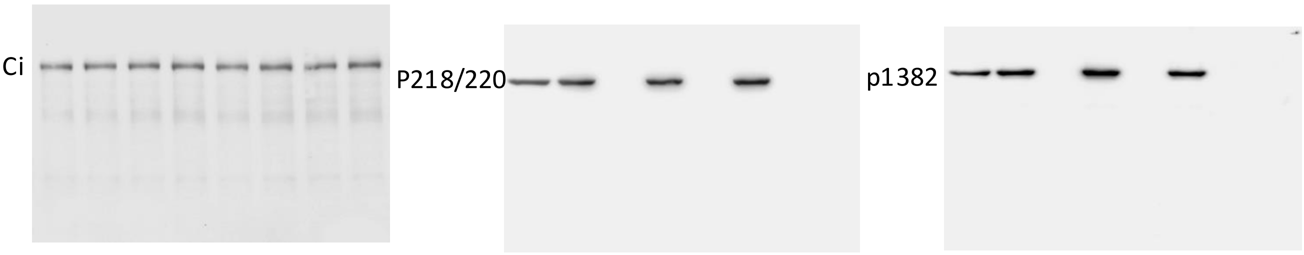

## Fig 1N

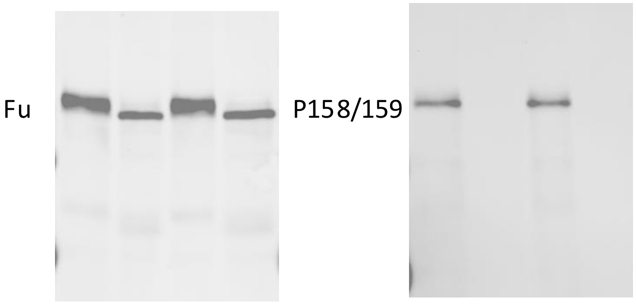


## Fig 1O

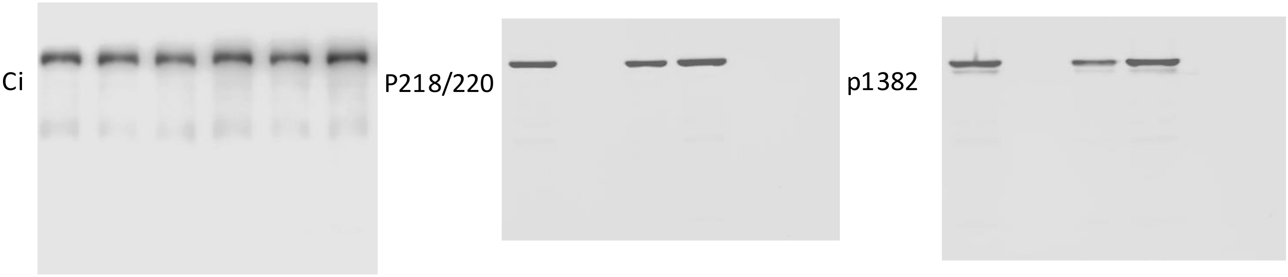


## Fig 1P

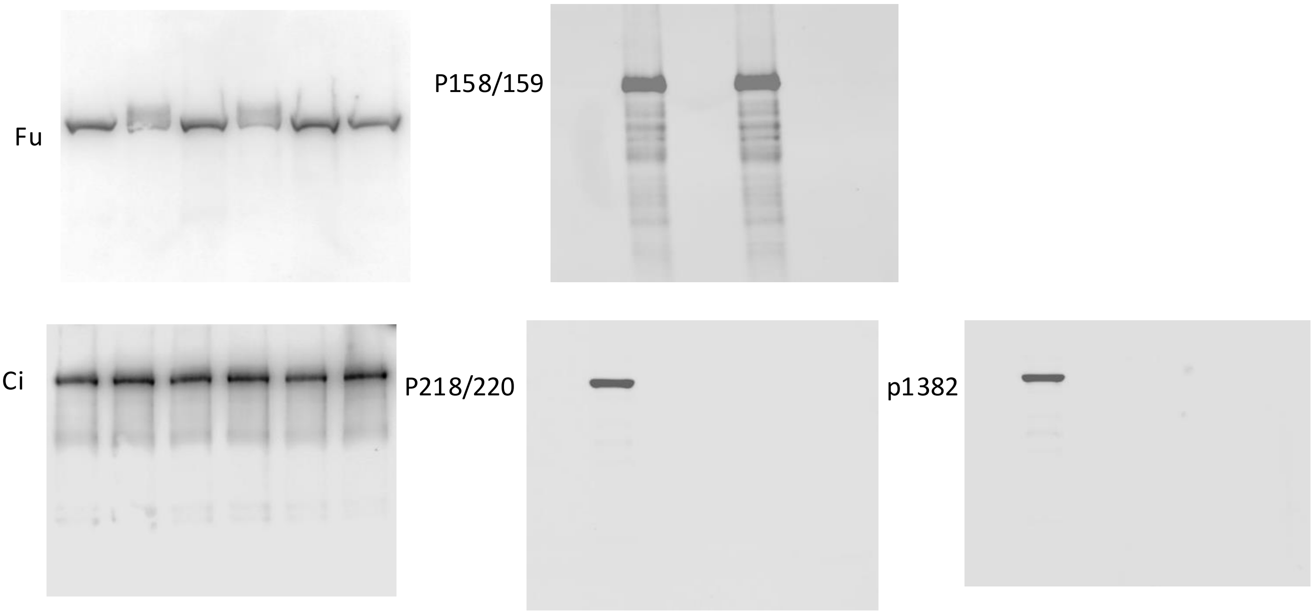

**Fig 2A**

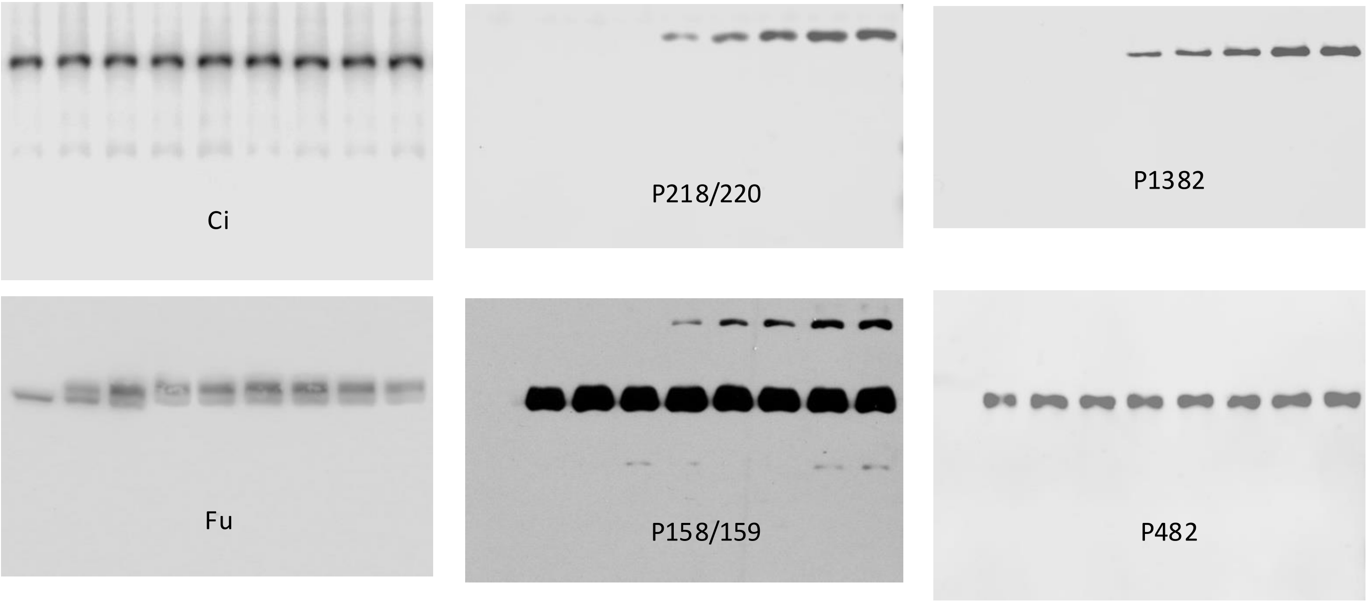


**Fig 2B**

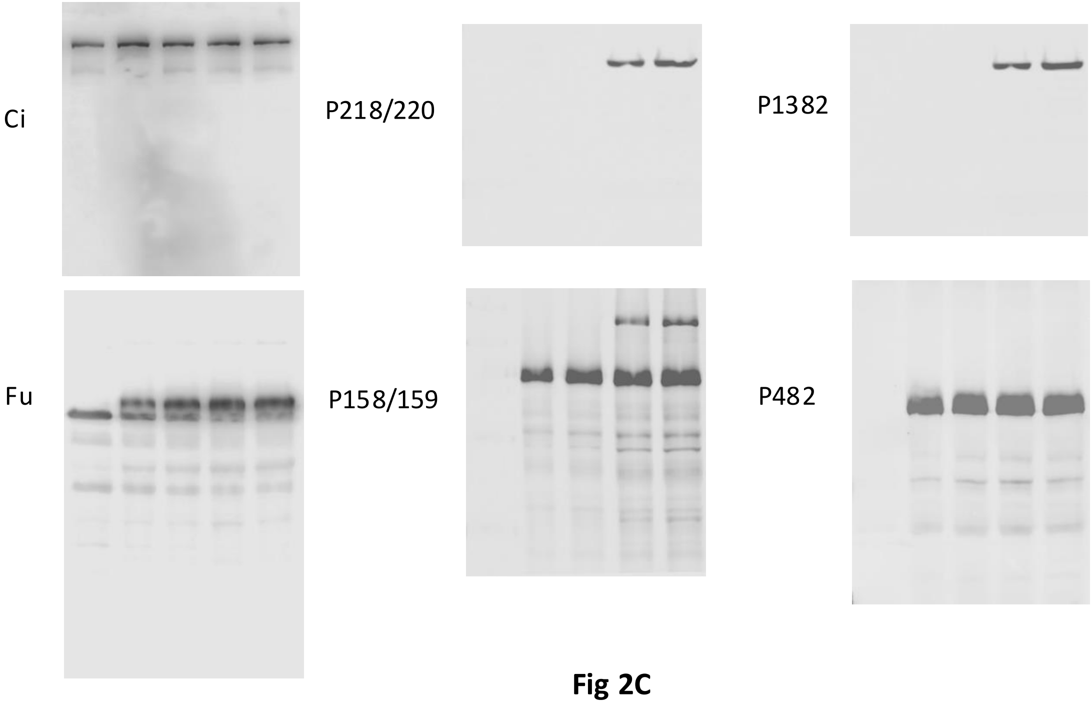


**Fig 2C**

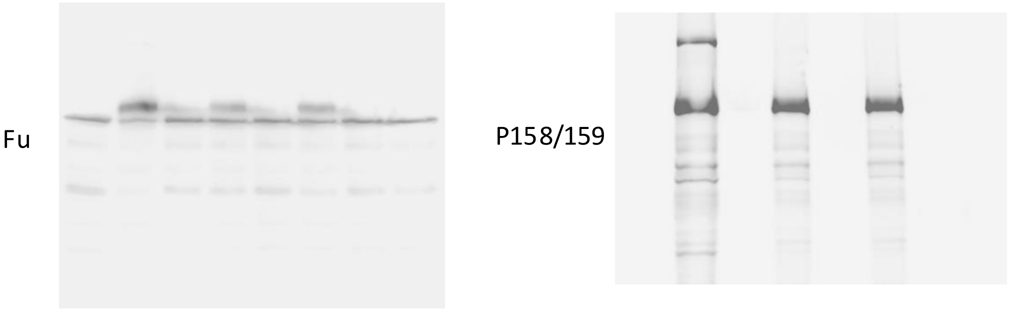

**Fig 2D**

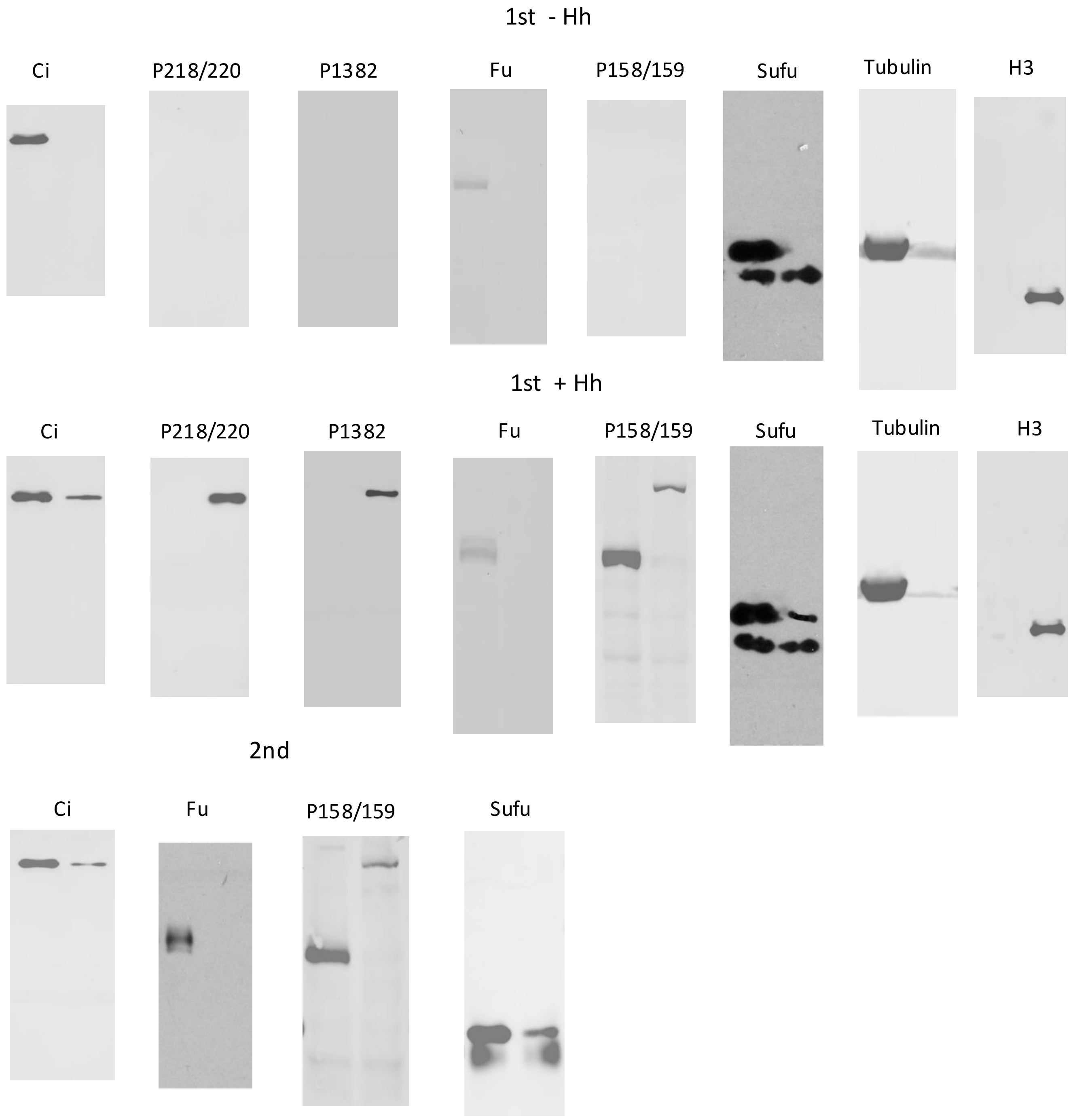
1st - Hh
Ci
P218/220
P1382
Fu
P158/159
Sufu
Tubulin
H3
1st + Hh
Ci
P218/220
P1382
Fu
P158/159
Sufu
Tubulin
H3
2nd
Ci
Fu
P158/159
Sufu

**Fig 2E**

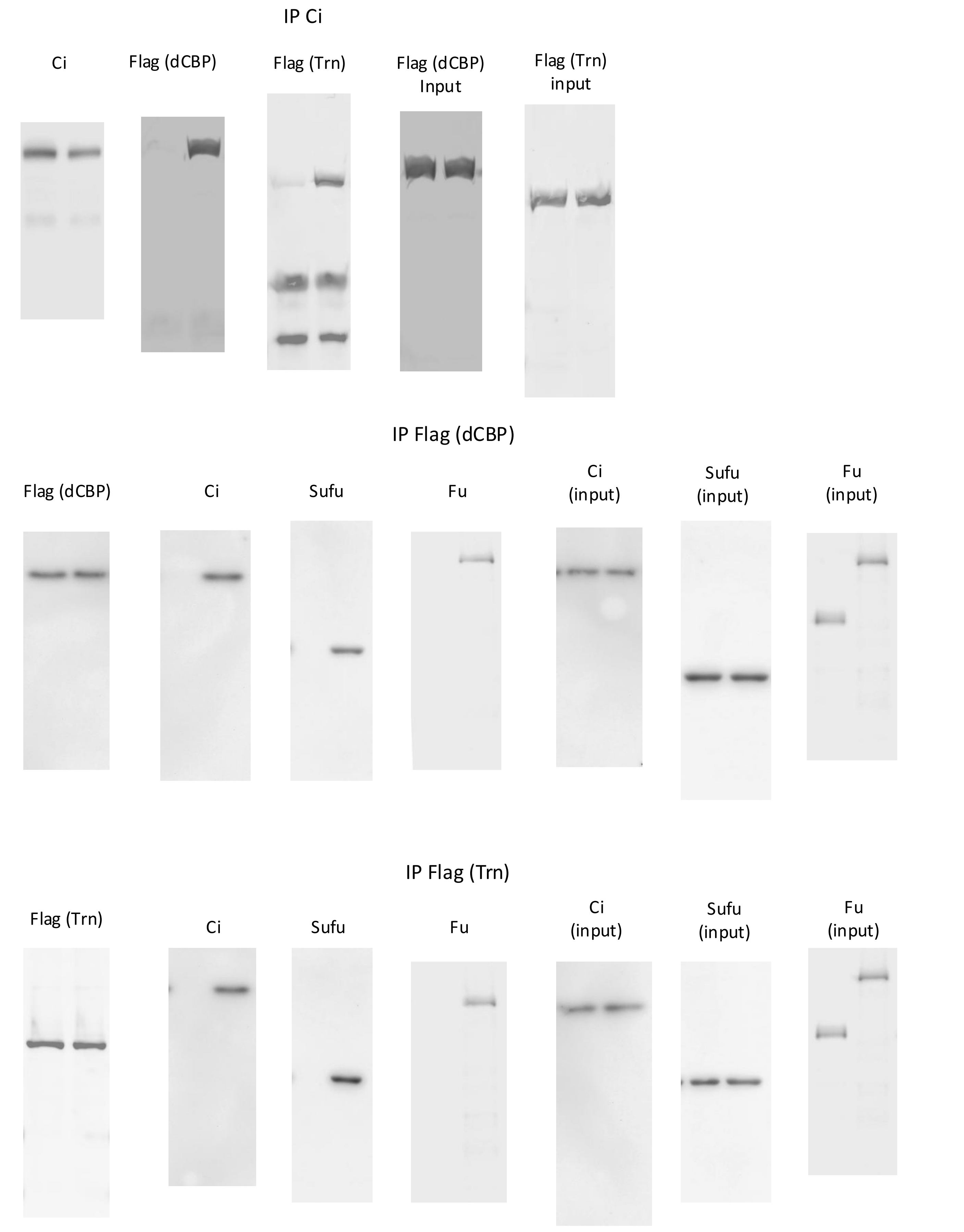
IP Ci
Ci
Flag (dCBP)
Flag (Trn)
Flag (dCBP) Input
Flag (Trn) input
IP Flag (dCBP)
Flag (dCBP)
Ci
Sufu
Fu
Ci (input)
Sufu (input)
Fu (input)
IP Flag (Trn)
Flag (Trn)
Ci
Sufu
Fu
Ci (input)
Sufu (input)
Fu (input)

## Fig 3B

HA (Fu)

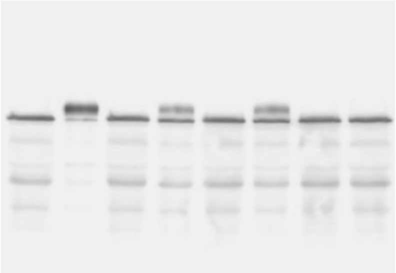

Flag (SUMO)

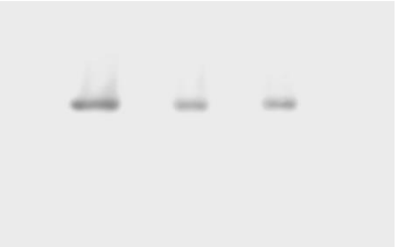

## Fig 3C

HA (Fu)

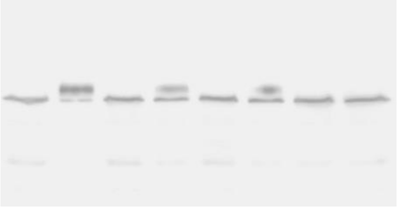

Flag (SUMO)

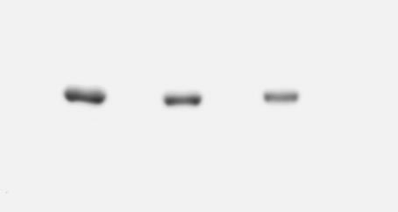

## Fig 3D

HA (Fu)

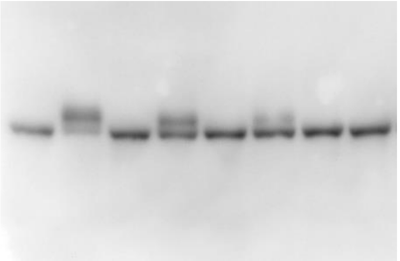

P158/159

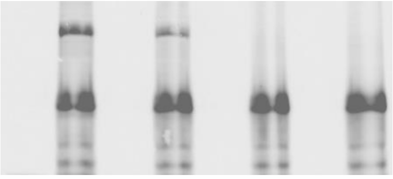

Ci

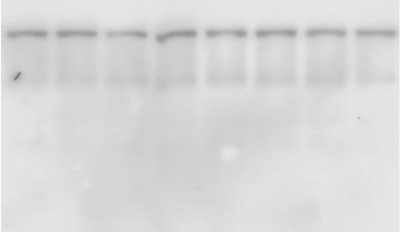

P218/220

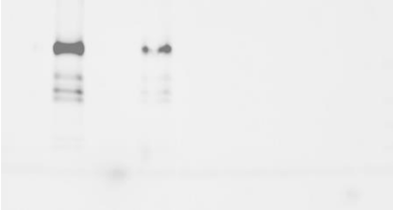

P1382

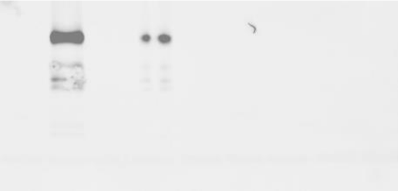

**Fig 3E**

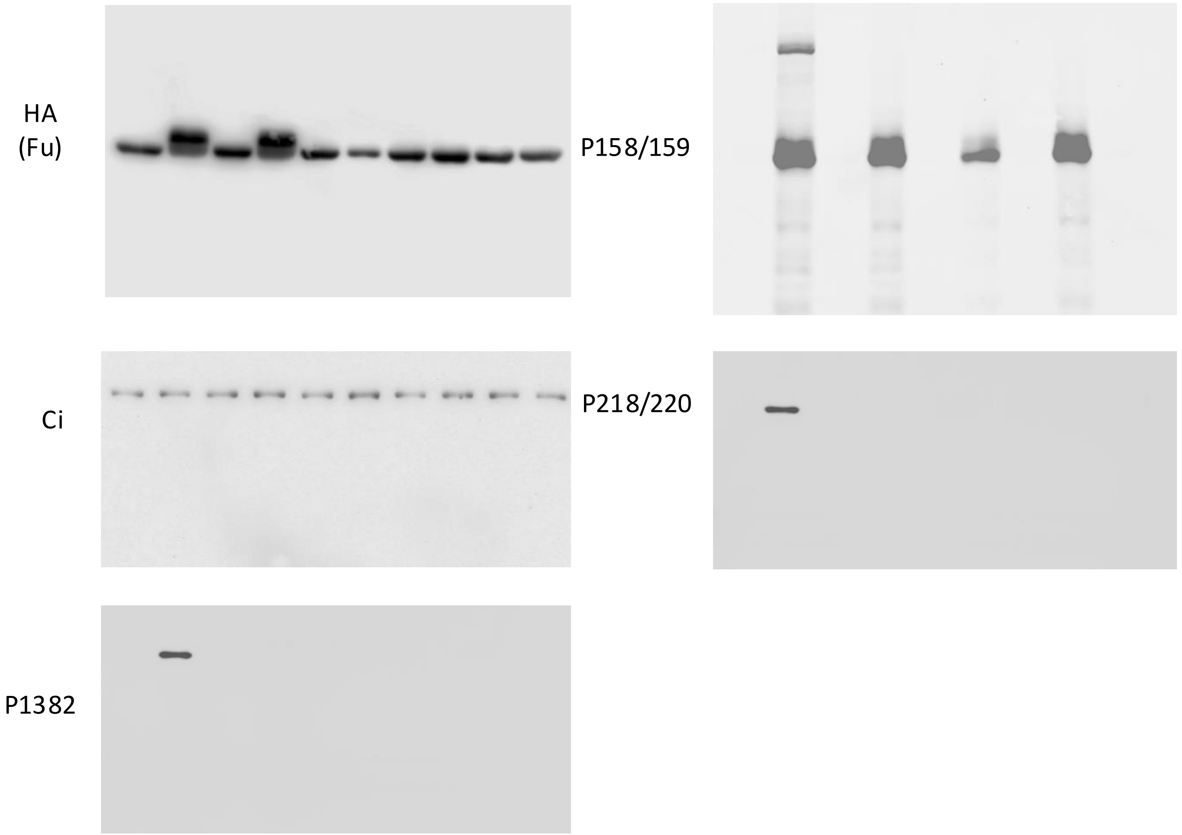


**Fig 3F**

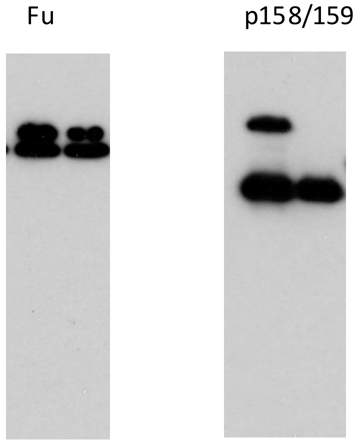

**Fig 4D**

WT

HA(Fu) p158/159 Sufu Ci p218/220

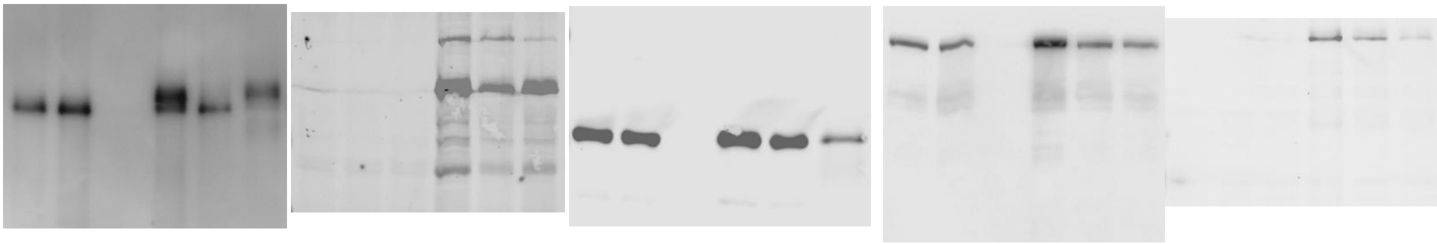

K33R

HA(Fu) p158/159 Sufu Ci p218/220

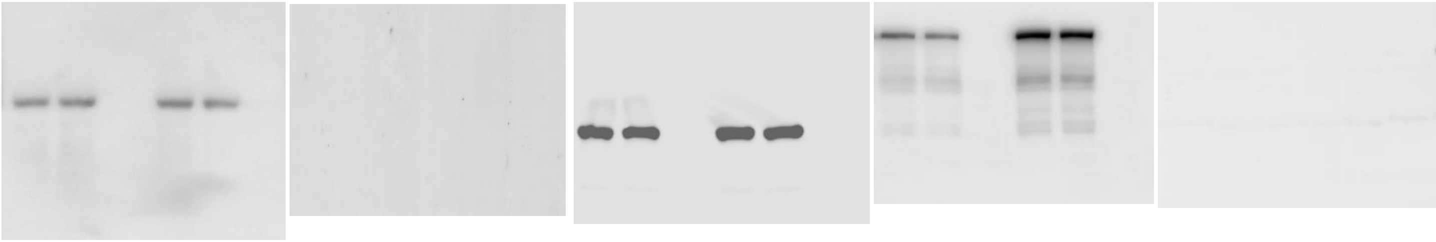

2KR

HA(Fu) p158/159 Sufu Ci p218/220

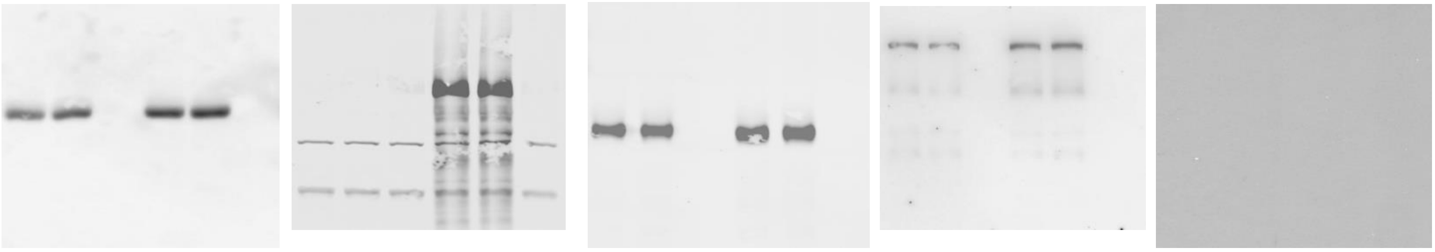

AV/AAV

HA(Fu) p158/159 Sufu Ci p218/220

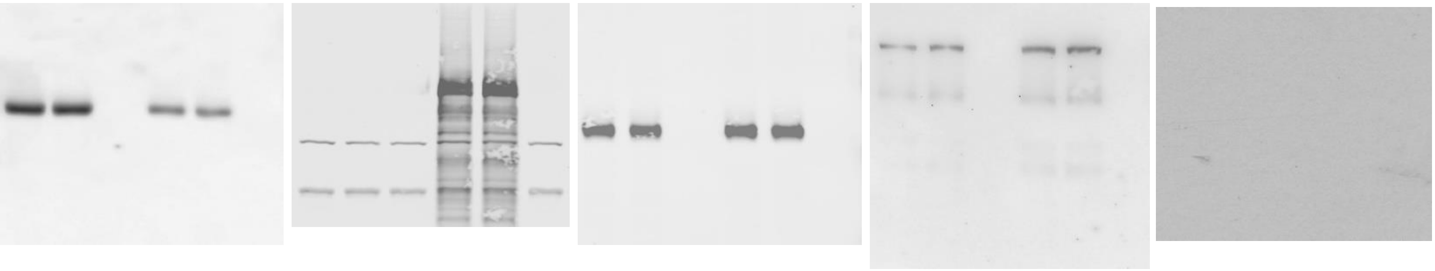

ΔSim

Fu p158/159 Sufu Ci 218/220

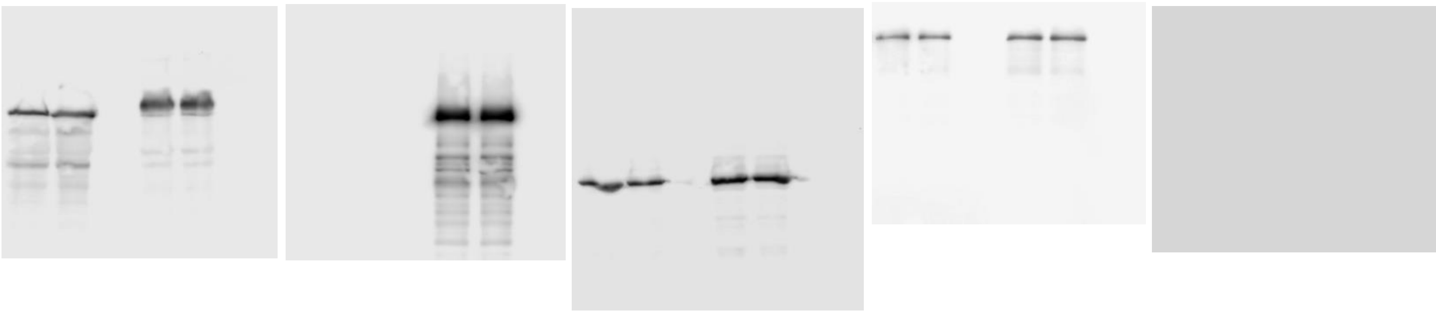

**Fig 4E**

-SUMO1

Flag (Fu)

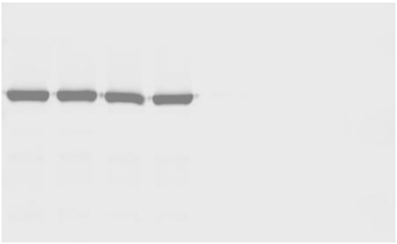

+SUMO1

Flag (Fu)

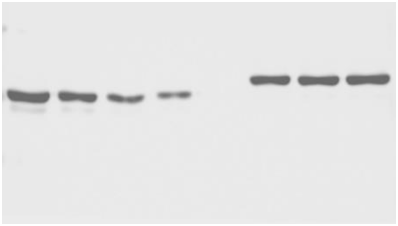

**Fig 4F**

Ulp1 WT

Flag (Fu)

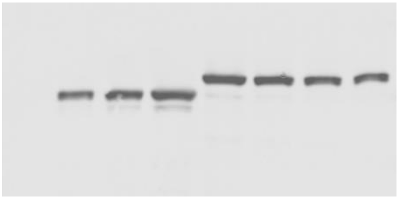

Ulp1 C580S

Flag (Fu)

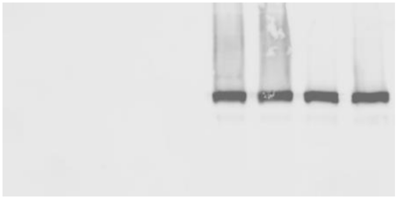

**Fig 5A**

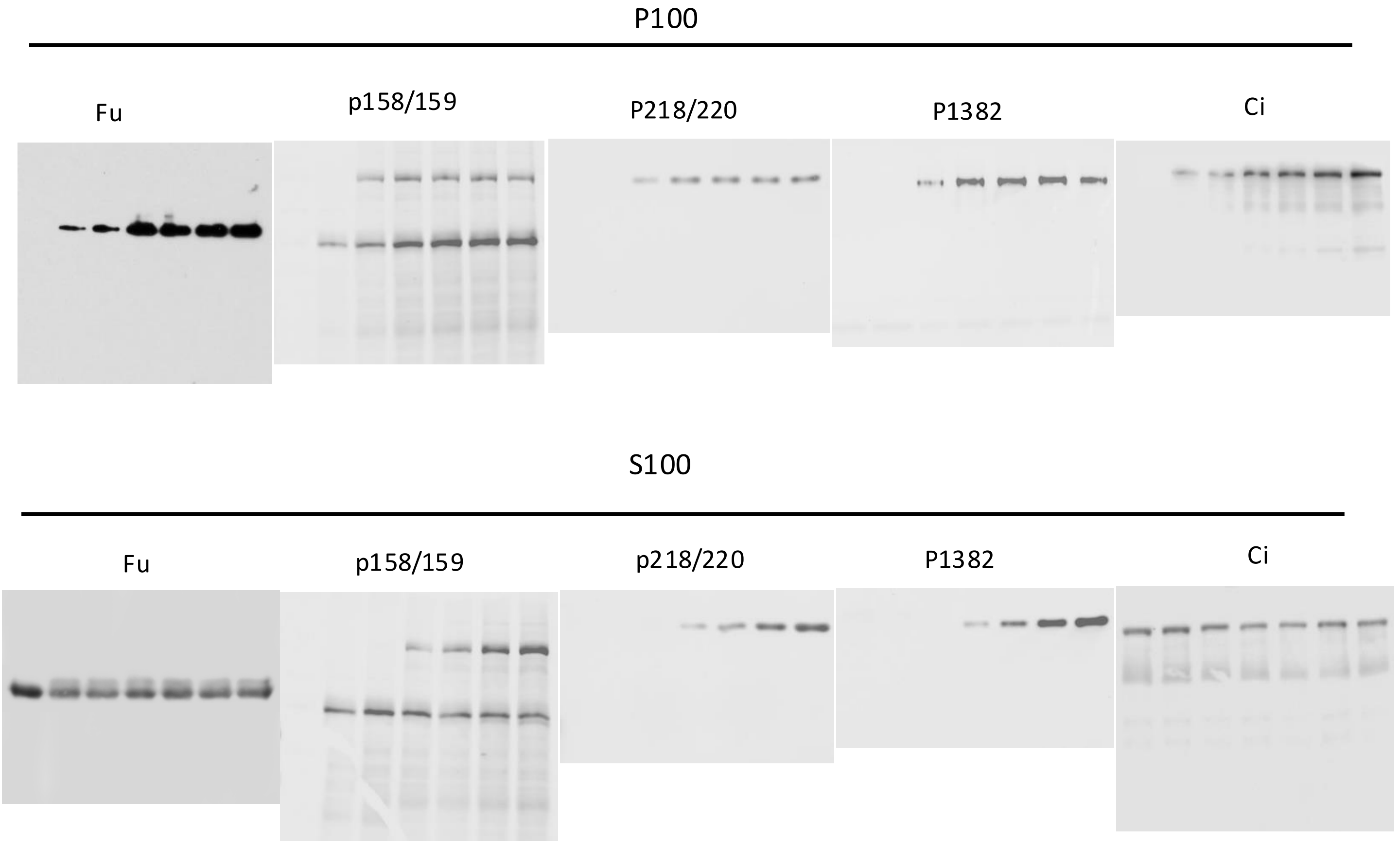


**Fig 5B**

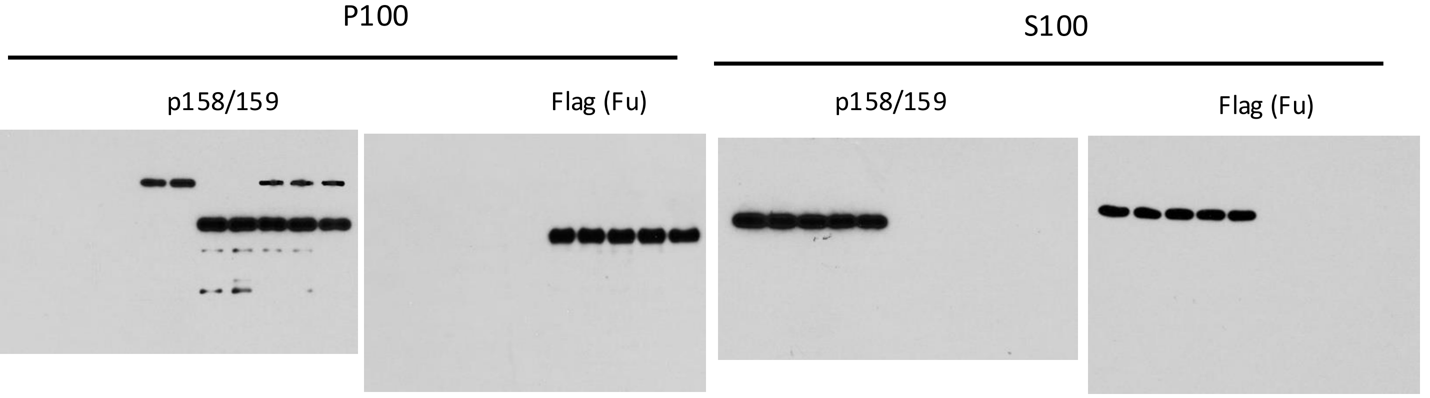


**Fig 5C**

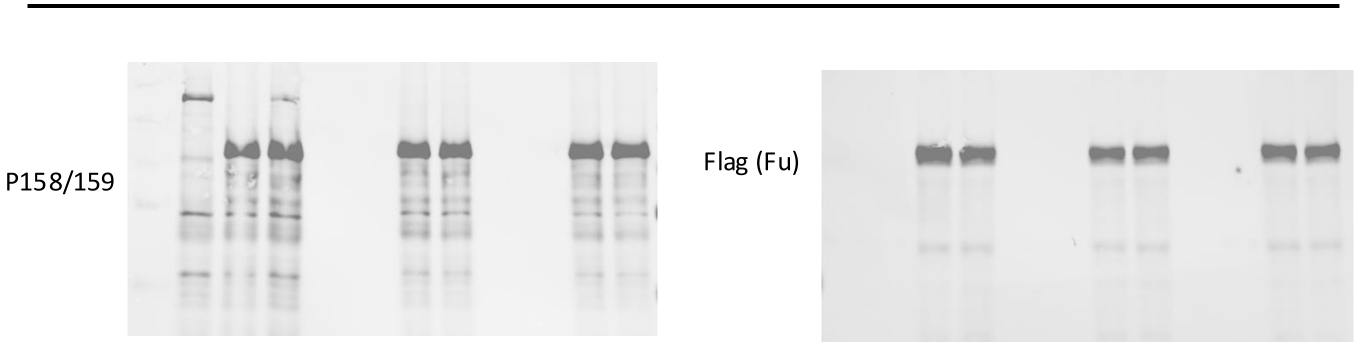

## Fig 5D

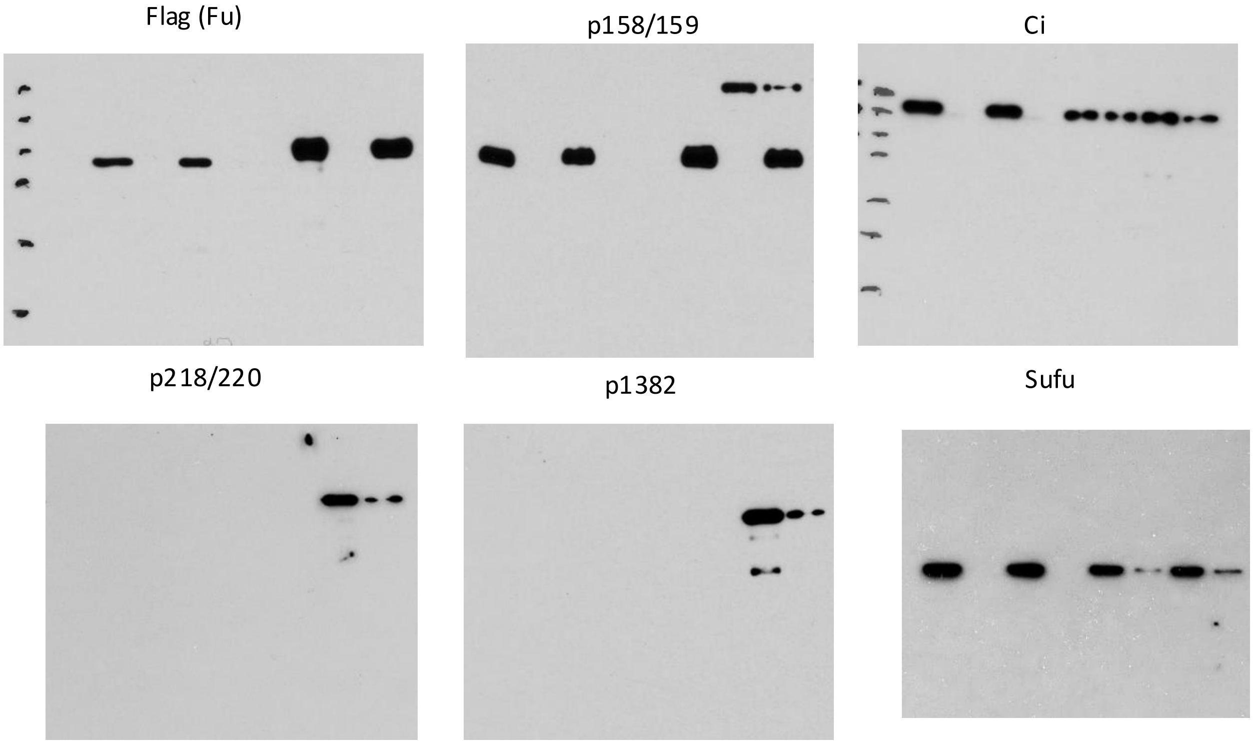


## Fig 5E

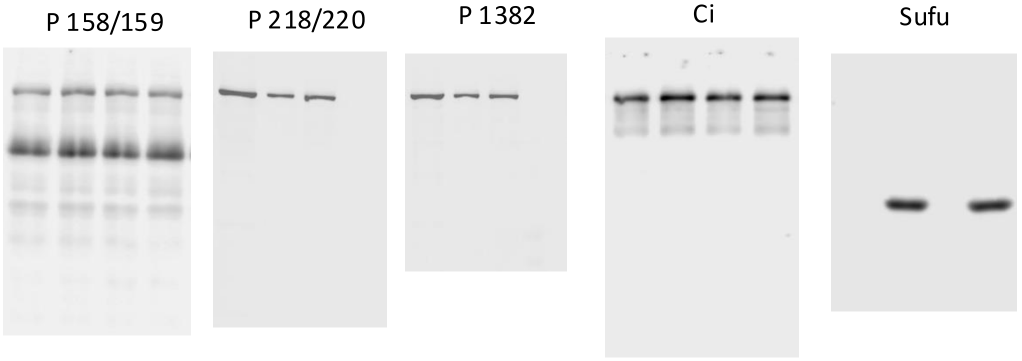


## Fig 5F

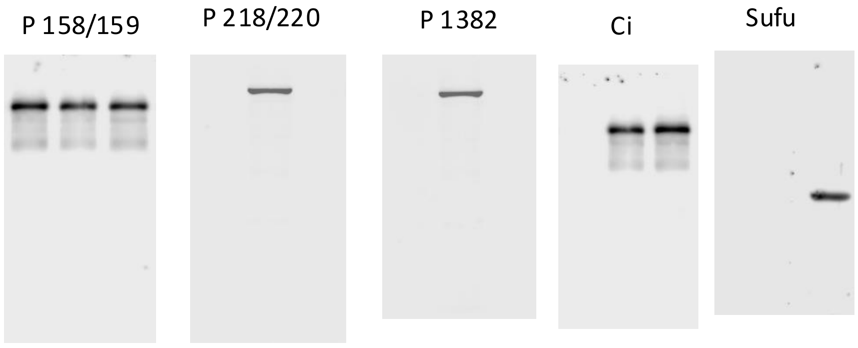

**Fig 6C**

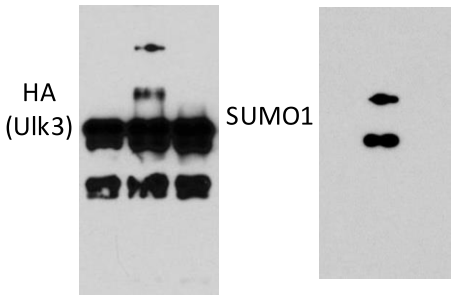


**Fig 6D**

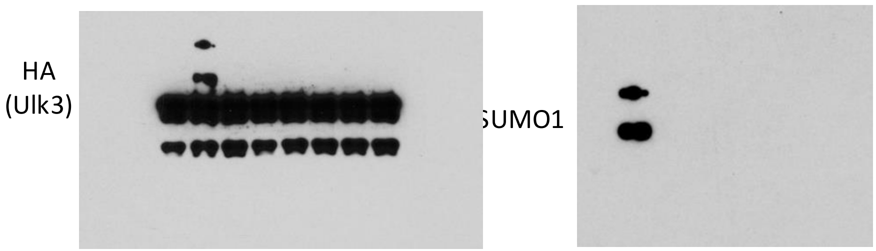


**Fig 6F**

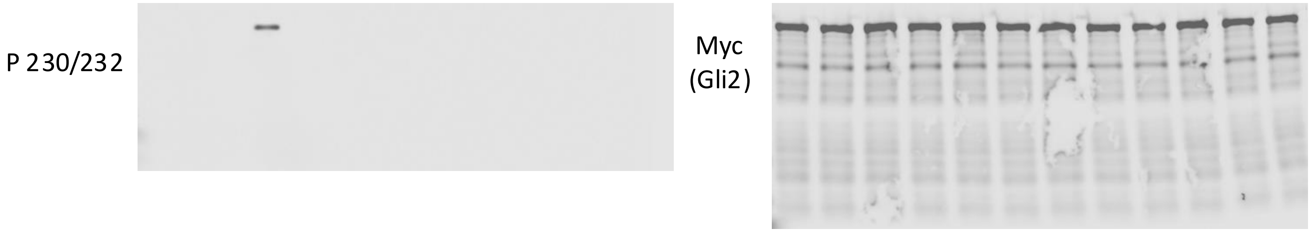


**Fig 6G**

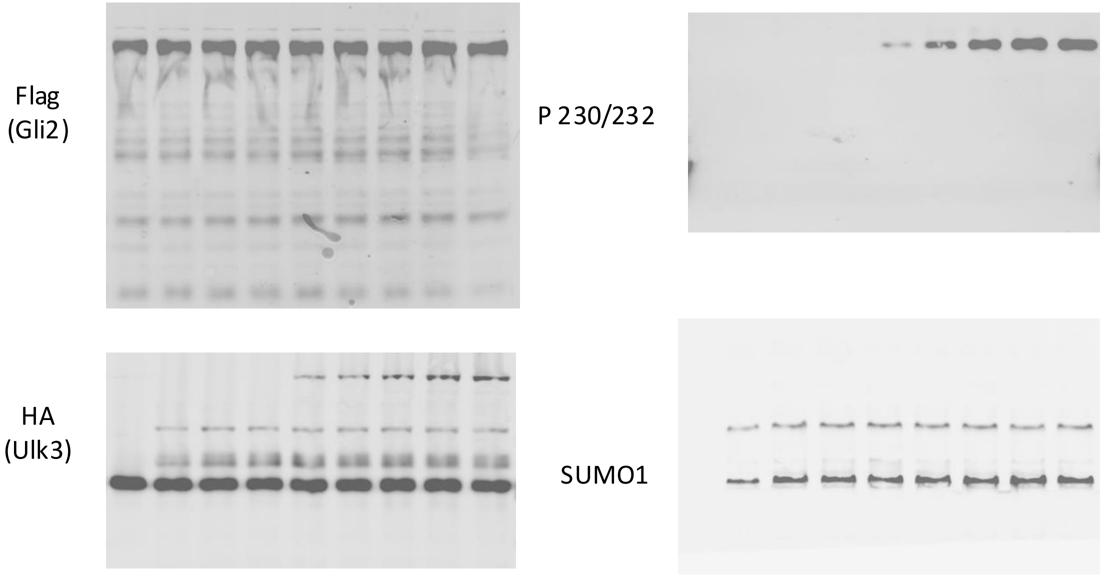


**Fig 6H**

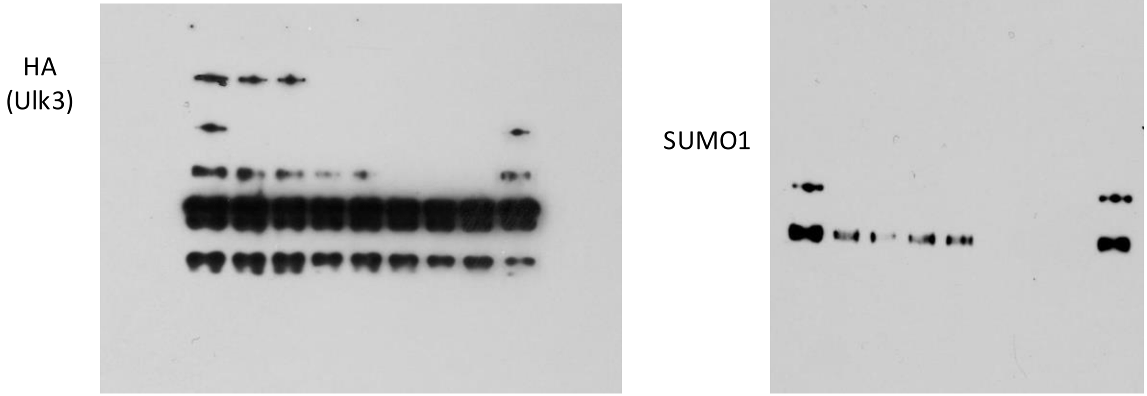

**Fig 6I**

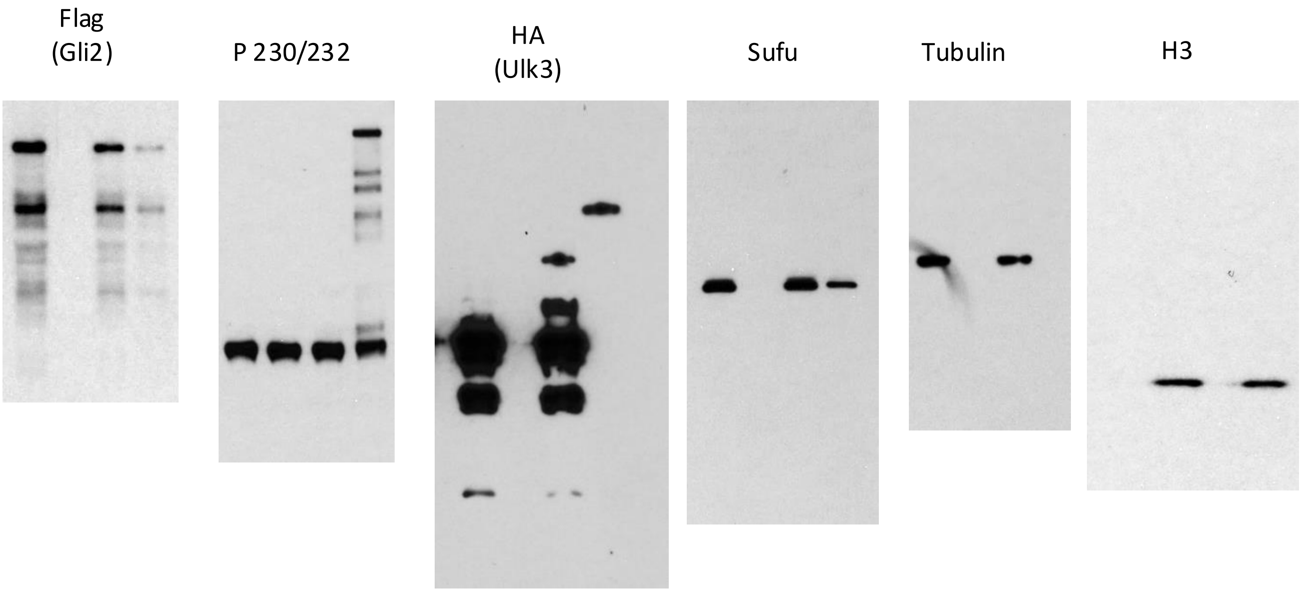

**Fig 7G**

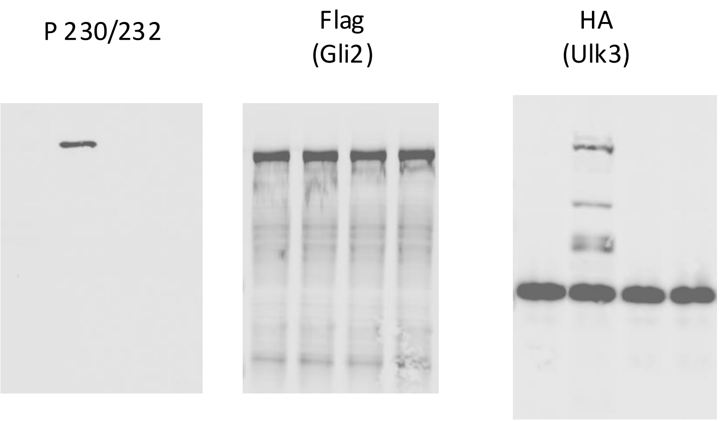

**Fig S1B**

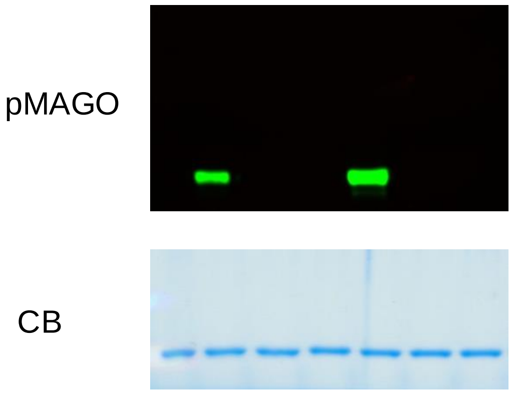


**Fig S1C**

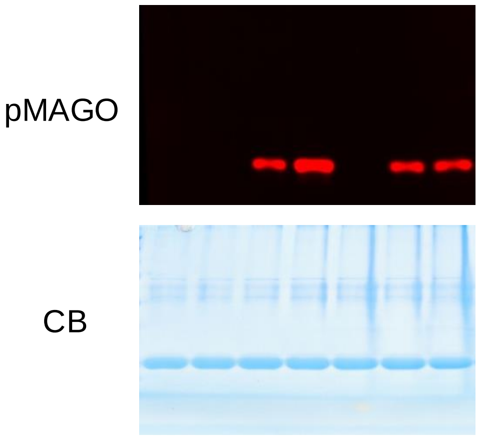


**Fig S1D**

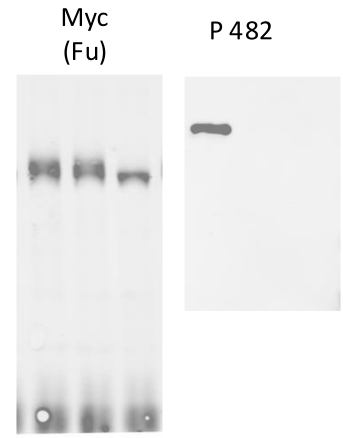


**Fig S1G**

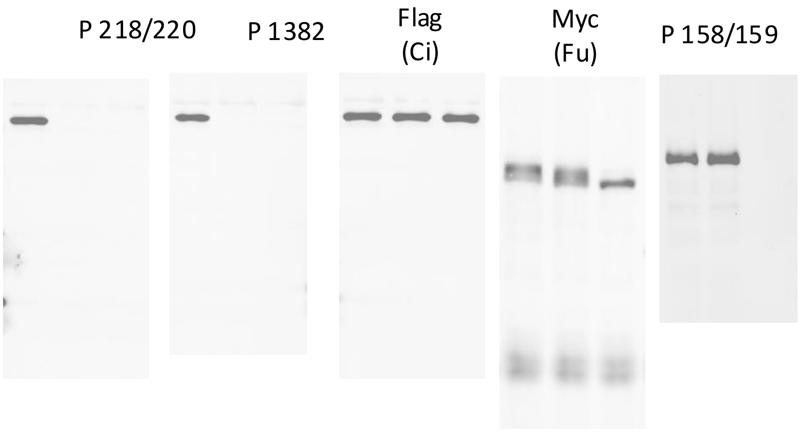


**Fig S1H**

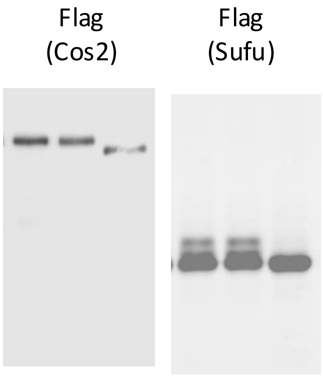


**Fig S1I**

**Fig S1I**

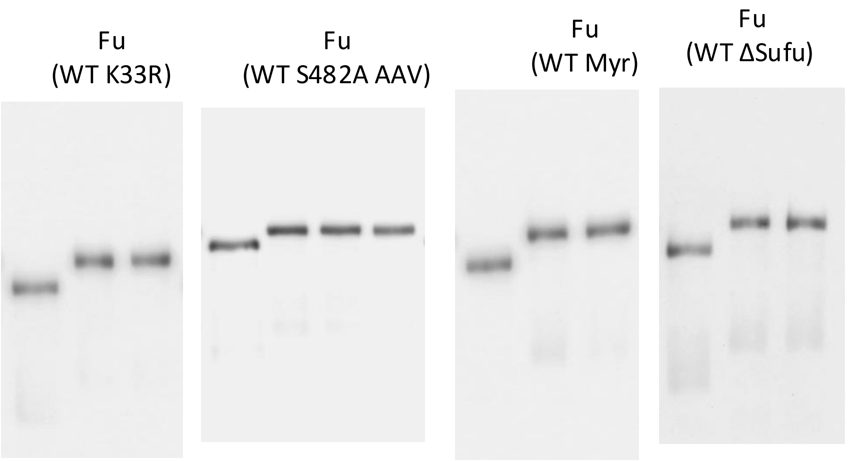

**Fig S1I**

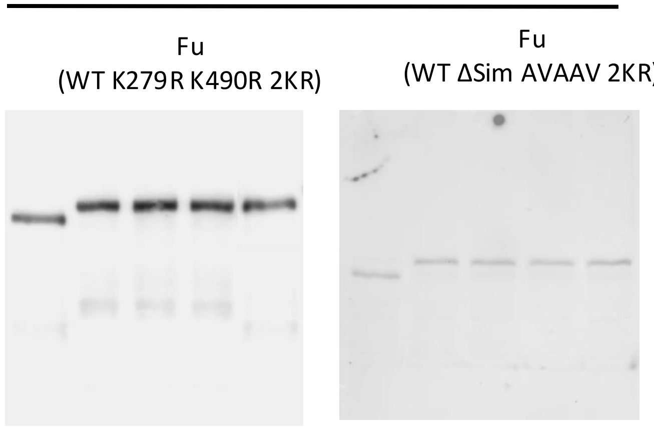


**Fig S2D**

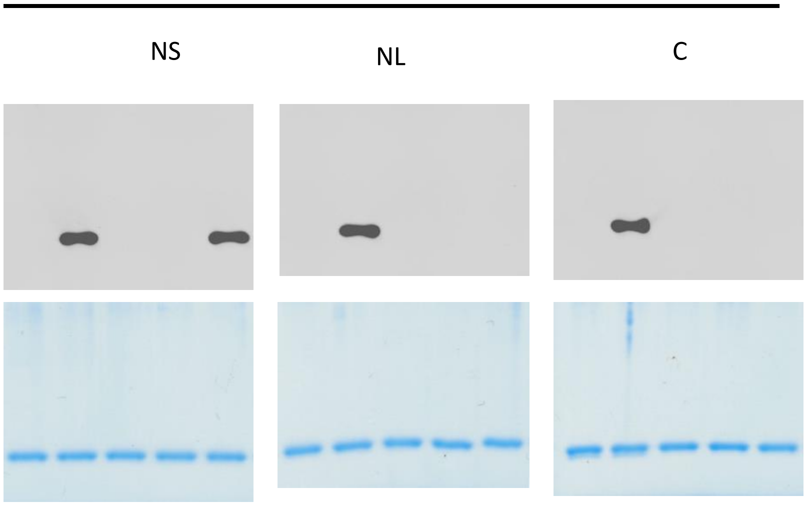

**Fig S3A**

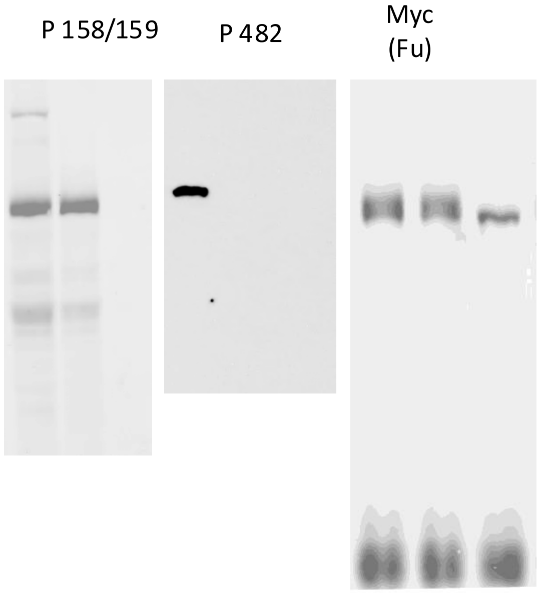


**Fig S3B**

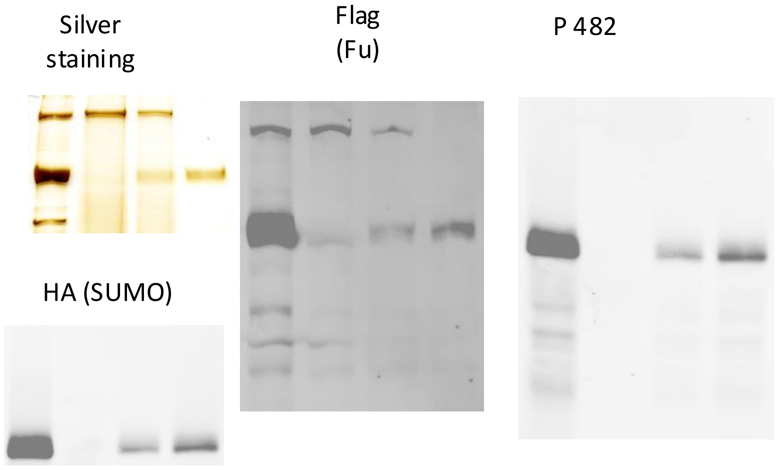


**Fig S3C (BN gel)**

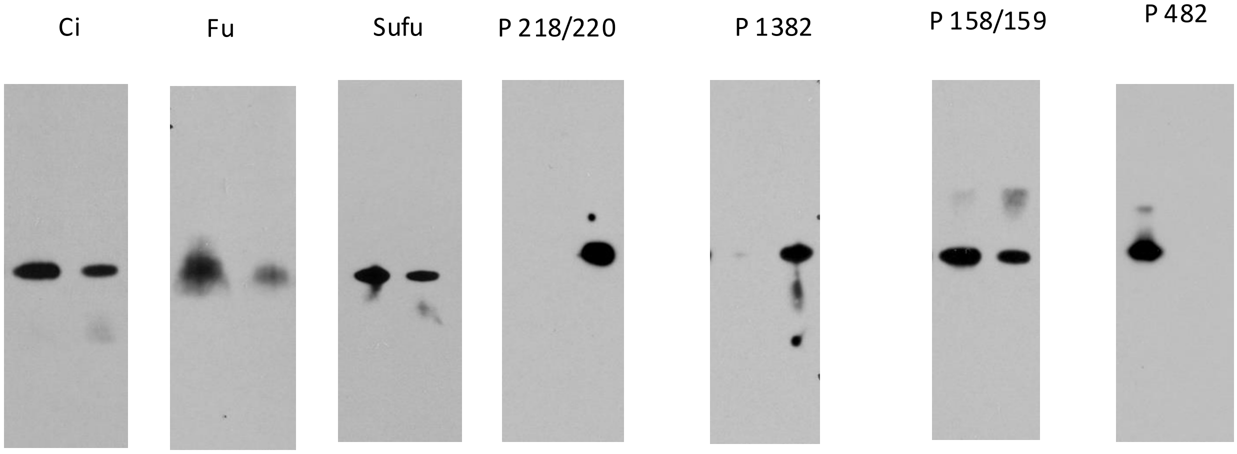


**Fig S3D**

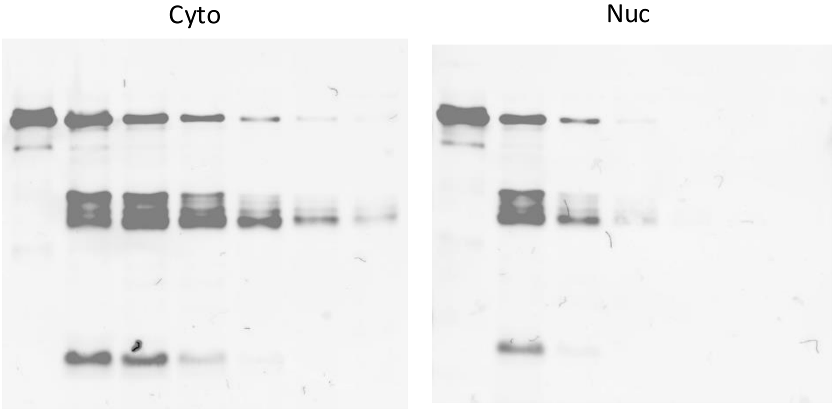

**Fig S3E**

Fu

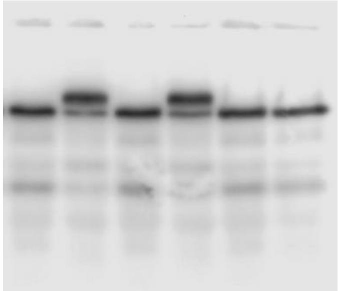

P 158/159

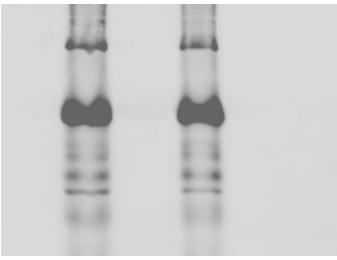

Ci

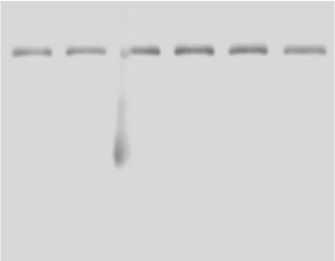

P 218/220

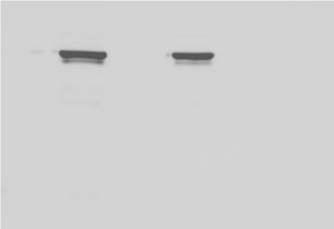

P 1382

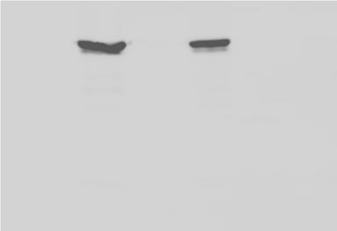

**Fig S3F**

Ci

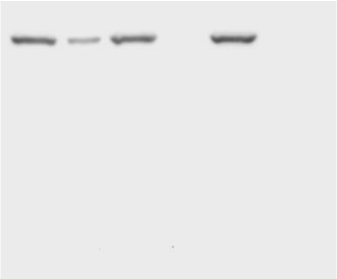

P 218/220

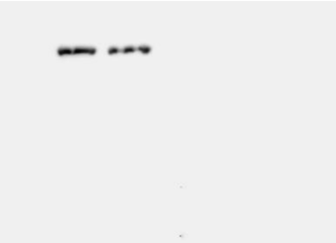

P 1382

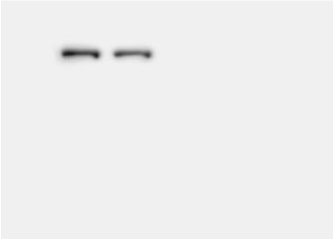

Tubulin

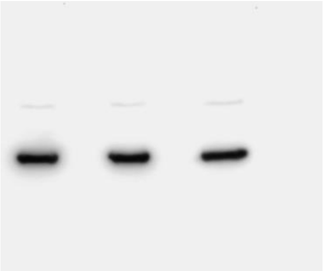

H3

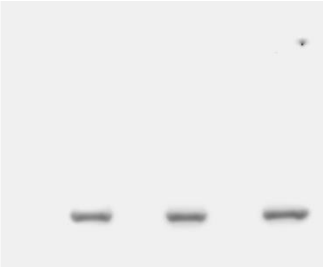

**Fig S4D**

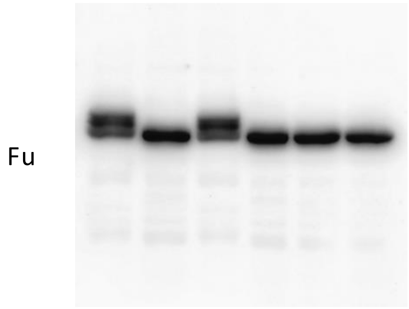


**Fig S4F**

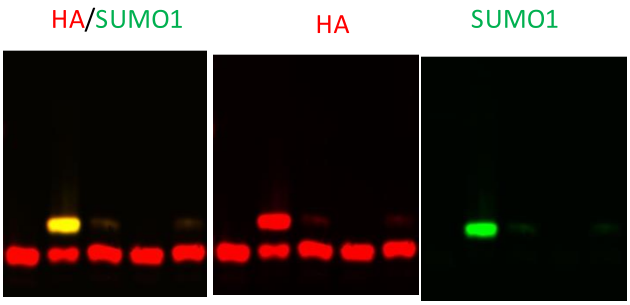


**Fig S4G**

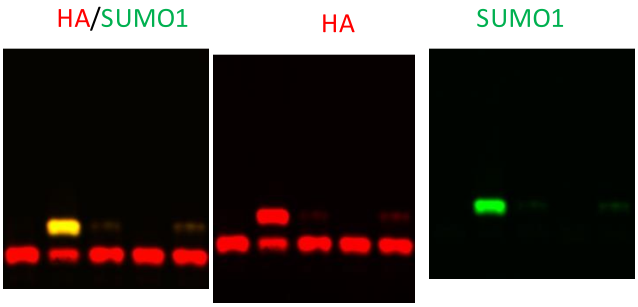

**Fig S5C**

Pre-extraction

S100

P100

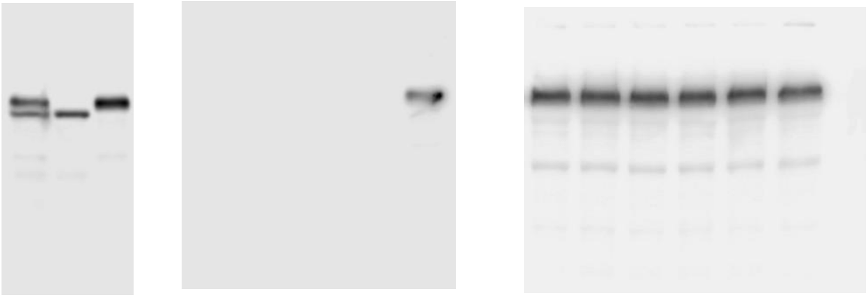

**Fig S5E**

Flag (Fu)



**Fig S5F**

Silver

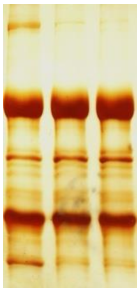

## Fig S6B

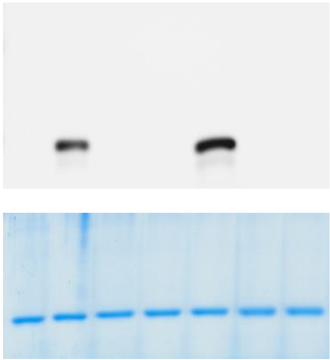

## Fig S6C

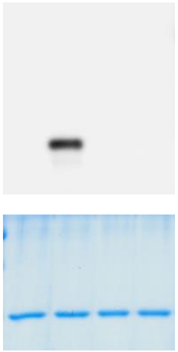

## Fig S6D

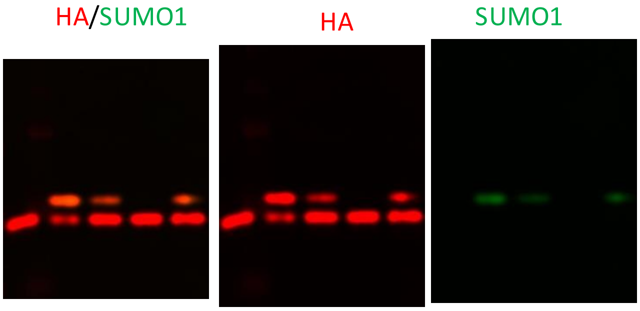


## Fig S6E

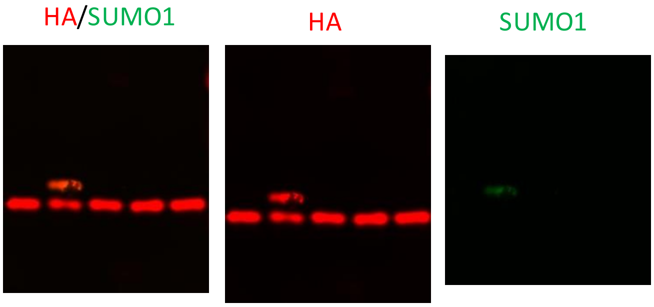


## Fig S6F

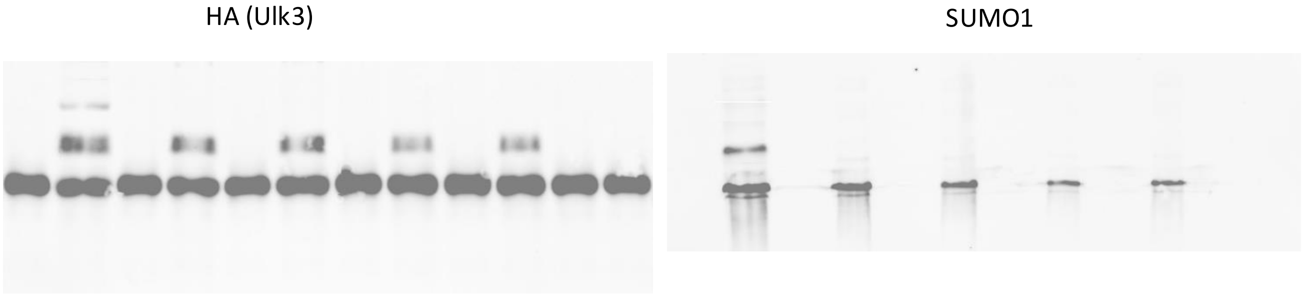


## Fig S6G

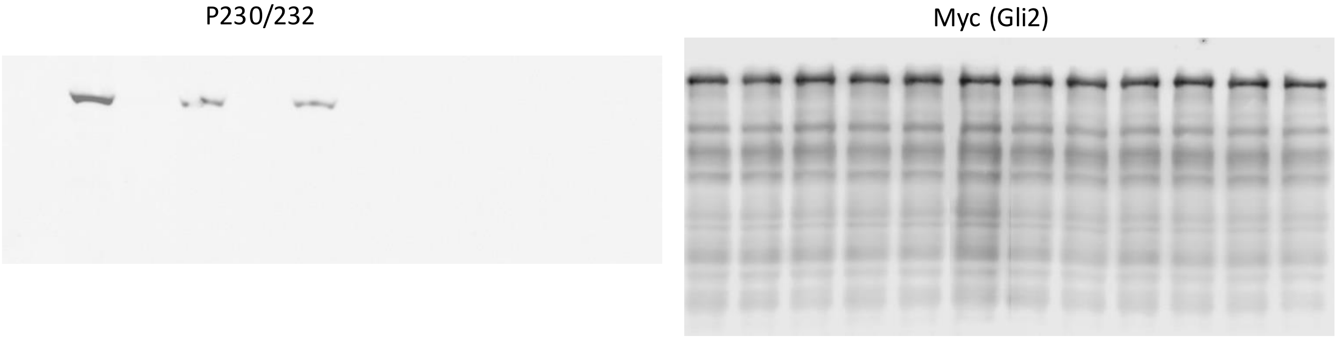

**Fig S6I**

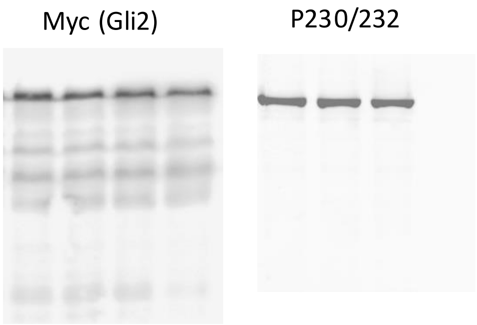


**Fig S6J**

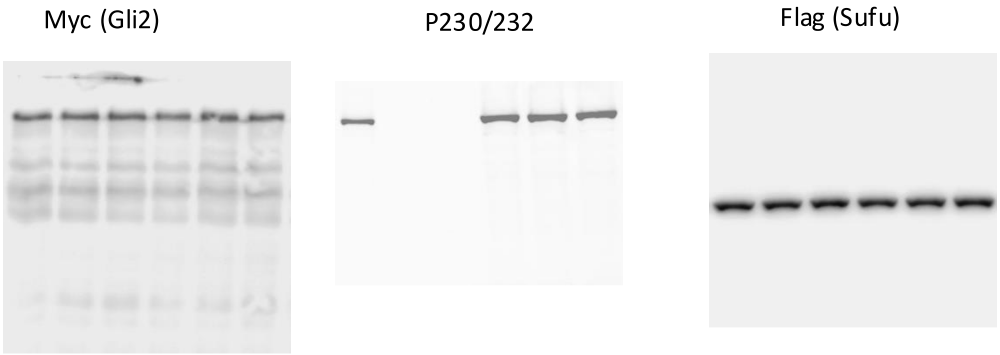


**Fig S6K**

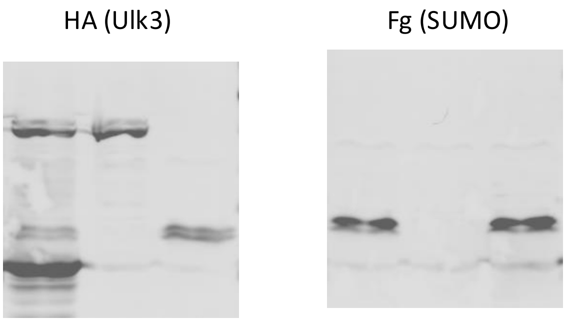

**Fig S7D**

HA (Ulk3)

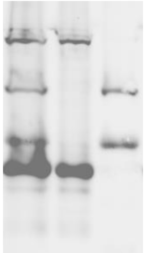

**Fig S7E**

HA (Ulk3)

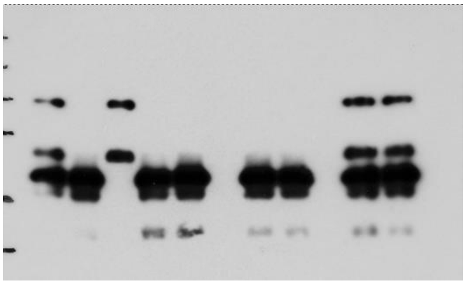

**Fig S7F**

HA (Ulk3)

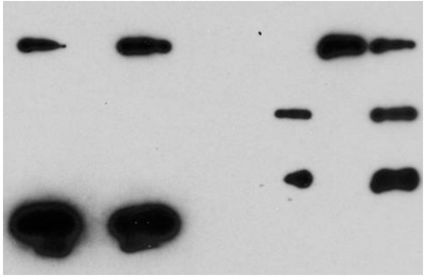

Flag (Gli2)

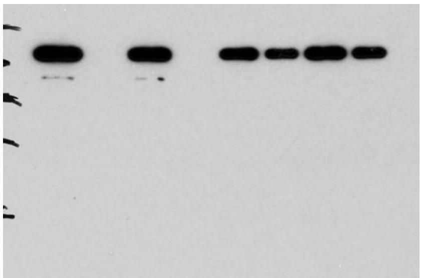

P230/232

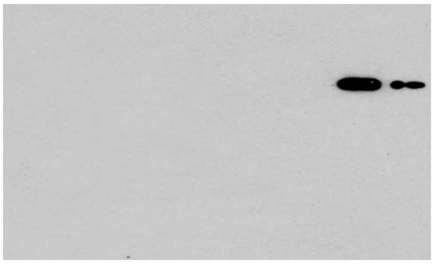

Sufu

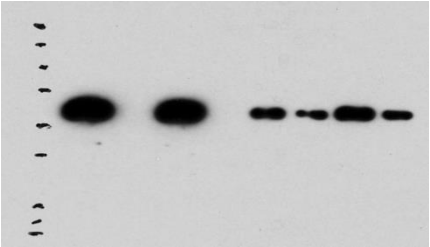

Supplement: Supplementary file 1 — Figs. S1 to S7 Uncropped Western Blots [file sciadv.adq1790_sm.pdf]
